# Gut microbiota diversity after autologous fecal microbiota transfer in acute myeloid leukemia patients

**DOI:** 10.1038/s41467-021-23376-6

**Published:** 2021-05-25

**Authors:** Florent Malard, Anne Vekhoff, Simona Lapusan, Francoise Isnard, Evelyne D’incan-Corda, Jérôme Rey, Colombe Saillard, Xavier Thomas, Sophie Ducastelle-Lepretre, Etienne Paubelle, Marie-Virginie Larcher, Clément Rocher, Christian Recher, Suzanne Tavitian, Sarah Bertoli, Anne-Sophie Michallet, Lila Gilis, Pierre Peterlin, Patrice Chevallier, Stéphanie Nguyen, Emilie Plantamura, Lilia Boucinha, Cyrielle Gasc, Mauricette Michallet, Joel Dore, Ollivier Legrand, Mohamad Mohty

**Affiliations:** 1 Service d’hématologie clinique et de thérapie cellulaire, Hôpital Saint Antoine, APHP, Sorbonne Université, INSERM UMRs 938, Paris, France; 2 Service d’hématologie, Institut Paoli Calmettes, Marseille, France; 3 Service d’hématologie, Centre Hospitalier Lyon Sud, Hospices Civils de Lyon, Lyon, France; 4 CHU de Toulouse, Institut Universitaire du Cancer de Toulouse Oncopole, Université de Toulouse III Paul Sabatier, Service d’hématologie, Toulouse, France; 5 Service d’hématologie, Centre Léon Bérard, Lyon, France; 6 Service d’hématologie, CHU Nantes, Nantes, France; 7 Service d’hématologie clinique, Hôpital de la Pitié Salpétrière, APHP, Sorbonne Université, Paris, France; 8 MaaT Pharma, Lyon, France; 9 Université Paris-Saclay, INRAE, MetaGenoPolis, AgroParisTech, MICALIS, Jouy-en-Josas, France

**Keywords:** Phase II trials, Acute myeloid leukaemia

## Abstract

Acute myeloid leukemia (AML) intensive chemotherapy combined with broad-spectrum antibiotics, leads to gut microbiota dysbiosis promoting pathological conditions and an increased incidence of complications. Here we report findings from a phase II single-arm, multicenter study evaluating autologous fecal microbiota transfer (AFMT) in 25 AML patients treated with intensive chemotherapy and antibiotics (ClinicalTrials.gov number: NCT02928523). The co-primary outcomes of the study are to evaluate the efficacy of AFMT in dysbiosis correction and multidrug-resistant bacteria eradication. The main secondary outcomes are to define a dysbiosis biosignature, to evaluate the effect of dysbiosis correction on patient clinical status, to assess the short and mid-term safety of AFMT in this immunocompromised population, and to evaluate the feasibility of the AFMT procedure and acceptability by the patient. Intensive induction chemotherapy induces a dramatic decrease of α-diversity indices, and a microbial dysbiosis with a significant shift of the microbial communities and domination of pro-inflammatory families. After AFMT treatment, α-diversity indices return to their initial mean levels and the similarity index shows the restoration of microbial communities. The trial meets pre-specified endpoints. AFMT appears to be safe and may be effective for gut microbiota restoration in AML patients receiving intensive chemotherapy and antibiotics, with an excellent gut microbiota reconstruction based on both richness and diversity indices at the species level.

## Abstract (web summary)

The combination of chemotherapy and broad-spectrum antibiotics induces gut microbiota (GM) dysbiosis in acute myeloid leukaemia (AML) leading to additional complications. Here, the authors report the efficacy in GM restoration and safety of autologous faecal microbiota transfer in treated AML patients in a phase II clinical trial.

## Introduction

Acute myeloid leukemia (AML) is a rare but potentially fatal blood cancer. The standard treatment for AML relies on conventional chemotherapy with or without allogeneic hematopoietic cell transplantation (alloHCT). Eligible patients first undergo an intensive induction chemotherapy (IC). If complete remission is achieved, consolidation therapy is performed to deepen response and achieve long-lasting remission. Several intensive chemotherapy cycles may be necessary. Finally, depending on the patient risk profile, HCT is undertaken^1^. The different treatment phases of AML require prolonged hospital stays in a protected environment and multiple courses of antibiotic treatment, due to the high risk of life-threatening infectious complications^2,3^.

Such treatments have been demonstrated to dramatically alter the rich and diverse composition of the human gut microbiota in AML patients^4–6^. The induced so-called dysbiosis is characterized by a reduction of overall microbial diversity, a disruption of beneficial bacteria which support host defenses, and a rise in dominance of bacterial species usually subdominant, including some pathogens, pathobionts, and multidrug-resistant bacteria (MDRB)^7,8^. The use of carbapenems during IC is associated with 4 times increased the risk of developing an infection within the following 90 days^6^. Thus, chemotherapy and antibiotic treatment disrupt the mutualistic relationship between host and microorganisms and promote pathological conditions involving impaired gut barrier function, uncontrolled local immune responses and, potentially, systemic inflammation^9^.

Moreover, the IC is known to have dramatic consequences on the gastrointestinal epithelium, which can lead to colitis with severe abdominal pain, diarrhea, and/or hematochezia^10,11^. The intestinal consequences of AML treatments can, thus, interfere with the patient’s optimal care through the increase of infectious-related complications, bad nutrition status, longer duration of hospitalization, interruption, or delayed consolidation courses due to treatment toxicity and MDRB carriage preventing alloHCT^12,13^.

Recent studies demonstrated that a high gut microbial diversity is associated with improved clinical outcome and reduced infectious complications in AML patients^4,5^. The development of strategies such as fecal microbiota transfer (FMT) to restore the diverse microbial communities lost during disease treatment and, consequently, to suppress, decrease or even prevent – in the case of subsequent alloHCT - treatment-related complications in AML patients, could offer novel therapeutic possibilities^14,15^. Here we report the results of a single-arm study evaluating autologous FMT (AFMT) in AML patients treated with intensive chemotherapy and antibiotics. In this work, AFMT is safe, reduces the count of antibiotic resistance genes and may be effective for gut microbiota restoration in patients receiving intensive chemotherapy and antibiotics for AML, with an excellent gut microbiota reconstruction based on both richness and diversity indexes at the species level. Our findings suggest that therapeutic manipulation of gut microbiota may become a treatment option to restore gut microbiota diversity and potentiate cancer management.

## Results

### Patient characteristics

A total of 62 AML patients were screened in 7 different centers, 25 were treated with AFMT, and 20 were considered as the per-protocol population. The baseline characteristics of treated and per-protocol patients are listed in Table 1 (see data for screen-failed patients in Supplementary Table 1). There were more men than women in the per-protocol population (ratio 3:1) and the median age was 50 years (range, 24–68). Most patients (80% of both treated and per-protocol patients) were considered as being intermediate-risk AML while 3 and 2 patients of the treated population were from the favorable and unfavorable risk groups, respectively. All patients received intensive IC with cytosine arabinoside associated with an anthracycline, except in one patient where anthracycline was replaced by gemtuzumab ozogamicin. No prophylactic antibiotic was used during IC, however all patients but one received antibiotic with large anti-anaerobic coverage (either piperacillin-tazobactam or imipenem-cilastatin) for febrile neutropenia during IC. The last patient did not receive antibiotic for febrile neutropenia.

**Table 1:** Baseline demographics and clinical characteristics of treated and per-protocol patients.

		Treated patients (*n* = 25)	Per-protocol patients (*n* = 20)
Gender, *n* (%)	Male Female	18 (72%) 7 (28%)	15 (75%) 5 (25%)
Age at inclusion (years)	Median [range]	52 [24–68]	50 [24–68]
BMI at inclusion	Median [Range]	26.33 [19.72–41.34]	26.54 [21.24–41.34]
Induction chemotherapy, *n* (%)	Cytarabine + Idarubicin Cytarabine + Daunorubicin Cytarabine + gemtuzumab ozogamicin	13 (52%) 11 (44%) 1 (4%)	12 (60%) 7 (35%) 1 (5%)
Antibiotics during induction chemotherapy, *n* (%)	Piperacillline-tazobactam alone Piperacillline-tazobactam followed by imipenem-cilastatin or meronem	12 (48%) 11^a^ (44%)	9 (45%) 9^a^ (45%)
	Cefepim followed by imipenem-cilastatin No antibiotic	1 (4%) 1 (4%)	1 (5%) 1 (5%)
Consolidation chemotherapy, *n* (%)	Cytarabine Cytarabine + Idarubicin Cytarabine + Methotrexate Cytarabine + Rituximab Unknown	19 (76%) 2 (8%) 2 (8%) 1 (4%) 1 (4%)	14 (70%) 2 (10%) 2 (10%) 1 (5%) 1 (5%)
Leukemia Risk category (revised MRC classification), *n* (%)	Favorable Intermediate Unfavorable	3 (12%) 20 (80%) 2 (8%)	2 (10%) 16 (80%) 2 (10%)
FLT3-ITD mutation, *n* (%)	No Yes Not Determined	2 (11%) 16 (89%) 7	2 (12%) 14 (88%) 4
NPM1 Mutation, *n* (%)	No Yes	13 (52%) 12 (48%)	9 (45%) 11 (55%)
CEBPA Mutation, *n* (%)	No Yes	11 (44%) 14 (56%)	7 (35%) 13 (65%)
Karyotype, *n* (%)	Normal Abnormal Failure	13 (52%) 11 (44%) 1 (4%)	10 (50%) 9 (45%) 1 (5%)

*BMI* body mass index.

^a^Three patients received meronem, others received imipenem-cilastatin.

### Feasibility and acceptability of the AFMT procedure

**Fig. 1:**
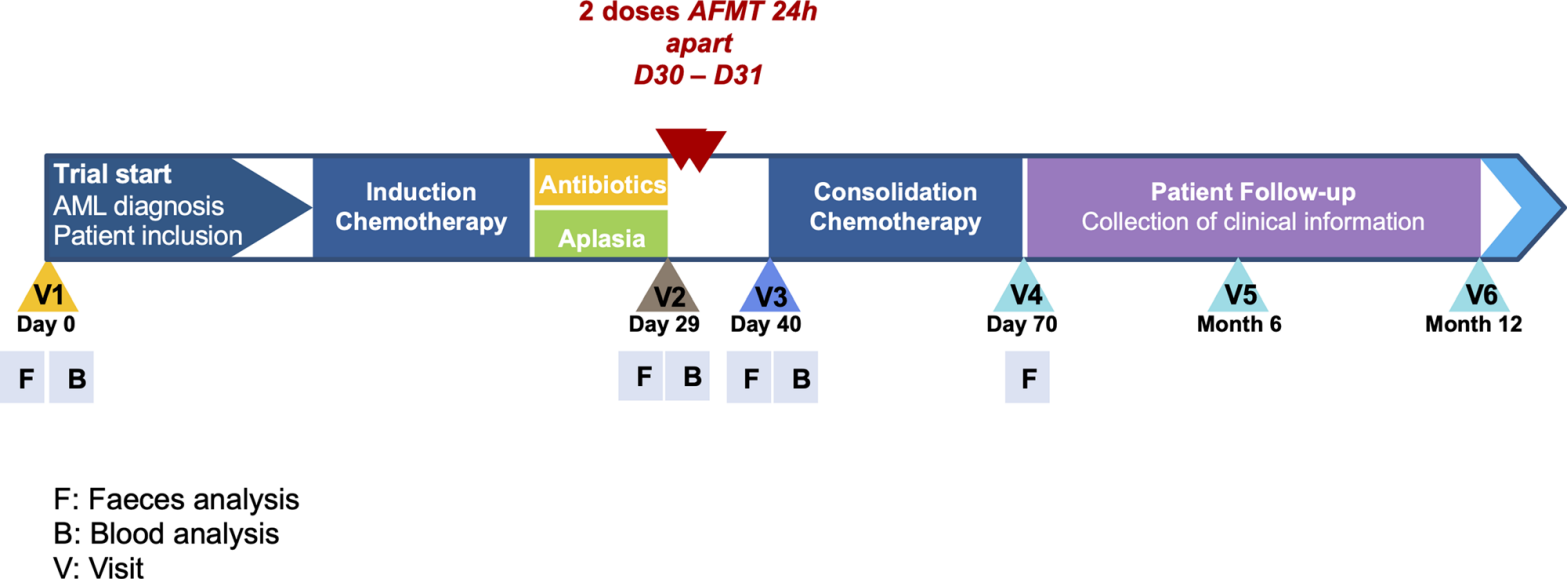
Study flow chart. AML is for acute myeloid leukemia. AFMT, autologuous fecal microbiota transfer; D, day.

AFMT feasibility and acceptability were evaluated as a secondary outcome. Twenty-eight patients fulfilled all inclusion and exclusion criteria, three eligible patients refused the AFMT procedure and 25 patients were treated and received the two AFMT one day apart. Nevertheless, only 20 patients were able to receive the two AFMT as planned after IC and hematopoietic recovery from aplasia (See Fig. 1 for study flow chart). For the remaining five patients, AFMT was not feasible right after the IC and was delayed after the first consolidation chemotherapy in four patients [AFMT contraindication in two patients (anal fistula (*n* = 1) and colitis (*n* = 1)), logistical organization in 2 others] or delayed 35 days after hematopoietic recovery from aplasia, in one patient, due to an infectious event requiring prolongation of antibiotics.

Median hemoglobin level, leukocytes count, absolute neutrophil count (ANC), lymphocytes count and platelets count were 9.35 g/dL (IQR, 8.85–10.1), 4.8 G/L (IQR, 2.0–8.0), 1.78 G/L (IQR, 0.75–4.7), 0.88 G/L (IQR, 0.65–1.12) and 66 G/L (IQR, 31.5–246) respectively, at time of AFMT (Supplementary Fig. 1). The median AFMT product retention time was longer than expected (188.50 min [range 48–288] and 138 min [range 41–245] for the first and second AFMT, respectively, instead of the recommended 120 min) demonstrating the feasibility of the enema procedure and the absence of discomfort for the patients.

In order to assess the acceptability of the procedure, treated patients’ quality of life (*n* = 25) was evaluated throughout the clinical study. The data showed that the results of the questionnaire after AFMT (V3) were similar or tended to improve, compared to those at V2 before AFMT (especially self-care, usual activities and anxiety, and depression parameters), which highlights the absence of a negative impact of AFMT on the overall health of patients (Supplementary Table 2).

### Gut microbiota reconstitution with AFMT

**Fig. 2:**
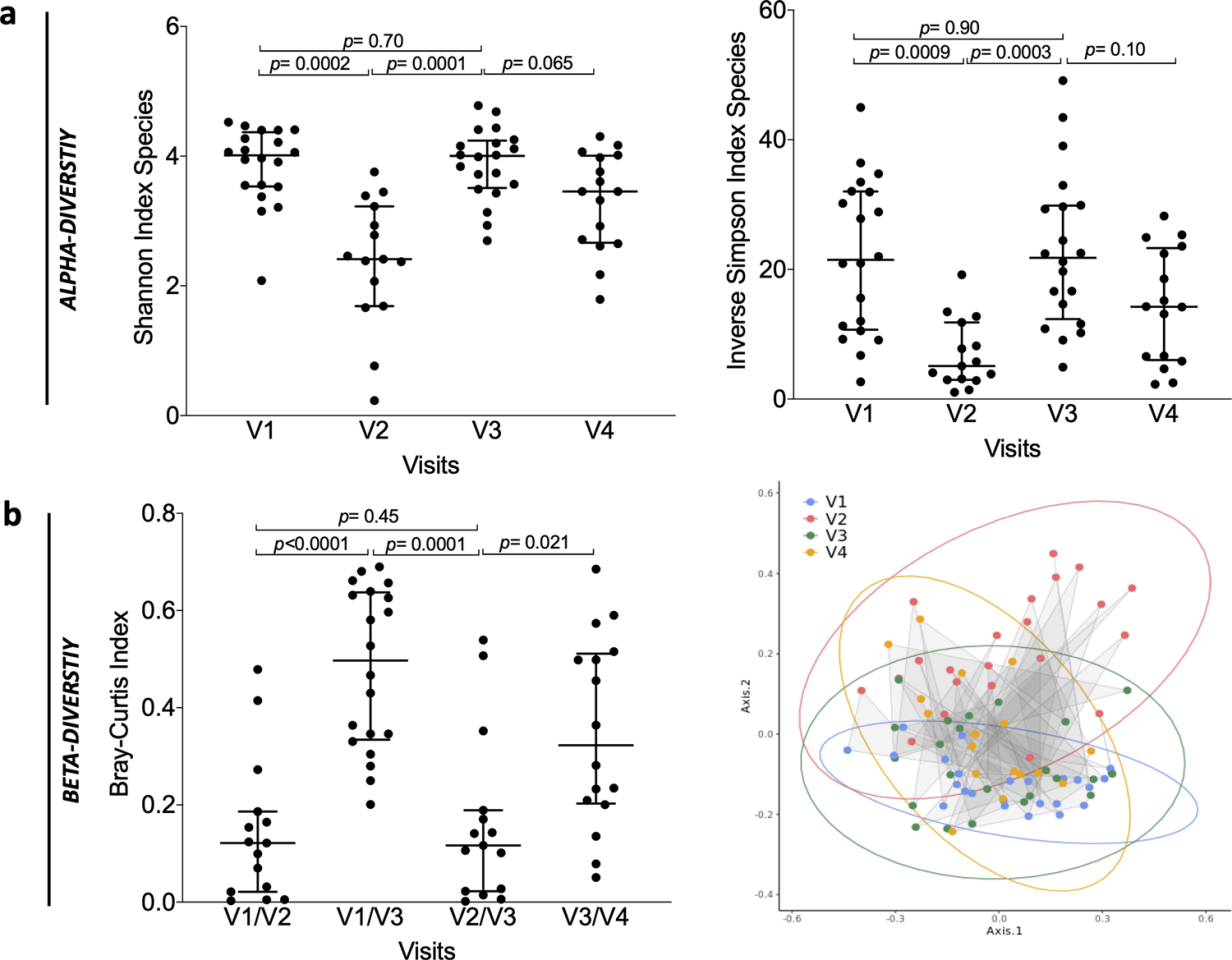
Characterization of the fecal microbiota from visit 1 (V1) to visit 4 (V4) in per-protocol patients (*n* **=** 20). **a** Alpha-diversity measured at species level with, from left to right: Shannon and Inverse Simpson indexes (*P*-value were determined by two-sided signed-rank Wilcoxon paired test, no adjustments were made for multiple comparisons, error bars indicate median and interquartile range). **b** Beta-diversity measured at species level with the Bray–Curtis index comparison between visits and Principal Coordinates Analysis based on Bray–Curtis similarity at each visit (*P*-value were determined by two-sided signed-rank Wilcoxon paired test, no adjustments were made for multiple comparisons, error bars indicate median and interquartile range). Source data are provided as a Source Data file.

**Fig. 3:**
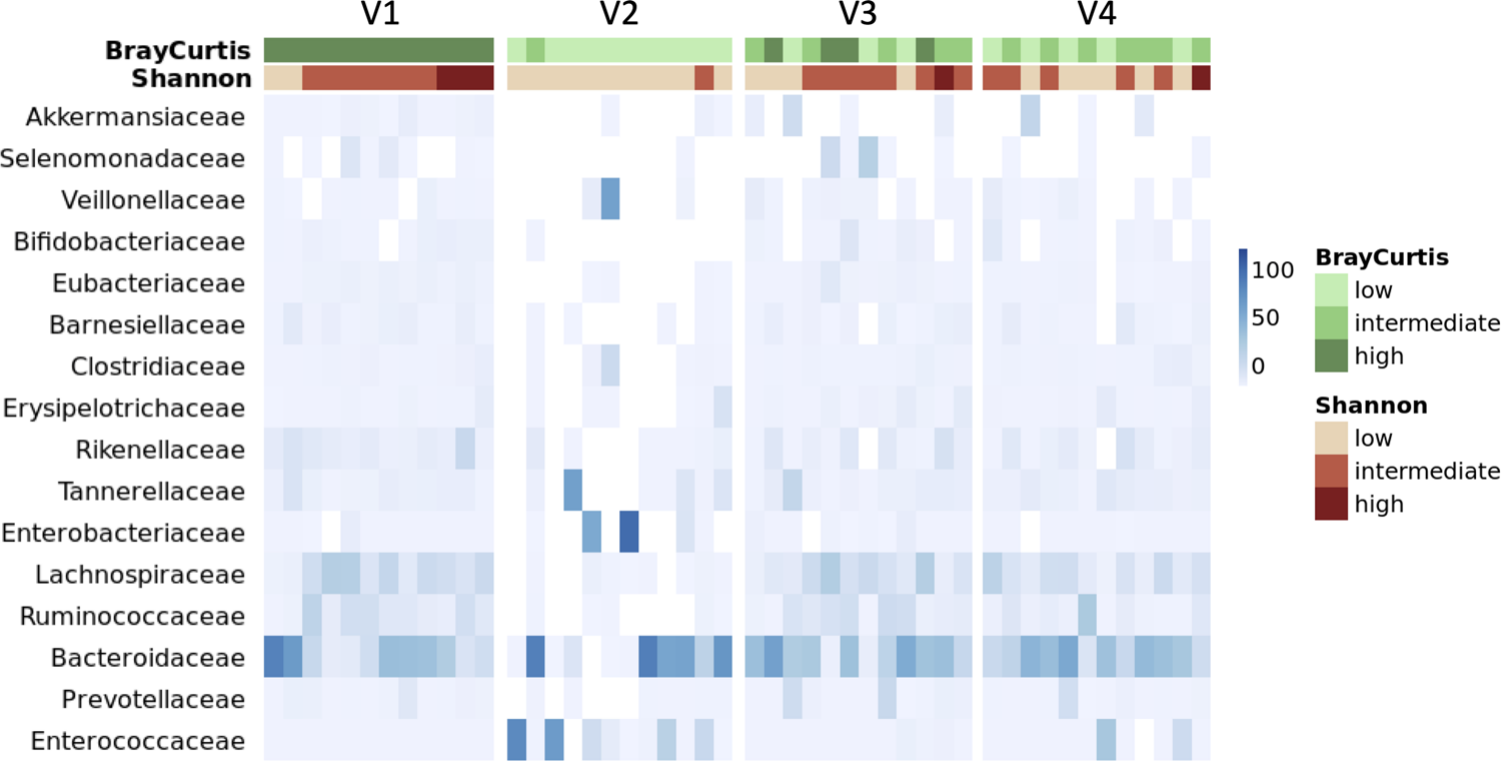
Family profile heatmap. The relative abundances of the families found in the different samples are represented in shades of blue. Each column corresponds to a patient and the order of the columns is respected between the different panels. Only patients for which a complete kinetics from visit 1 (V1) to visit 4 (V4) is available are shown (*n* = 12). For each sample, the associated values of the Shannon index at the species level and the Bray-Curtis similarity with respect to V1 at the species level are represented. For Shannon index, ranges were based on the Shannon value of the study population: low < first quartile, medium ≥ first quartile and < third quartile, high ≥ third quartile. For Bray–Curtis similarity index: low < 0.4, medium ≥ 0.4 and < 0.6 and high ≥ 0.6. The choice of this mathematically determination was made before the analysis of the results.

Evaluation of AFMT efficacy in dysbiosis correction by measure of microbiota diversity was evaluated as a co-primary outcome. We first examined the impact of IC and subsequent AFMT treatment on the metagenomic richness and diversity of fecal microbiota in per-protocol patients. We demonstrated that IC induces a dramatic and significant decrease of richness based on α-diversity indexes, which reflect the number of unique bacteria taxa present and their relative abundances, between V1 (baseline, before IC) and V2 (after IC and before AFMT), at the metagenomic species level: median Shannon index 4.0 versus 2.4; (*p* = 0.0002) and median inverse Simpson index 21.5 versus 5.1 (*p* = 0.0009) respectively (Fig. 2a). Similar observations were made at the gene level: median richness 548,529 versus 81,980 (*p* < 0.0001) (Supplementary Fig. 2). Measure of β-diversity with the Bray–Curtis similarity index (BC), which allows a comparative assessment of bacterial diversity between samples for each participant, demonstrated the induction of a microbial dysbiosis with a significant shift of the microbial communities after IC (median BC V1–V2: 0.12) (Fig. 2b) and domination of pro-inflammatory families (*Enterobacteriaceae*, *Enterococcaceae* and *Veillonellaceae*) (Fig. 3 and Supplementary Fig. 3). After AFMT treatment, α-diversity indexes returned to their initial median levels when compared to V2 (median Shannon: 4.0, *p* = 0.0001; median Inverse Simpson: 21.8, *p* = 0.0003) with no significant difference between values at V1 and V3 (Fig. 2a). The number of genes was significantly increased but did not return to its baseline level (median richness: 389,768, *p* < 0.0001 when compared to V2) (Supplementary Fig. 3). Thus, the gut microbiota at V3 after AFMT is reconstructed based on diversity indexes at the species level. Indeed, the BC similarity index showed the restoration of microbial communities after AFMT treatment, in particular for the families *Lachnospiraceae* and *Ruminococcaceae*, which composition was closer to that of the initial communities at the species level (median BC V1–V3: 0.50) (Figs. 2b, 3). We then observed that the gut microbiota diversity at V4 was not altered by high-dose cytarabine based consolidation chemotherapy, with no significant reduction of α-diversity indexes and a slight reduction of gene richness compared to V3 (median Shannon: 3.5; Inverse Simpson: 14.2; gene richness: 270,689, *p* = 0.039) (Fig. 2a and Supplementary Fig. 2). Finally, we observed that among the 19 per-protocol patients that received antibiotics, patients’ receiving carbapenems (imipenem-cilastatin or meronem) have decreased α-diversity indexes at V2 compared to patients receiving piperacillline-tazobactam alone, that persist at V3 after AFMT (Supplementary Fig. 4).

### MDRB decolonization

**Fig. 4:**
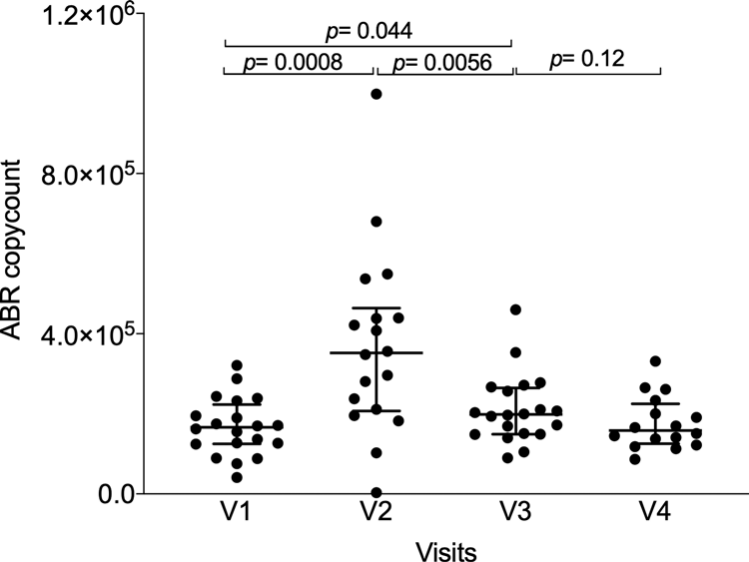
Number of reads mapped against antibiotic resistance genes identified through metagenomic sequencing from V1 to V4 for the per-protocol population (*n* **=** 20). *P*-values were determined by two-sided signed-rank Wilcoxon paired test, no adjustments were made for multiple comparisons, error bars indicate median and interquartile range. ABR is for antibiotic resistance genes. Source data are provided as a Source Data file.

Evaluation of AFMT efficacy in MDRB eradication was evaluated as a co-primary outcome. To evaluate the presence of antibiotic resistance through metagenomic sequencing, we mapped the total sequencing reads on the MEGARes antibioresistance gene database. We found evidence that IC and associated antibiotic treatments induced a significant increase in the median number of reads mapped against antibiotic resistance genes from V1 to V2 (166,158 versus 352,140 reads, *p* = 0.0008) for per-protocol patients (Fig. 4). Thereafter, a significant reduction of the median number of reads mapped was observed at V3 after AFMT (198 374 reads, *p* = 0.0056). The count of antibiotic resistance genes at V4 (158,229 reads) was also not significantly different compared to that in V3, suggesting that AFMT had a beneficial effect in alleviating the microbiota disruption induced by consolidation chemotherapy and antibiotics (Fig. 4).

### Adverse events

Adverse events were collected to evaluate the safety of AFMT (secondary outcome). During the AFMT treatment, no harmful changes in vital signs of treated patients (*n* = 25) were observed (heart rate, blood pressure) (Supplementary Table 3). In the first 24 h after AFMT, five adverse events (AEs) were reported in four patients (16%) (Supplementary Table 4). Two were considered as related to the procedure, but neither required treatment for both, patients recovered without sequelae: one patient had moderate abdominal pain for 6 days and one patient had mild diarrhea for 5 weeks, both starting the day after the second AFMT. The other three AEs also reported within 24 h after AFMT were a mild transient fever (38.1 °C), diarrhea, and weight increase in each of three different patients. Regarding the weight increase, the patient gained 6 kg between V2 and V3. Of note, this patient had a poor nutritional status at the time of AFMT with a weight loss of 12 kg during IC (between V1 and V2). No serious adverse events (SAEs) were reported during this period in the treated population.

After the first 24 h post-AFMT and until the end of the 1-year follow-up period, 415 AEs were reported in 24 of the 25 treated patients (96%) (Supplementary Table 5). Among them, one patient had mild mucosal inflammation for 8 days, starting 103 days after AFMT, which was considered to be probably related to the procedure. Other AEs were in line with leukemia patient profiles receiving intensive chemotherapy. Most AE occurred between inclusion and AFMT (V1–V2) (incidence rate of 27% per 100 person-days) and between V3 and V4 (incidence rate of 15% per 100 person-days) (Supplementary Fig. 5).

In addition, SAEs were reported, mainly between inclusion and AFMT (V1–V2) (15 SAEs in five patients, incidence rate of 1.57% per 100 person-days) and between V3 and V4 (11 SAEs in three patients, incidence rate of 0.90% per 100 person-days) (Supplementary Fig. 5 and Supplementary Table 6). None of these SAEs occurred during the first month following AFMT. Only one SAE was declared to be possibly related to the AFMT treatment by the site investigator. Asymptomatic colonization by multidrug resistant *E. coli* was detected in the patient’s feces at V3, 22 days after the second AFMT. Thereafter, the patient developed *Escherichia coli* sepsis 93 days after the second AFMT with hyperthermia and gastrointestinal symptoms, and fully recovered with antibiotic therapy. Importantly multidrug resistant *E. coli* was not detected during screening of the AFMT product.

Finally, given AML patients have a high infectious burden during consolidation post-intensive chemotherapy^16^, we decided to evaluate the impact of AFMT on infectious AE. During the consolidation chemotherapy, only six patients had a microbiologically defined infection (MDI), one a clinically defined infection (CDI), eight had fever of unknown origin and five had no fever. There was no difference in the microbiota diversity after AFMT between these three groups (Supplementary Fig. 6). Regarding use of antibiotics during consolidation chemotherapy, we find that only half patients (*n* = 10) received antibiotic with large anti-anerobic coverage (imipenem-cilastatin, *n* = 3; piperacillline-tazobactam, *n* = 7), while five patients received third or fourth generation cephalosporine and five patients did not received antibiotics. Interestingly, patients receiving antibiotic with a large anti-anerobic coverage had a significantly lower alpha diversity after AFMT, compared to patients that received antibiotic with less anti-anerobic coverage or no antibiotics (Supplementary Fig. 6).

### Inflammatory parameter

**Fig. 5:**
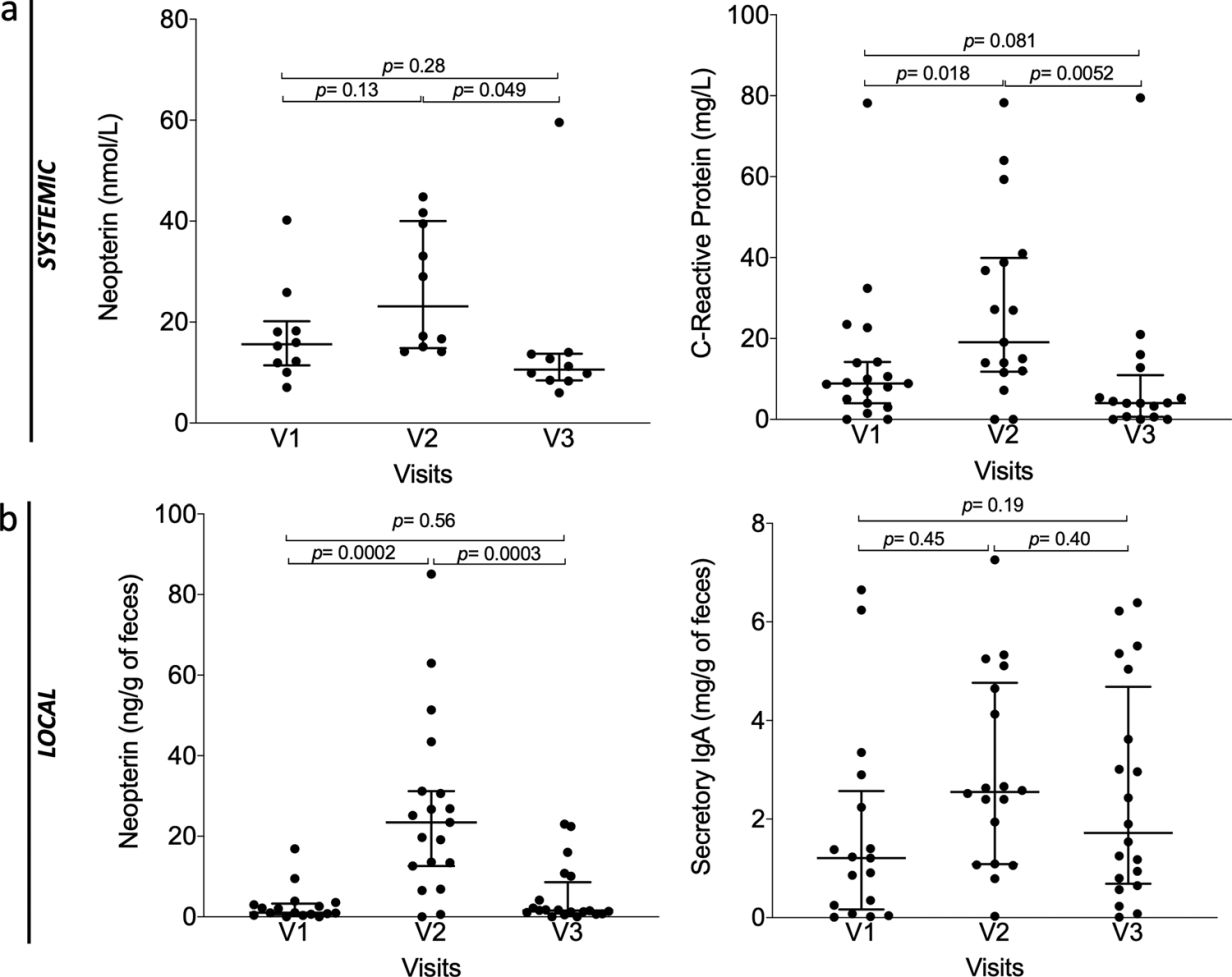
Evolution of biochemical and immunological parameters in per-protocol patients (*n* **=** 20). **a** Systemic level in blood, from left to right: neopterin, C-Reactive Protein (*P*-value were determined by two-sided signed-rank Wilcoxon paired test, no adjustments were made for multiple comparisons, error bars indicate median and interquartile range, for neopterin *n* = 10). **b** Local levels in feces: neopterin, secretory immunoglobulin A (IgA, *P*-value were determined by two-sided signed-rank Wilcoxon paired test, no adjustments were made for multiple comparisons, error bars indicate median and interquartile range). Source data are provided as a Source Data file.

In addition, in order to evaluate the safety of AFMT (secondary outcome), we also measured inflammatory parameters both locally in the gut and systematically in plasma of per-protocol patients (*n* = 20) during the first three visits (Fig. 5 and Supplementary Fig. 7). As expected after intensive chemotherapy, we observed a significant increase of systemic C-Reactive Protein at V2 (Median V1: 8.9 mg/L; V2: 19.1 mg/L; *p* = 0.018) and a return to baseline at V3 after AFMT (Median V3: 4.05 mg/L; V2 versus V3: *p* = 0.0052). Systemic neopterin levels followed the same variations with an increase at V2 and a return to baseline at V3 (Fig. 5a).

We next sought to assess the local immunity and inflammation in the gut by measuring fecal neopterin and secretory IgA (Fig. 5b). Neopterin is produced and released from activated macrophages upon stimulation with various inducers, such as bacterial components, and through IFNγ, TNFα pathways^17^. Neopterin reflects the degree of cell-mediated immune response and, thereby, the levels of intestinal inflammation. We observed a significant increase of median fecal neopterin levels after IC (V1: 1.05 ng/g of feces versus V2: 23.45 ng/g of feces; *p* = 0.0002), highlighting the expected inflammatory intestinal status of patients after IC and antibiotic therapy. Levels were significantly decreased and returned to baseline following AFMT (V3: 1.58 ng/g of feces; *p* = 0.0003 compared to V2). These variations were in line with C-Reactive Protein variations. As a mirror of local immunity, secretory IgA was also measured in feces, and similar trends were observed (Median V1: 1.21 mg/g of feces; V2: 2.55 mg/g of feces; V3: 1.72 mg/g of feces). Altogether, these data clearly point out to the absence of deleterious inflammatory reaction, both locally and systematically, after AFMT.

Previous studies have reported that oxidative stress is closely related to the occurrence and development of cancers^18^ and is also associated with gut dysbiosis. Oxidative stress occurring during inflammation is a factor amplifying dysbiosis by strongly decreasing the microbial diversity in the gut, and by promoting the outgrowth of specific aerotolerant and proinflammatory bacterial taxa^19^. Therefore, maintaining a functional antioxidant response is important. We measured the total antioxidant status (TAS), and we observed no significant variation of TAS levels between V1 and V2 (Median V1: 1.35 mmol/L; V2: 1.30 mmol/L), but a significant increase after AFMT (V3: 1.44 mmol/L; *p* = 0.0026), which could be associated with gut microbiota restoration (Supplementary Fig. 5).

### Correlation between host and microbiota parameters

**Fig. 6:**
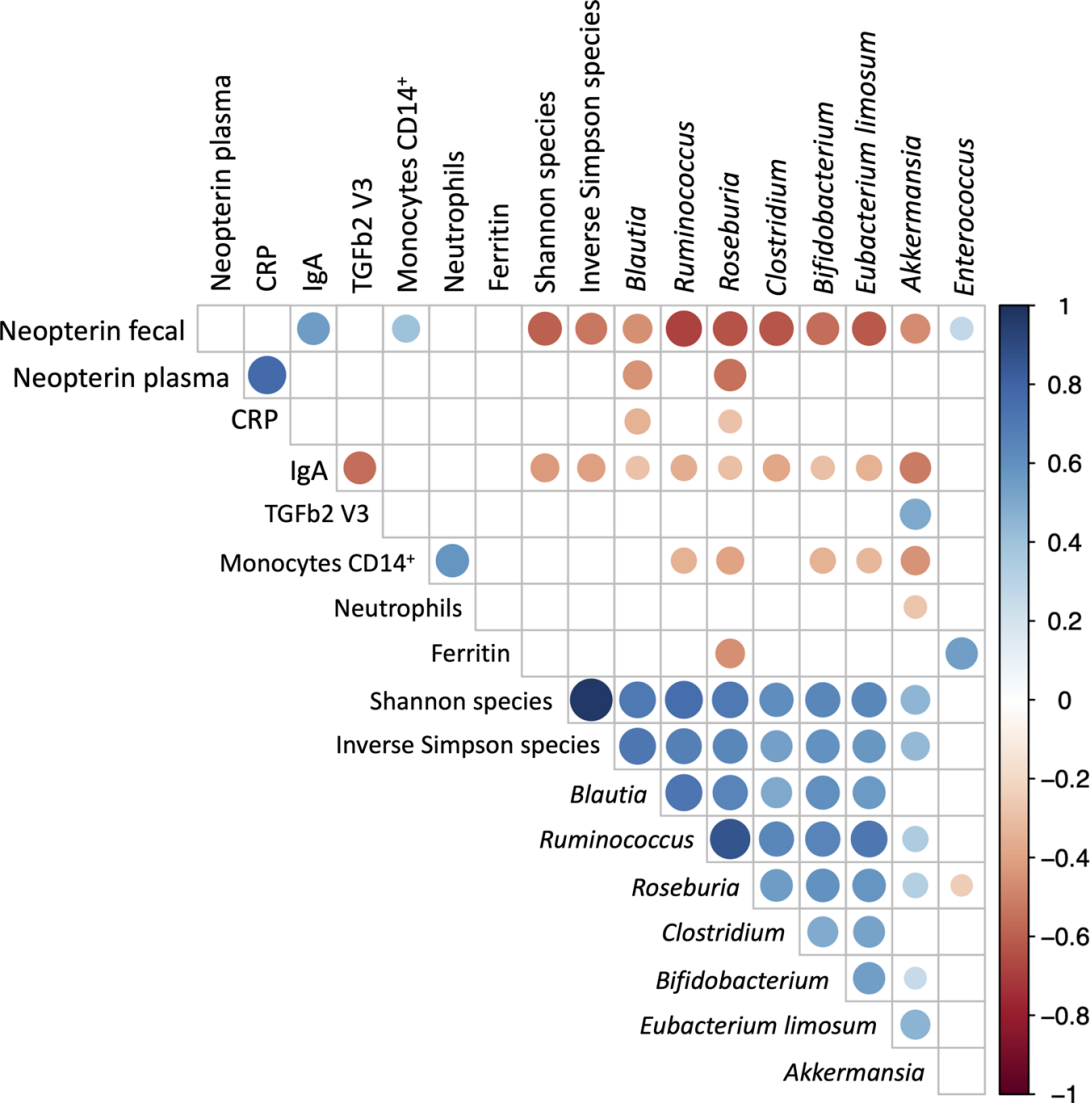
Correlation between host and microbiota parameters (*n* **=** 20). Spearman pairwise correlation. Only significant correlations are depicted. Blue = positive correlation; Red = negative correlation. Correlations are calculated from the values measured in all patients at all visits (except for TGFβ2 for which only the values measured at visit V3 are considered). CRP is for C-Reactive Protein; IgA, immunoglobulin A; TGFβ2_V3, Transforming growth factor-beta 2 at visit 3; CD14_pos, cluster of differentiation 14 positive. Source data are provided as a Source Data file.

Finally, in order to establish a dysbiosis biosignature (secondary outcome), we sought to investigate the link between microbiota parameters (e.g. abundance of specific taxa and alpha-diversity indices), immune and inflammatory parameters (neopterin, IgA, IL-10, CRP….) by performing a Spearman correlation analysis, all visits combined (Fig. 6). We focus on bacteria with an already evidenced role in patients with hematologic malignancies^20^, including butyrate producing bacteria (*Blautia, Rumminococcus, Roseburia, Clostridium and Eubecterium Limosum*), *Bifidobacterium* that cross-fed butyrate, *Akkermensia* and *Enterococcus*. We find a strong negative correlation between fecal neopterin and alpha diversity, butyrate producing bacteria and *Bifidobacterium*. Similarly, some butyrate producing bacteria correlate negatively with other inflammatory parameters, including CRP, ferritin and CD14+ monocytes. Furthermore, IgA, that is associated with inflammation and gut microbiota dysbiosis in irritable bowel syndrome, also correlate negatively with butyrate producing bacteria in you study. In contrast the pro-inflammatory bacteria *Enterococcus* correlates positively with fecal neopterin and ferritin and negatively with the butyrate producing bacteria *Roseburia*. Finally, *Akkermansia* that has been inversely associated with obesity, diabetes, cardiometabolic diseases and low-grade inflammation^21^, but also in response to cancer immunotherapy^22^, positively correlate with butyrate producing bacteria and TGFb2 and negatively correlate with inflammatory parameters (fecal neopterin, CD14+ monocytes and neutrophils).

### Clinical outcomes

Clinical outcomes and the effect of dysbiosis correction on clinical status (secondary outcome) are described for all patients having received the AFMT (*n* = 25). The median follow-up time for surviving patients was 29.9 (range 27.5–37.0) months. Among the treated patients, all but one, had responded to chemotherapy at V3 (96%), including 23 complete response and one partial response according to the 2017 ELN recommandation^23^. Seven patients relapsed at a median of 16.8 (range 3.9–27.8) months. The leukemia-free survival (LFS) rate was 88% at 6 months and 60% at 2 years (Supplementary Fig. 8). Eighteen patients received an alloHCT at a median of 5.0 (range 2.8–21.6) months after inclusion, including three patients after relapse and salvage chemotherapy. The cumulative incidence of grade II-IV acute GVHD at day 180 was 22% (*n* = 4), including three with gastrointestinal involvement (stage 1, *n* = 1; stage 2, *n* = 1 and stage 4, *n* = 1). In the entire cohort of patients, 8 died, the causes of death being relapse in two patients, GVHD in one, sepsis in two (one 113 days after AFMT in a patient with grade III acute GVHD after alloHCT and one 762 days after AFMT in a patient with AML relapse), CMV disease in one, multiorgan failure in one patient and thromboembolic event in one patient. The overall survival (OS) rate was 92% at 6 months and 72% at 2 years (Supplementary Fig. 8). Finally, in order to evaluate the impact of microbiota reconstitution at V3 on patients’ outcomes, we classify α-diversity indexes into tertiles as low, intermediate and high diversity and we compared OS and LFS between patients with low versus intermediate-high microbiota α-diversity. Two years OS and LFS were higher in patients with intermediate-high Shannon index at V3 (after AFMT), being 71% and 71% versus 62% and 38% respectively for patients with low Shannon index at V3 (*p* = 0.79 and *p* = 0.27, Supplementary Fig. 8).

## Discussion

The results of the current phase II study demonstrate that AFMT after IC in AML patients was feasible. Among the 28 patients that fulfilled the inclusion and exclusion criteria, only 3 patients refuse the procedure, and the remaining 25 patients received the AFMT. Of note, five patients could not receive the AFMT as planned after IC and hematopoietic recovery from aplasia for logistical reason or transient contraindication, but the AFMT was successfully administered after resolution of the issues. We also found the approach to be safe in immunocompromised patients, as they manifested minimal toxicity after AFMT administration. Only three patients experienced transient gastrointestinal symptoms (abdominal pain, *n* = 1 and diarrhea *n* = 2) within few days after AFMT. Most importantly, none of the patients developed SAE and, in particular, infectious complications within the month after AFMT. Only one patient experienced asymptomatic feces colonization by multidrug resistant *E. coli* at V3, 22 days after the second AFMT, followed by an *E. coli* sepsis 71 days later. Nevertheless, this SAE occurred 3 months after AFMT, raising the question of its link to the administered treatment. Safety was further confirmed by the low level of inflammatory parameters after AFMT. The good safety profile is in accordance with previously published results of FMT in immunocompromised patients with hematologic malignancies^24–26^.

Disruption of the intestinal microbiota by intensive chemotherapy and antibiotics is well established in patients with hematologic malignancies^5,6,8^, and our results confirm these findings with a decrease of gene richness after IC. Nevertheless, we found that the AFMT intervention reestablished the microbiota after its disruption, as evidenced by an increase of α-diversity indexes back to baseline levels after AFMT. Furthermore, the BC similarity index showed the restoration of microbial communities, and while microbiota reconstruction was not identical to baseline based on principal coordinates analysis we observed a restoration of the *Clostridiales Lachnospiraceae* and *Ruminococcaceae* after AFMT close to that of the initial level. Meanwhile, proinflammatory bacteria, such as *Enterobacteriaceae* and *Enterococceae* which predominate after IC, went back to a low level after AFMT. Importantly, Spearman correlation analysis over times of immunological and microbiota parameters confirm that butyrate producing immunoregulatory bacteria correlate negatively with pro-infammatory parameters, while *Enterococcus* was associated with increased fecal neopterin and ferritin levels. Furthermore, gut microbiota composition was preserved after consolidation chemotherapy (V4). Importantly, *Lachnospiraceae* and *Ruminococcacea*e have been associated with reduced mortality after alloHCT^27,28^, while *Enterobacteriaceae* and *Enterococcus* were associated with a decreased OS^27–29^ and an increased GVHD-related mortality^29^. Therefore, our findings suggest that our current strategy of AFMT and gut microbiota restoration after IC allow AML patients to get to alloHCT with a protective microbiota. In fact, 18 patients received an alloHCT and only three developed gastrointestinal GVHD, raising the question of a possible long-term protective effect of our approach. The cumulative incidence of clinically significant grade II-IV aGVHD was 22%, and microbiota α-diversity after AFMT has no impact on the cumulative incidence of aGVHD. Our results compare favorably with an incidence of 34.8% we reported previously^30^.

Another important finding was the significant reduction of the median number of reads mapped against antibiotic resistance genes observed at V3 after AFMT. We and others had reported that FMT is an effective strategy for MDRB eradication in patients with hematologic malignancies^24,31^. This is particularly important given that bloodstream infections from MDRB are associated with a poor outcome and an increased mortality^32^. Eradication of antibiotic resistance genes after AFMT may have partially contributed to the good outcomes reported in our study with a 2-year OS of 72%. Importantly, OS compared favorably with previously published studies. Therefore, lower two-years OS ranging from 41.9% to 60% have been reported in randomized phase 3 studies with a similar induction chemotherapy (cytosine arabinoside + anthracycline)^33,34^. Furthermore, although not statistically significant, two-years OS and LFS were higher in patients with intermediate-high Shannon index, further suggestion a long-term protective effect of our approach on OS and AML relapse.

One of the main limits of our study was the high incidence of screening failure, owing to the exclusion of a significant number of patients during screening prior to production of the AFMT inocula. This was due to strict, rigorous guidelines regarding microbiological testing and amount of feces required for the IMP production being followed. Nevertheless, these guidelines are indispensable to guarantee product safety. Use of third party donors as it has been done after alloHCT^26^ or of readily available pooled microbiota biotherapeutic, as it is currently investigated in GVHD^35^, may be a promising strategy to overcome this limitation. Also, one could object that gut microbiota composition will spontaneously restore back to normal without AFMT and the absence of a control group treated with placebo FMT prevent us from drawing any definitive conclusions on our findings. Additional immunological and microbiota analysis during follow-up, in particular at 6 months or before/after alloHCT would also have been useful to evaluate the long-term effect of our approach.

Finally, AFMT may not be appropriate in patients with previous antibiotic therapies or other treatments altering the composition of the gut microbiota. Therefore, the use of allogenic FMT may be more effective, and has already been shown to be feasible, safe, and associated with the expansion of recipient microbiome diversity after alloHCT^26^. Furthermore, the development of allogenic FMT from multiple donors in order to increase the amount of different bacterial species and, thus, enhance the global microbial richness of the treatment is of interest. This approach is currently being evaluated with FMT capsules for colon delivery in order to ease the administration of the product in a similar setting (Clinicaltrials.gov identifier: NCT04150393). Further clinical investigations will be required to evaluate the beneficial impact of this strategy implemented after each cycle of chemotherapy and HCT conditioning.

Overall, AFMT appears to be safe and effective for gut microbiota restoration in patients receiving IC and antibiotic for AML. Besides this particular setting, providing patients with a drug that may modulate inflammation and better potentiate other anti-cancer treatments is of considerable interest.

## Methods

### Study design

ODYSSEE was a phase I/II single-arm, multicentre, prospective, interventional trial in hospitalized patients with AML/ high-risk myelodysplastic syndrome to assess the feasibility and tolerance of AFMT in the setting of IC and antibiotic therapy given during aplasia. All patients were pre-evaluated within clinical practice before the official start of enrollment. All enrolled subjects gave their written informed consent to participate in the study. The study was approved by the ethics committee “Comité de protection des personnes Ile de France V” on April 5th, 2016, was conducted in full compliance with ICMJE guidelines and was registered in ClinicalTrials.gov (Identifier: NCT02928523). While study was submitted for registration on May 26, 2016, before the first patient inclusion on June 2016, there was a delay in the approval during the registration process while some innovative aspects were clarified and the study was finally registered on October 10, 2016. The full study protocol is available as Supplementary Note 1 in the Supplementary Information file.

### Study population and baseline assessment

**Fig. 7:**
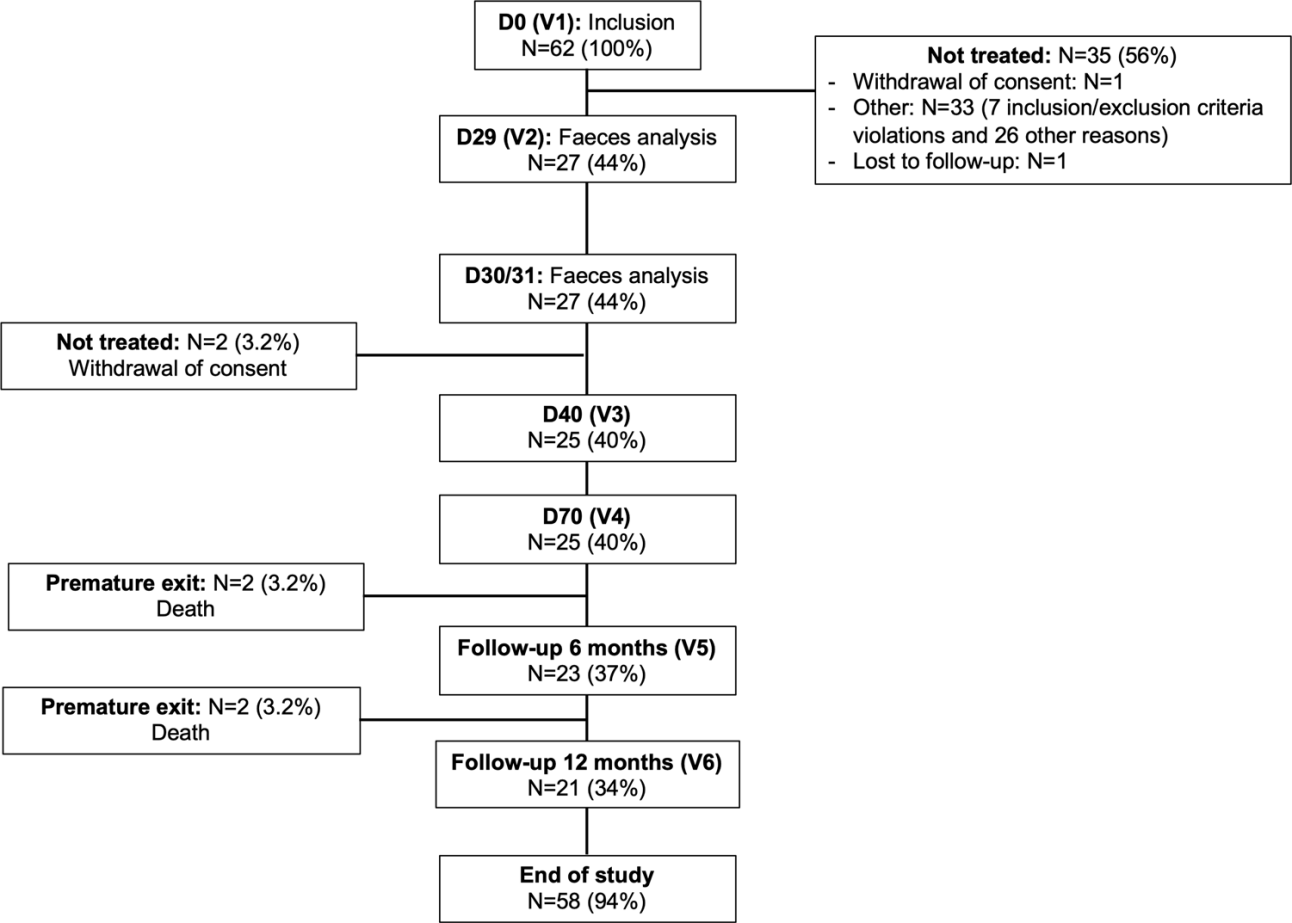
CONSORT diagram. AFMT is for autologous fecal microbiota transfer; D, day; N, number of patients, V1 to V6, visit 1 to visit 6.

A total of 62 patients between 24 and 69 years of age with de novo diagnosis of AML were screened from 7 French medical centers. Twelve patients were screened before trial was registered on October 10, 2016, of whom only two were treated before that date. The first patient was enrolled June 20, 2016 and the last patient was enrolled June 14, 2017. Patients with acute promyelocytic leukemia, and/or suffering from other severe diseases including digestive disorders (inflammatory bowel disease, severe colitis), or who received antibiotic therapy until 4 days before study inclusion were excluded from this trial (see Supplementary Table 7 for complete list of criteria). Microbiological safety was assessed in the feces collected at inclusion (Visit V1, Day D0) which were used for inoculum manufacturing. Detection of MDRB, bacterial pathogens, *Clostridium difficile*, parasites, noroviruses and/or rotaviruses (See Supplementary Table 8 for complete screening tests performed) led to the exclusion of patients. A total of 28 patients met all the inclusion/exclusion criteria (see Fig. 7 for Patient disposition and Supplementary Table 9 for screening failure detailed reasons), but three withdrew consent before the AFMT procedure. The treatment cohort comprised 25 patients.

### Study treatment

After hematopoietic recovery from aplasia, feces and blood were collected within 2 days before antibiotic discontinuation for bacteriological, metagenomics, biochemical, and immunological analyses (Visit V2). The AFMT was planned at the end of IC, after aplasia completion (ANC > 0.5 G/L) and 24 h after antibiotics discontinuation. AFMT was performed irrespective of platelet level, nevertheless platelet transfusion to increase platelet count before AFMT procedure was allowed based on the physician decision. In addition, patients with the following condition: persistent mucositis, colitis, or hemorrhoids were not eligible for AFMT according to exclusion criteria (Supplementary Table 7). Colonic preparation with laxative was administered before the first procedure. Patients received 2 inocula of 150 mL one day apart, each containing 30 g of feces using a rectal probe. Patients were placed in a lying position on their left side, with pelvis and legs elevated. After administration, the patients were asked to remain in this position for 20 min, and afterward turn on their belly, on their back and on their right side, maintaining each position for 15 min, allowing the product to flow into the colon and enhance microbiota colonization. Patient’s vital signs (blood pressure, pulse, temperature, respiratory rate) were monitored during all the time of the transplantation process, until the expulsion of the remaining transplant but at least 2 h after AFMT.

Among the 25 treated patients, a major protocol deviation was reported in five: four patients could only receive AFMT after the first consolidation chemotherapy due to AFMT contraindication in two patients (anal fistula (*n* = 1) and colitis (*n* = 1)) and for logistical organization in two others. For one patient, the period between V2 and AFMT was 35 days, due to an infectious event requiring prolongation of antibiotics, with a potential impact on the primary endpoint. Twenty patients were thus considered as per protocol. All figures and tables will present the data obtained from these patients, unless specified.

### Follow-up

Feces and blood were collected for the same analyses as before, prior to the initiation of consolidation chemotherapy (Visit V3), and at the end of the hospitalization (Visit V4). The quality of life of patients was assessed at each visit (V1 to V4), using an EQ-5D-5L questionnaire (evaluating the mobility, self-care, usual activities, pain/discomfort, anxiety, and depression). Finally, after 6 months (Visit V5) and 1 year (Visit V6), clinical information and safety assessment were recorded (see Fig. 1 for study flow chart). In addition, centers were contacted in September 2019 to collect long-term follow-up data regarding survival or death, including date of relapse, date and causes of death, undertaking or not and date of alloHCT and the occurrence or not of acute and chronic graft versus host disease (GvHD) after alloHCT.

### Production of AFMT inocula

Feces were collected at the time of patient’s admission, before the beginning of IC. The material was sent in a closed device at controlled ambient temperature (15-25 °C) to the manufacturing facility and processed following Good Manufacturing Practices. Feces were processed within 72 h with a patented cryoprotective diluent into a proprietary device (MaaT Pharma reference: 5M001), filtered, conditioned, and stored frozen at −80 °C until transplantation^36^.

### Quality control of AFMT inocula

Bacterial viability of the IMP was assessed by flow cytometry using the kit LIVE/DEAD® BacLight™ Bacterial Viability Kit from ThermoFisher Scientific^36^. This kit allows the distinction between live and dead bacteria based on the integrity of their membranes, through two fluorophores, SYTO9^®^ and propidium iodide (PI). SYTO9^®^ enters in all cells, undamaged or not, binds to the DNA (deoxyribonucleic acid) molecule and emits at 540 nm (green) after excitation at 470 nm (blue laser). PI also targets DNA molecules but only penetrates cells with damaged membranes (emission at 635 nm (red) after excitation at 470 nm.

Suspension samples were thawed at room temperature and immediately stained with a mix of the two fluorophores. Samples were then incubated for 15 min at room temperature protected from light, and next analyzed by flow cytometry. A control sample was processed in parallel to achieve a 0% viability and validate the test.

Bacteria, parasites, and viruses were screened in the fecal samples collected during the first visit to verify their safety for AFMT use. Detection of *C. difficile* and MDRB [multidrug resistant bacteria: methicillin resistant *Staphylococcus aureus*, vancomycin- and glycopeptide-resistant Enterococci, extended-spectrum beta-lactamase (ESBL) producing bacteria and carbapenemase-producing bacteria] was performed using PCR (polymerase chain reaction) and culture on specific isolation media, respectively. Parasites were detected using PCR (Microsporidia, *Dientamoeba fragilis*) or microscopy (*Strongyloides stercoralis*, *Cyclospora* sp., *Isospora* sp., *Entamoeba histolytica*, *Giardia intestinalis*, *Cryptosporidium* sp., *Blastocystis hominis*) after feces concentration. Noroviruses and Rotaviruses were identified by PCR and immunochromatography, respectively, and pathogenic bacteria were detected using PCR (*Campylobacter*) and culture (*Listeria* sp., *Vibrio* sp., *Yersinia* sp.) (See Supplementary Table 4).

Rigorous microbiological testing performed on the fresh stools according to the French health agency (ANSM) guideline^37^ and viability assessment by flow cytometry on the IMP allowed the release of the product after a quarantine period (Supplementary Tables 9, 10).

### Biochemical and immunological analyses

Biochemical and immunological analyses were performed on the different blood and feces samples collected during the first three visits. Neopterin and secretory IgA (sIgA) were measured from feces supernatants using the Neopterin ELISA (enzyme linked immunosorbent assay; IBL International) and IgA Secretory Human ELISA sa (EUROBIO) kits, respectively. Neopterin was also measured from plasma using the same Neopterin ELISA (IBL International). Total Antioxidant Status was measured from plasma using the Hitachi 912 kit (RANDOX Laboratories), and C-Reactive Protein levels were measured from plasma/serum samples in the different medical centers according to their own internal procedures.

### DNA isolation and metagenomic sequencing

Genomic DNA was extracted from the fecal samples collected during the first four visits using the Qiagen QIAamp Fast DNA stool mini kit and following a beat-beating procedure. A sequencing library was constructed for each DNA sample using the TruSeq Nano DNA Library Prep kit (Illumina) according to the manufacturer’s instructions. Libraries were then sequenced in 2 paired-end (2 × 125 bp) HiSeq2500 v4 (Illumina) runs. Raw sequencing data was downsized to 40 million reads/sample when possible.

### Bioinformatics analyses

Bioinformatics analyses were performed on Gut Print^®^ platform with the in-house MgRunner v1.1.2 pipeline. In brief, after quality filtering using Trimmomatic v0.36^38^, host sequence decontamination was performed using Bowtie2 v2.3.0^39^. Between 936,060 and 37,212,124 pairs of reads (mean: 34,811,750 pairs of reads) were obtained from the different samples. To ensure comparability, all samples were rarefied to the same sequencing depth i.e. 1,500,000 paired-end sequences per sample. Taxonomic profiling was then performed with Kraken v.0.10.5-beta^40^ and the RefSeq genomic database (release 84, 2017, http://www.ncbi.nlm.nih.gov/refseq/). The measure of α- and β-diversity indexes was performed in R Statistical Software (R Core Team 2015, version 3.4.4, http://www.R-project.org) using vegan v2.4-2 and phyloseq 1.19.1 packages. Gene-based analyses were performed using the Integrated Gene Catalogue (IGC)^41^. Finally, antibiotic resistance analyses were performed through gene mapping with Bowtie 2 v2.3.0 on MEGARes v1.0.1 (https://megares.meglab.org/) database.

### Clinical outcomes

The co-primary outcomes of the study were to evaluate the efficacy of AFMT in dysbiosis correction and MDRB eradication. The main secondary outcomes were (a) to define a dysbiosis biosignature using combination of biological parameters, (b) to evaluate the effect of dysbiosis correction on patient clinical status, (c) to assess the short and mid-term safety of AFMT in this immunocompromised population and (d) to evaluate the feasibility of the AFMT procedure and acceptability by the patient. Secondary outcomes b, c, and d only appear in the study protocol and do not appear on the registration site (clinicaltrial.gov). An individual patient Meta-analysis for a controlled result of efficacy was planned as an exploratory objective and will be conducted in a separate study.

### Sample size calculation and statistical analyses

By assuming repeated measurement between Simpson index post AFMT (Sf) and the two previous Simpson index values (Sb before antibiotherapy and chemotherapy and St post treatment) we assume that the correlation *R* (Sd, Sf) is at least *R* = 0.5. Without an accurate estimate of the standard deviation of Sd and Sf, we consider in this first-in-man trial that a change observed as at least half of its standard deviation should be considered as clinically meaningful as defined by Cohen’s rules. In these conditions, a sample size of at least 30 patients should provide a power of 0.875 to detect a difference of a clinically relevant difference Δ = | Sf−Sd | , at a one- tailed significance level of 0.05. Nevertheless, in order to limit the number of cases, given the expected recruitment difficulties, sample size was evaluated according to beta values and effect size. When the effect size is at least 0.6 and with a beta risk of at least 0.20, 20 patients should constitute an acceptable size. Therefore, a sample of 20 patients constitutes an acceptable sample size for this pilot study.

A planned interim analysis was performed on the first 9 patients fulfilling inclusion and exclusion criteria^42^. The correlation R between St and Sf was 0.83 (Spearman correlation), higher than the requested (0.5). Furthermore, measurement of clinical relevance was based on the standardized mean difference or cohen effect size. The Standard Mean Deviation was 1.063 [(mean Sf -mean St)/pooled standard deviation]. Therefore, based on this large effect size it was not necessary to increase the sample size. Based on the result of the interim analysis performed on the first 9 patients, it was not necessary to adapt the sample size and futility analysis was not performed.

For most visit and parameter, the normality distribution, including after a log transformation, was rejected by Shapiro–Wilk test (Supplementary Table 11). In two cases (Inverse Simpson and number of genes after log transformation) data follow a Normal distribution, nevertheless, homoscedasticity is not respected. Hence, a non-parametric approach has been followed with two-sided signed-rank Wilcoxon paired test using GraphPad Prism 7.

### Reporting summary

Further information on experimental design is available in the Nature Research Reporting Summary linked to this paper.

## Supplementary information

Supplementary Information

Reporting summary

## Data Availability

Sequence data that support the findings have been deposited in the NCBI SRA database under the BioProject accession PRJNA715586, and available at http://www.ncbi.nlm.nih.gov/bioproject/715586). All the other data supporting the findings of this study are available within the article and its supplementary information files and from the corresponding author upon reasonable request. Source data are provided with this paper.

## Source data

Source Data
