# Supplementary material for: Gut microbiota diversity after autologous fecal microbiota transfer in acute myeloid leukemia patients
Source: Nat Commun. 2021 May 25;12:3084. doi: 10.1038/s41467-021-23376-6 (PMC8149453; doi:10.1038/s41467-021-23376-6)
Supplement: Supplementary file 1 — Supplementary Information [file 41467_2021_23376_MOESM1_ESM.pdf]

Restoration of gut microbiota diversity with autologous fecal microbiota transfer in acute myeloid leukemia patients

Florent Malard, Anne Vekhoff, Simona Lapusan, Francoise Isnard, Evelyne D'incan-Corda, Jérôme Rey, Colombe Saillard, Xavier Thomas, Sophie Ducastelle-Lepretre, Etienne Paubelle, Marie-Virginie Larcher, Clément Rocher, Christian Recher, Suzanne Tavitian[4], Sarah Bertoli, Anne-Sophie Michallet, Lila Gilis, Pierre Peterlin, Patrice Chevallier, Stéphanie Nguyen, Emilie Plantamura, Lilia Boucinha, Cyrielle Gasc, Mauricette Michallet, Joel Dore, Ollivier Legrand, Mohamad Mohty

Supplementary information

Supplementary Tables

Supplementary Table 1. Baseline demographics and clinical characteristics of screen failed patients (n=37).

		#	%
**Gender**	Male	17	46
	Female	20	54
**Age at inclusion (years)**	Median (Range)	58 [29-69]	
**Risk category**	Favourable	4	12
	Intermediate	24	70
	Unfavourable	6	18
	Missing data	3	-
**Body mass index at inclusion**	Median (Range)*	24.4 [19.6-44.6]	-<br>-<br>-<br>-

* Missing data n=1

Supplementary Table 2. Descriptive statistics of quality of life questionnaire for treated patients (n=25).

Parameters	Statistics	V1	V2	V3	V4
**Mobility**	Missing	3	4	6	7
	I have no problems in walking about	18 (82%)	18 (86%)	16 (84%)	14 (78%)
	I have slight problems in walking about	3 (14%)	3 (14 %)	3 (16%)	3 (17%)
	I have moderate problems in walking about	1 (4%)	0 (0%)	0 (0%)	1 (5%)
	I have severe problems in walking about	0 (0%)	0 (0%)	0 (0%)	0 (0%)
	I am unable to walk about	0 (0%)	0 (0%)	0 (0%)	0 (0%)
**Self-care**	Missing	3	4	5	7
	I have no problem washing or dressing myself	21 (95%)	19 (90%)	19 (95%)	16 (89%)
	I have slight problems washing or dressing myself	1 (5%)	2 (10%)	1 (5%)	2 (11%)
	I have moderate problems washing or dressing myself	0 (0%)	0 (0%)	0 (0%)	0 (0%)
	I have severe problems washing or dressing myself	0 (0%)	0 (0%)	0 (0%)	0 (0%)
	I am unable wash or dress myself	0 (0%)	0 (0%)	0 (0%)	0 (0%)
**Usual activities**	Missing	5	4	5	7
	I have no problems doing my usual activities	12 (60%)	13 (62%)	15 (75%)	11 (61 %)
	I have slight problems doing my usual activities	4 (20%)	6 (29%)	4 (20%)	5 (28%)
	I have moderate problems doing my usual activities	2 (10%)	2 (9%)	1 (5%)	2 (11%)
	I have severe problems doing my usual activities	1 (5%)	0 (0%)	0 (0%)	0 (0%)
	I am unable do my usual activities	1 (5%)	0 (0%)	0 (0%)	0 (0%)
**Pain / Disconfort**	Missing	3	4	5	7
	I have no pain or discomfort	10 (46%)	17 (81%)	16 (80%)	13 (72%)
	I have slight pain or discomfort	8 (36 %)	4 (19%)	4 (20%)	4 (22%)
	I have moderate pain or discomfort	4 (18%)	0 (0%)	0 (0%)	1 (6%)
	I have severe pain or discomfort	0 (0%)	0 (0%)	0 (0%)	0 (0%)
	I have extreme pain or discomfort	0 (0%)	0 (0%)	0 (0%)	0 (0%)
**Anxiety and depression**	Missing	3	4	5	8
	I am not anxious or depressed	11 (50%)	14 (67%)	15 (75%)	8 (47%)
	I am slight anxious or depressed	5 (23%)	4 (19%)	3 (15%)	6 (35%)
	I am moderately anxious or depressed	4 (18%)	3 (14%)	2 (10%)	3 (18%)
	I am severely anxious or depressed	2 (9%)	0 (0%)	0 (0%)	0 (0%)
	I am extremely anxious or depressed	0 (0%)	0 (0%)	0 (0%)	0 (0%)
**Your health today**	N (Missing)	20 (5)	21 (4)	20 (5)	17 (8)
	Mean (SD)	61.05 (26.05)	76.24 (16.44)	78.75 (16.61)	71.47 (15.49)
	(Min;Max)	(9;95)	(45;100)	(50;95)	(40;95)
	Median (Q1;Q3)	62.5 (47.5;80)	80 (70;90)	85 (65;95)	70 (65;80)

Supplementary Table 3. Monitoring of vital signs of treated patients (n=25) during AFMT procedure.

Parameters	Statistics	AFMT n°1	AFMT n°2
**Temperature min (C°)**	N (Missing)	24 (1)	24 (1)
	Mean (SD)	36.68 (0.47)	36.54 (0.39)
	(Min;Max)	(36.00;37.80)	(35.70;37.30)
	Median (Q1;Q3)	36.55 (36.35;37.05)	36.50 (36.30;36.80)
**Temperature max (C°)**	N (Missing)	22 (3)	21 (4)
	Mean (SD)	37.14 (0.45)	37.01 (0.31)
	(Min;Max)	(36.40;38.10)	(36.50;37.70)
	Median (Q1;Q3)	37.10 (36.90;37.40)	37.10 (36.80;37.20)
**Heart rate (BPM)**	N (Missing)	24 (1)	25 (0)
	Mean (SD)	73.21 (8.70)	74.36 (6.95)
	(Min;Max)	(56.00;88.00)	(61.00;88.00)
	Median (Q1;Q3)	73.50 (69.00;79.50)	75.00 (71.00;77.00)
**Blood pressure min : Systolic**	N (Missing)	25 (0)	25 (0)
	Mean (SD)	118.28 (16.20)	120.16 (13.64)
	(Min;Max)	(90.00;150.00)	(90.00;160.00)
	Median (Q1;Q3)	118.00 (110.00;130.00)	120.00 (111.00;125.00)
**Blood pressure min : Diastolic**	N (Missing)	25 (0)	25 (0)
	Mean (SD)	69.44 (13.15)	67.84 (11.35)
	(Min;Max)	(55.00;114.00)	(49.00;90.00)
	Median (Q1;Q3)	69.00 (60.00;71.00)	66.00 (60.00;75.00)
**Blood pressure max : Systolic**	N (Missing)	22 (3)	22 (3)
	Mean (SD)	132.95 (16.99)	136.73 (16.59)
	(Min;Max)	(100.00;179.00)	(110.00;180.00)
	Median (Q1;Q3)	131.00 (120.00;140.00)	136.50 (128.00;147.00)
**Blood pressure max : Diastolic**	N (Missing)	22 (3)	22 (3)
	Mean (SD)	73.18 (12.00)	79.32 (11.34)
	(Min;Max)	(60.00;92.00)	(60.00;100.00)
	Median (Q1;Q3)	73.50 (60.00;83.00)	80.00 (70.00;90.00)

Supplementary Table 4. Adverse events 24-hours after AFMT by system organ class in treated patients (n=25).

SOC	PT	# (%)
Gastrointestinal disorders	Abdominal pain	1 (20%)
	Diarrhea	2 (40%)
General disorders and administration site conditions	Pyrexia	1 (20%)
Investigations	Weight increased	1 (20%)

Supplementary Table 5. Adverse events after the first 24-hours after AFTM by system organ class in treated patients (n=25).

SOC	# (%)
Blood and lymphatic system disorders	58 (14%)
Cardiac disorders	2 (0%)
Congenital, familial and genetic disorders	8 (2%)
Eye disorders	1 (0%)
Gastrointestinal disorders	78 (18%)
General disorders and administration site conditions	39 (9%)
Hepatobiliary disorders	8 (9%)
Immune system disorders	6 (1%)
Infections and infestations	88 (21%)
Injury, poisoning and procedural complications	13 (3%)
Investigations	20 (5%)
Metabolism and nutrition disorders	14 (3%)
Musculoskeletal and connective tissue disorders	12 (3%)
Nervous system disorders	18 (4%)
Psychiatric disorders	5 (1%)
Renal and urinary disorders	2 (0%)
Reproductive system and breast disorders	1 (0%)
Respiratory, thoracic and mediastinal disorders	17 (4%)
Skin and subcutaneous tissue disorders	15 (4%)
Surgical and medical procedures	1 (0%)
Vascular disorders	9 (2%)

Supplementary Table 6. Severe adverse events after the first 24-hours after AFMT by system organ class in treated patients (n=25).

SOC	# (%)
Blood and lymphatic system disorders	1 (3%)
Gastrointestinal disorders	1 (3%)
General disorders and administration site conditions	3 (10%)
Immune system disorders	3 (10%)
Infections and infestations	13 (43%)
Injury, poisoning and procedural complications	2 (6%)
Investigations	1 (3%)
Metabolism and nutrition disorders	1 (3%)
Nervous system disorders	1 (3%)
Respiratory, thoracic and mediastinal disorders	2 (6%)
Skin and subcutaneous tissue disorders	1 (3%)
Vascular disorders	1 (3%)

Supplementary Table 7. Selection of patients for the study.

**Inclusion criteria**	- Patients ≥ 18 and ≤ 75 years old with *de novo* diagnosis of AML or HR MDS for whom intensive induction chemotherapy was anticipated within 10 days after admission;<br>- Patients willing to donate stool samples and to follow protocol recommendations;<br>- Signature of informed and written consent.
**Exclusion criteria**	- Acute promyelocytic leukemia;<br>- Known allergy or intolerance to trehalose or maltodextrin;<br>- Pregnancy (positive urinary or blood test in female of childbearing potential);<br>- Severe disease with a life expectancy < 3 months;<br>- Other ongoing interventional protocol that might interfere with the study;<br>- Non eligibility for collection of autologous stools upon admission:<br>- Patients refusing to consent;<br>- Antibiotic therapy at the time of study inclusion ≥ 4 days;<br>- Concomitant or previous diagnosis of a significant inflammatory bowel disease (ulcerative colitis, Crohn's disease) or other progressive digestive disease requiring treatment or further medical exploration;<br>- Presence of severe colitis of any etiology at the time of admission or severe digestive disorders (acute or chronic diarrhea) within 3 months preceding inclusion;<br>- Presence of blood in feces collected at the time of inclusion;<br>- Patient subjected to a recent colonoscopy (within 3 months preceding inclusion);<br>- Detection of MDRB, pathogenic bacteria, parasites, norovirus and/or rotavirus during screening of autologous stool collected immediately after the inclusion visit;<br>- Non eligibility for inoculum transplantation: persistent mucositis, colitis, or hemorrhoids, presence of blood in more than 1 out of 3 feces samples the week preceding the transplantation;<br>- Non feasibility of inoculum procedure: patient refusal, technical or biological mismatch of the inoculum;<br>- Absence of effective contraceptive method for female of childbearing potential;<br>- Lactation;<br>- Inability to give an informed consent.

Supplementary Table 8. List of screening tests performed in feces.

**Biochemistry (feces)**	Calprotectin		ELISA assay
	Zonulin		ELISA assay
	Neopterin		ELISA assay
	IgA		ELISA assay
**Microbiology (feces)**	*C. difficile*		PCR
	Norovirus		PCR
	Rotavirus		Immunochromatography
	MDRB	MRSA	PCR
		VRE and GRE	Culture (2 specific media)
		ESBLs	Culture (2 specific media)
		Carbapenemases	Culture (2 specific media)
	Pathogenic bacteria	*Campylobacter sp*	PCR
		Listeria sp	Culture (ALOA)
		Salmonella sp	PCR
		Shigella sp	PCR
		Vibrio sp	Culture (after enrichment)
		Yersinia sp	Culture (Cefsulodin-Irgasan-Novobiocine)
	Parasites	Strongyloides stercoralis, Cyclospora, Isospora, Entamoeba histolytica, Giardia intestinalis, Cryptosporidium, Microsporidies, Dientamoeba fragilis, Blastocystis hominis	Feces concentration – coproculture and PCR

IgA : A Immunoglobulin ; sp : species, PCR: Polymerase Chain Reaction; MDRB: Multi-Drug Resistant Bacteria; VRE and GRE: Vancomycin and Glycopeptide-resistant Enterococci; ESBLs: Extended Spectrum Beta Lactamase-producing bacteria; ALOA: Agar Listeria according to Ottaviani & Agosti

Supplementary Table 9. Reasons for screening failure (n=37).

**Related to IMP manufacturing n=22 (59%)**	Insufficient raw material; n=14 (38%)
	Logistic failure; n=4 (11%)
	Quality control for batch release not reached; n=4 (11%)
**Related to the patient n=15 (41%)**	FMT not performed due to patients' condition; n=5 (14%)
	Patient's refusal to collection of the inoculum procedure (consent withdraw); n=3 (8%)
	AML diagnosis not confirmed; n=1 (3%)
	MDRB or *C. difficile* carriage at diagnosis; n=6 (16%)

IMP: Investigational Medicinal Product; FMT: Fecal Microbiota Transfer; AML: Acute Myeloid Leukemia; MDRB: Multi-Drug Resistant Bacteria

Supplementary Table 10. IMP batch release specifications

Description		Specifications
Aspect – Visual method		Homogenous colored suspension, from yellow to brown
Bacterial viability		> 20%
Purity	Methicillin-resistant *Staphylococcus aureus*<br>Vancomycin and Glycopeptid-resistant Enterococci<br>Extended spectrum beta lactamase producing bacteria<br>Carbapenemase producing bacteria	Absence of multi-drug resistant bacteria
	Campylobacter sp<br>*Listeria* sp<br>*Salmonella* sp<br>*Shigella* sp<br>*Vibrio* sp<br>*Yersinia* sp<br>Toxinogenic *Clostridium difficile*	Absence of pathogenic bacteria
	Strongyloides stercoralis<br>Cryptosporidium<br>Cyclospora<br>Isospora<br>Entamoeba histolytica<br>Giardia intestinalis<br>Microsporidia<br>Bastocystis hominis<br>Dientamoeba fragilis	Absence of parasites
	Noroviruses (1 and 2)<br>Rotavirus	Absence of viruses

Supplementary Table 11. Evaluation of normal distribution of raw data and log-transformed data and of homoscedasticity of data with normal distribution.

		Shapiro-Wilk test		Bartlett test
		**Raw data**	**Log-transformed data**	
BrayCurtis	V1V2	W= 0.853<br>p-value= 0.019	W= 0.891<br>p-value=0.069	
	V1V3	W= 0.898<br>p-value=0.037	W= 0.895<br>p-value=0.033	
	V2V3	W= 0.813<br>p-value=0.005	W= 0.909<br>p-value=0.130	
	V3V4	W= 0.944<br>p-value=0.407	W= 0.901<br>p-value=0.084	
Shannon	V1	W= 0.872<br>p-value=0.013	W= 0.792<br>p-value=0.001	
	V2	W= 0.944<br>p-value=0.440	W= 0.728<br>p-value=0.001	
	V3	W= 0.972<br>p-value=0.789	W= 0.946<br>p-value=0.308	
	V4	W= 0.939<br>p-value=0.339	W= 0.906<br>p-value=0.100	
Inverse Simpson	V1	**W= 0.950**<br>**p-value=0.365**	W= 0.900<br>p-value=0.040	Raw data:<br>K-squared=10.706<br>df=3<br>p-value=0.013
	V2	**W= 0.889**<br>**p-value=0.065**	W= 0.969<br>p-value=0.841	
	V3	**W= 0.958**<br>**p-value=0.505**	W= 0.969<br>p-value=0.733	
	V4	**W= 0.921**<br>**p-value=0.176**	W= 0.892<br>p-value=0.060	
ABR	V1	W= 0.978<br>p-value=0.900	W= 0.947<br>p-value=0.328	
	V2	W= 0.937<br>p-value=0.254	W= 0.690<br>p-value=4.683×10^{-5}	
	V3	W= 0.899<br>p-value=0.039	W= 0.979<br>p-value=0.926	
	V4	W= 0.925<br>p-value=0.205	W= 0.980<br>p-value=0.964	

Fecal neopterin	V1	W= 0.645<br><br>p-value=2.916×10^{-5}	W= NaN (several values = 0)<br><br>p-value=NA	
	V2	W= 0.893<br><br>p-value=0.037	W= NaN (several values = 0)<br><br>p-value= NA	
	V3	W= 0.675<br><br>p-value=1.955×10^{-5}	W= NaN (several values = 0)<br><br>p-value= NA	
CRP	V1	W= 0.662<br><br>p-value=2.002×10^{-5}	W= NaN (several values = 0)<br><br>p-value= NA	
	V2	W= 0.907<br><br>p-value=0.089	W= NaN (several values = 0)<br><br>p-value= NA	
	V3	W= 0.520<br><br>p-value=3.113×10^{-6}	W= NaN (several values = 0)<br><br>p-value= NA	
IgA	V1	W= 0.780<br><br>p-value=0.001	W= 0.903<br><br>p-value=0.077	
	V2	W= 0.935<br><br>p-value=0.239	W= 0.741<br><br>p-value=0.001	
	V3	W= 0.884<br><br>p-value=0.021	W= 0.847<br><br>p-value=0.005	
Plasmatic neopterin	V1	W= 0.848<br><br>p-value=0.055	W= 0.977<br><br>p-value=0.946	
	V2	W= 0.843<br><br>p-value=0.048	W= 0.841<br><br>p-value=0.045	
	V3	W= 0.523<br><br>p-value=6.688×10^{-6}	W= 0.773<br><br>p-value=0.007	
Genes	V1	W= 0.952<br><br>p-value=0.395	**W= 0.911**<br><br>**p-value=0.066**	Log-transformed data:<br><br>K-squared=20.958<br><br>df=3<br><br>p-value=0.001
	V2	W= 0.759<br><br>p-value=0.001	**W= 0.924**<br><br>**p-value=0.194**	
	V3	W= 0.946<br><br>p-value=0.306	**W= 0.985**<br><br>**p-value=0.981**	
	V4	W= 0.935<br><br>p-value=0.293	**W= 0.893**<br><br>**p-value=0.063**	

TAS	V1	W= 0.949<br><br>p-value=0.471	W= 0.935<br><br>p-value=0.289	
	V2	W= 0.966<br><br>p-value=p=0.749	W= 0.986<br><br>p-value=0.993	
	V3	W= 0.582<br><br>p-value=7.065×10^{-6}	W= 0.728<br><br>p-value=0.001	

P value were determined by two-sided Shapiro-Wilk test, for normality and Bartlett's test for homoscedasticity, no adjustments were made for multiple comparisons.

Supplementary Figures

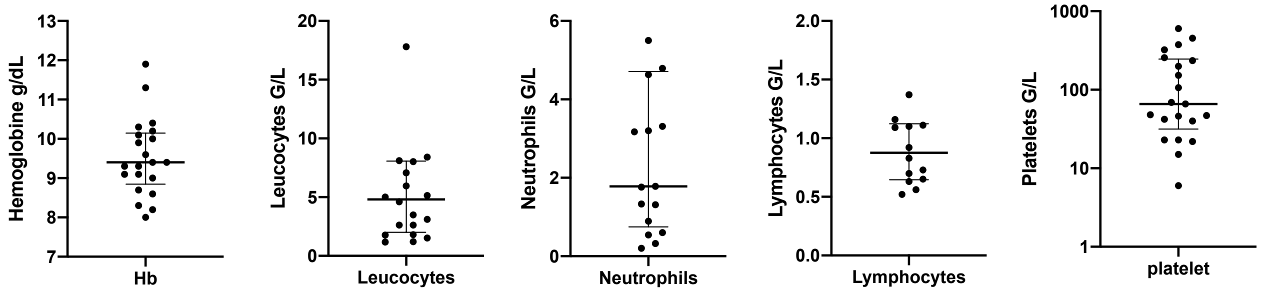

Supplementary Figure 1: Haemoglobin level, leucocytes count, absolute neutrophil count (ANC), lymphocytes count and platelets count at time of AFMT (n=25, error bars indicate median and interquartile range, source data are provided as a Source Data file).

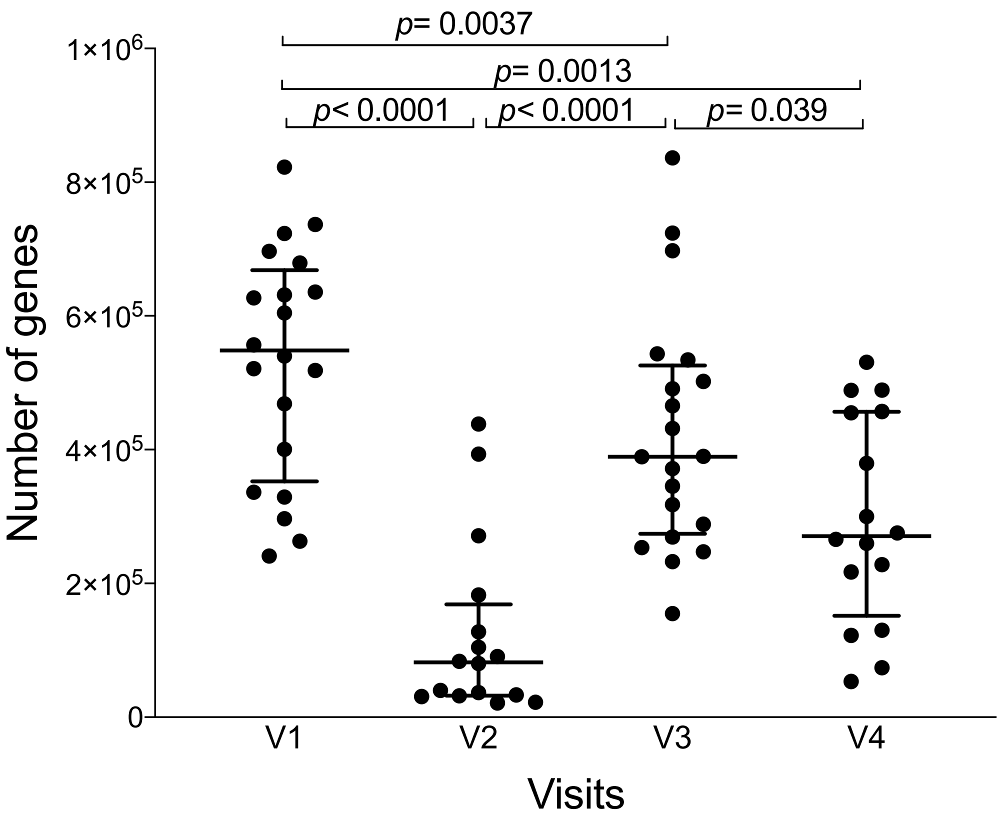

Supplementary Figure 2: Gene richness of the gut microbiota from visit 1 to visit 4 (V1 to V4) in *per protocol* patients (n=20, P value were determined by two-sided signed-rank Wilcoxon paired test, no adjustments were made for multiple comparisons, error bars indicate median and interquartile range, source data are provided as a Source Data file).

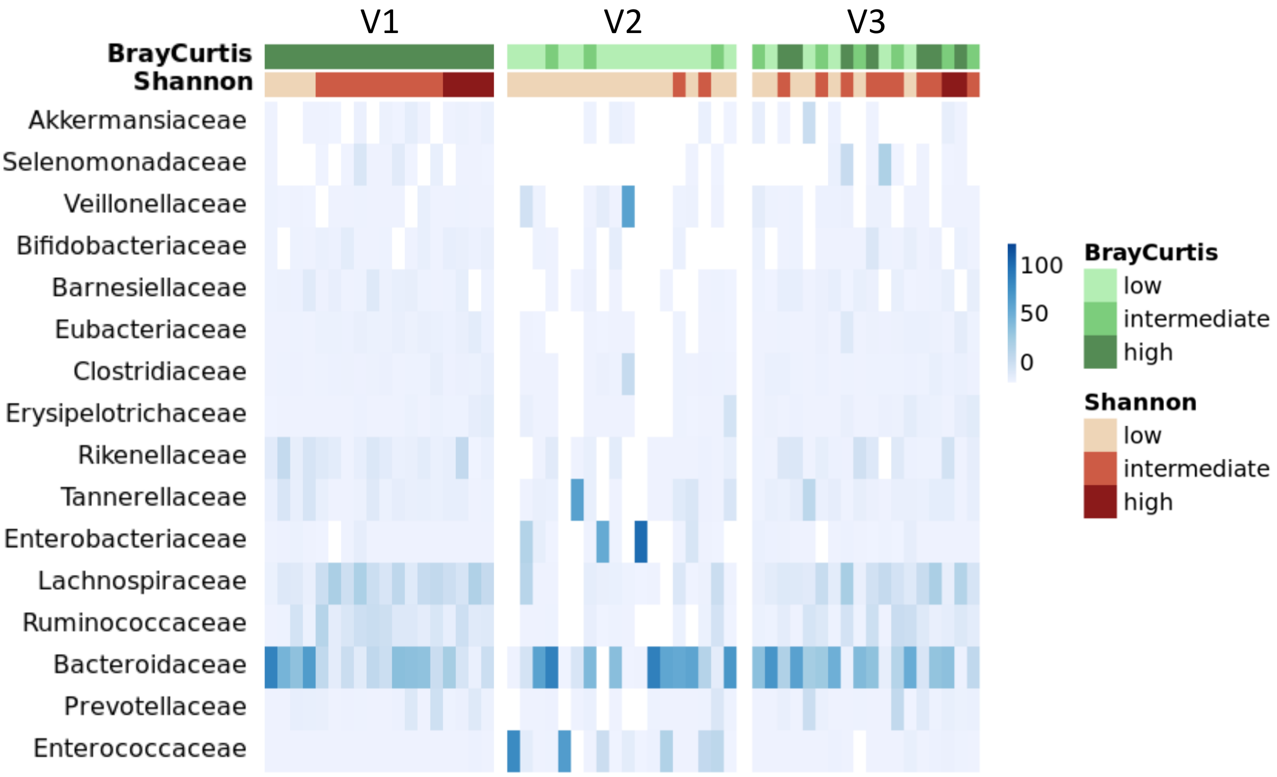

Supplementary Figure 3: Family profile heatmap.

The relative abundances of the families found in the different samples are represented in shades of blue. Each column corresponds to a patient and the order of the columns is respected between the different panels. Only patients for which a complete kinetics is available from visit 1 to visit 3 (V1 to V3) are shown (n=18). For each sample, the associated values of the Shannon index at the species level and the BrayCurtis similarity with respect to V1 at the species level are represented. For Shannon index, ranges were based on the Shannon value of the study population: low < first quartile, medium ≥ first quartile and < third quartile, high ≥ third quartile. For Bray-Curtis dissimilarity index: low < 0.4, medium ≥ 0.4 and < 0.6 and high ≥ 0.6. The choice of this mathematically determination was made before the results analysis.

V1

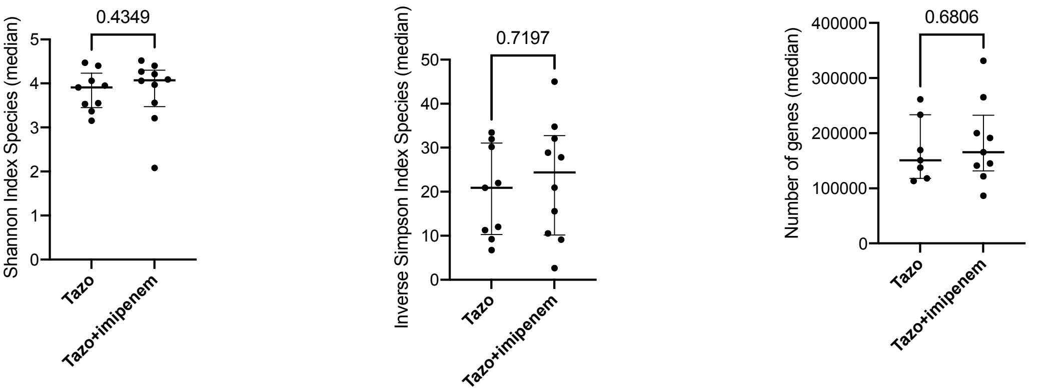
0.4349
Shannon Index Species (median)
Tazo
Tazo+imipenem
0.7197
Inverse Simpson Index Species (median)
0.6806
Number of genes (median)

V2

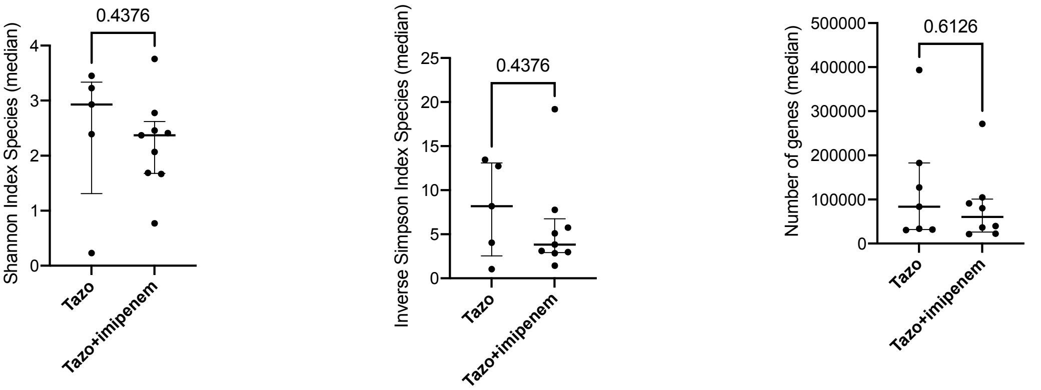
0.4376
Shannon Index Species (median)
Tazo
Tazo+imipenem
0.4376
Inverse Simpson Index Species (median)
0.6126
Number of genes (median)

V3

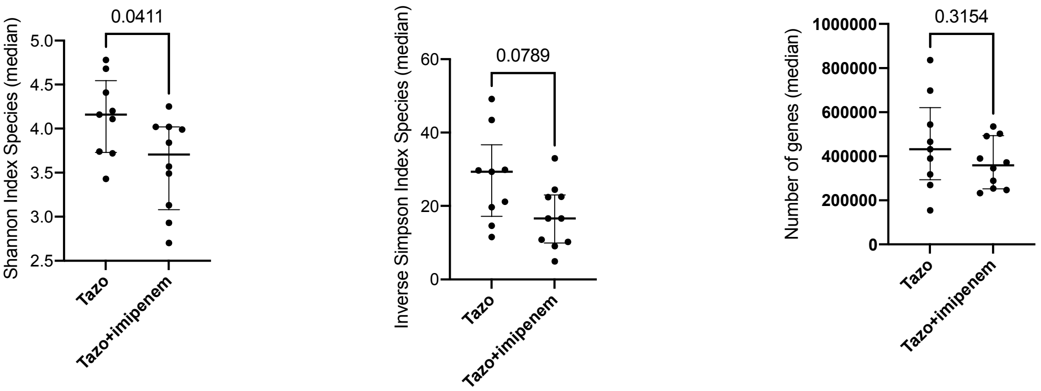
0.0411
Shannon Index Species (median)
Tazo
Tazo+imipenem
0.0789
Inverse Simpson Index Species (median)
0.3154
Number of genes (median)

V4

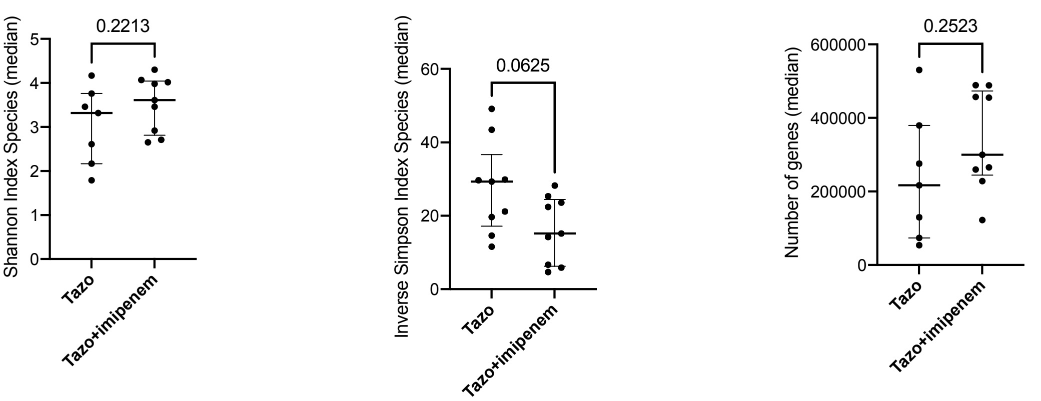
0.2213
Shannon Index Species (median)
Tazo
Tazo+imipenem
0.0625
Inverse Simpson Index Species (median)
0.2523
Number of genes (median)

Supplementary Figure 4: Alpha microbiota diversity from visit 1 to visit 4 (V1 to V4) according to the antibiotic administered during the induction chemotherapy. (n=19, P value were determined by two-sided Mann Whitney unpaired test, error bars indicate median and interquartile range, source data are provided as a Source Data file).

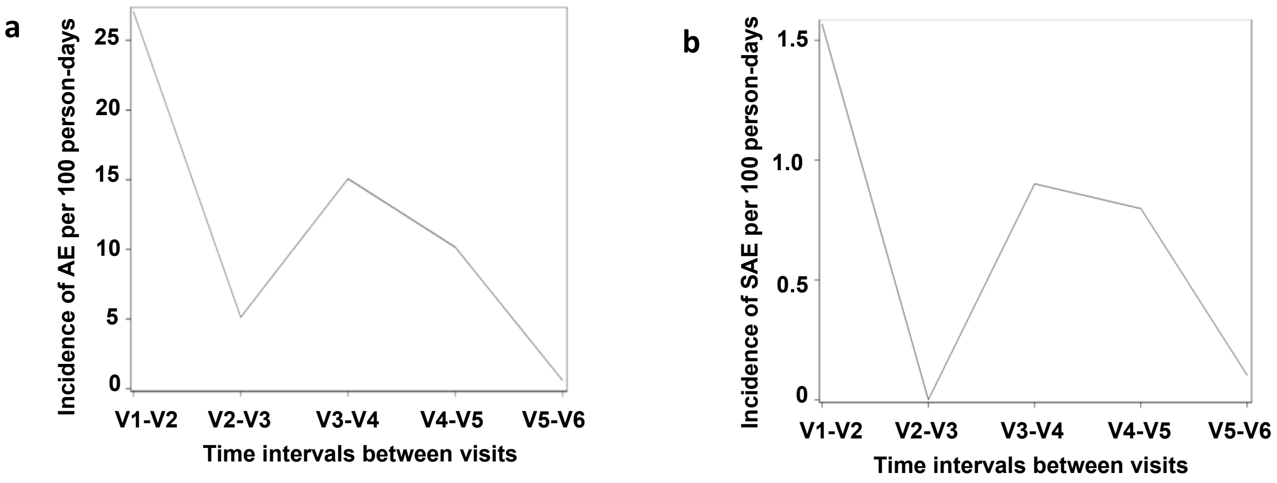

c

Interval	Person-days	# AE	# subject with >1 AE	IR AE	# SAE	# subject with >1 SAE	IR SAE
	Included in FAS N=25						
Before D0	.	30	14 (56.00%)	.	0	0 (0.00%)	.
D0-D29	957	259	25 (100.00%)	27.06	15	5 (20.00%)	1.57
D29-D40	546	28	10 (40.00%)	5.13	0	0 (0.00%)	0.00
D40-D70	1221	184	23 (92.00%)	15.07	11	3 (12.00%)	0.90
D70-6m	1881	191	20 (80.00%)	10.15	15	10 (40.00%)	0.80
6m-12m	3909	23	8 (32.00%)	0.59	4	4 (16.00%)	0.10

IR:Incidence rate for 100 person-days

Supplementary Figure 5: Incidence rate of AE and SAE by interval between visits for treated patients (n=25).

(a) AE (b) SAE (c) descriptive statistics od AE and SAE by interval between visits.

AE is for adverse event, SAE, serious adverse event, V1 to V6, visit 1 to visit 6.

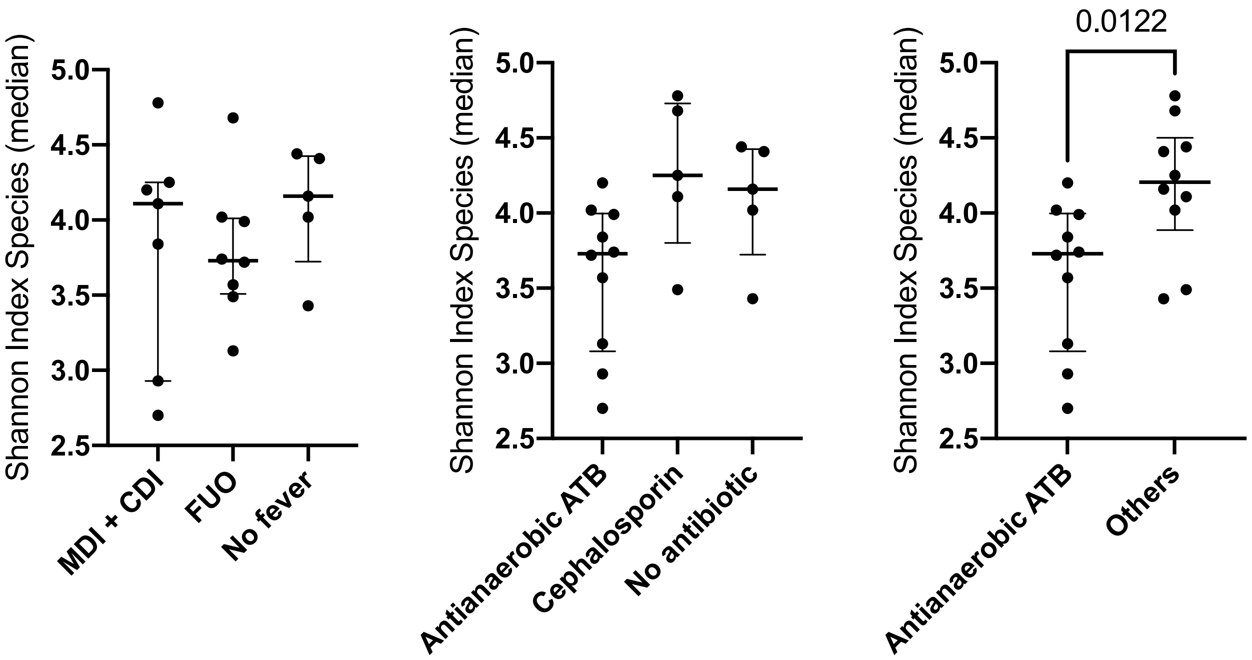

Supplementary Figure 6. Shannon index species at visit 3 (V3) according to infectious adverse events and use of antibiotic during consolidation chemotherapy. (n=20, P value was determined by two-sided Mann Whitney unpaired test, error bars indicate median and interquartile range).

ATB is for antibiotics; MDI is for microbiologically defined infection; CDI, clinically defined infection; FUO, fever of unknown origin. Source data are provided as a Source Data file.

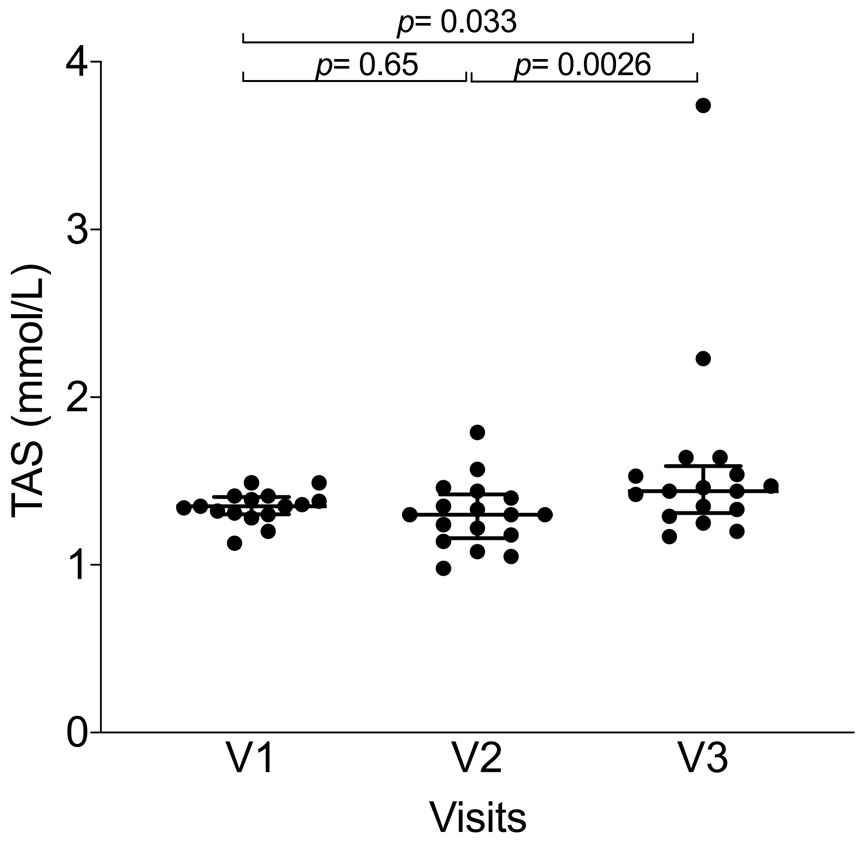

Supplementary Figure 7: Evolution of Total Antioxidant Status (TAS) from visit 1 to visit 3 (V1 to V3) in *per protocol* patients. (n=20, P value were determined by two-sided signed-rank Wilcoxon paired test, no adjustments were made for multiple comparisons, error bars indicate median and interquartile range, source data are provided as a Source Data file).

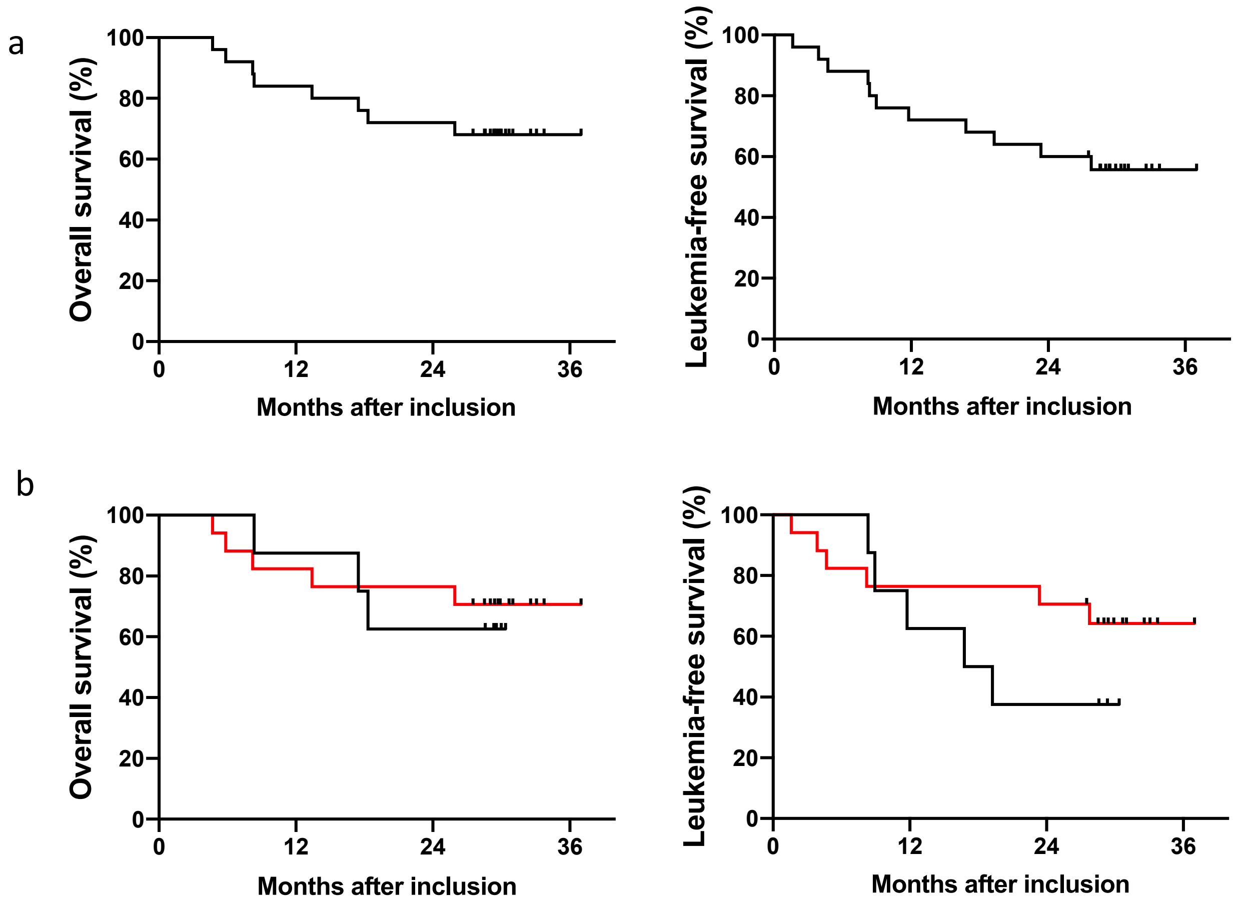

Supplementary Figure 8: Overall survival and leukemia-free survival.

a. Kaplan-Meyer estimate of overall survival and leukemia-free survival in the 25 treated patients. b. Kaplan-Meyer estimate of overall survival and leukemia-free survival according to gut microbiota α-diversity (black line: low α-diversity, red line: intermediate/high α-diversity, source data are provided as a Source Data file).

MaaT Pharma	Protocol	Protocol N°: MPOH02<br>EudraCT N° : 2015-004335-12<br>Version 10.0<br>Date : 20 November 2017
	**ODYSSEE STUDY**	

PreventiOn of DYSbioSis complications with autologous fecal microbiota transplantation in acutE myEloid leukemia patients undergoing intensive treatment: a feasibility and safety study
ODYSSEE STUDY

Principal Investigator:

Pr Ollivier LEGRAND
Hématologie Clinique et Thérapie Cellulaire
Hôpital Saint-Antoine
Assistance Publique - Hôpitaux de Paris
184, rue du faubourg Saint-Antoine – 75012 Paris
Tél: 01.49.28.26.20
Fax: 01.49.28.32.00
Email: ollivier.legrand@aphp.fr

Coordinating Investigator:

Pr Mohamad MOHTY
Hématologie Clinique et Thérapie Cellulaire
Hôpital Saint-Antoine
Assistance Publique - Hôpitaux de Paris
184, rue du faubourg Saint-Antoine – 75012 Paris
Tél: 01.49.28.26.20
Fax: 01.49.28.32.00
Email: mohamad.mohty@inserm.fr

Sponsor:

MaaT Pharma
317 avenue Jean Jaurès, 69007 LYON

All documentation of **MaaT Pharma** is PROPRIETARY information and may not be forwarded to third parties without prior written consent from **MaaT Pharma.** Reproductions, either in part or in whole, may not be published or copied in any manner, without the explicit written consent of **MaaT Pharma**

Version History

Version	Date	Author	Change description
2.0	24 Jun 2015	E. Plantamura	Initial version- Document submitted to the technical facilitation meeting with ANSM (2015/07/03)
3.0	04 Feb 2016	E. Plantamura	Changes made according to the ANSM's questions after pre-submission
4.0	04 May 2016	E. Plantamura	Changes made according to CPP and ANSM comments and after amendment dated 04 May 2016.
5.0	27 Jun 2016	E. Plantamura	Changes made according to ANSM comments during first amendment submission
6.0	10 Aug 2016	E. Plantamura	Changes related to amendment N°2
7.0	15 Dec 2016	E. Plantamura	Changes related to amendment N°3
8.0	26 Jan 2017	E. Plantamura	Changes made according to ANSM comments during third amendment submission
9.0	28 Feb 2017	E. Plantamura	Changes related to amendment N°4
10.0	20 Novembre 2017	E. Plantamura	Changes related to amendment N°5

All documentation of **MaaT Pharma** is PROPRIETARY information and may not be forwarded to third parties without prior written consent from **MaaT Pharma.** Reproductions, either in part or in whole, may not be published or copied in any manner, without the explicit written consent of **MaaT Pharma**

TABLE OF CONTENTS

TABLE OF CONTENTS .... - 3 -
List of figures and tables .... - 6 -
Abbreviations .... - 7 -
ADMINISTRATIVE STRUCTURE AND CONTACT INFORMATION .... - 9 -
SYNOPSIS .... - 10 -
1. SCIENTIFIC JUSTIFICATION .... - 12 -
1.1. Introduction .... - 12 -
1.2. Microbiota and cancer .... - 13 -
1.3. Microbiota in hematology and allogenic stem cell transplantation .... - 15 -
1.4. Microbiota and hematological malignancies .... - 17 -
1.5. Strategies to restore microbiota homeostasis during intensive therapy for hematological malignancies .... - 18 -
1.6. Safety of fecal microbiota transplantation (FMT) .... - 19 -
1.7. Investigational medicinal product .... - 21 -
2. WORKING HYPOTHESIS AND PROPOSAL .... - 23 -
3. OBJECTIVES .... - 23 -
4. OUTCOME MEASUREMENTS .... - 24 -
4.1. Efficacy of AFMT in dysbiosis correction .... - 24 -
4.2. Efficacy of AFMT in MDRB eradication .... - 24 -
4.3. Definition of a dysbiosis biosignature .... - 24 -
4.4. Effect of dysbiosis correction on patient clinical status .... - 25 -
4.5. Measurement of AFMT safety .... - 25 -
4.6. Measurement of AFMT feasibility and acceptability .... - 26 -
5. SELECTION OF POPULATION FOR THE STUDY .... - 26 -
5.1. Inclusion criteria .... - 26 -
5.2. Exclusion criteria .... - 26 -
6. STUDY DESIGN .... - 27 -
6.1. Type of study .... - 27 -
6.2. Study duration .... - 27 -
6.3. Number of patients included in the study- Recruitment potential .... - 27 -
6.4. Experimental plan and practical procedures .... - 28 -
6.4.1. Study flow chart .... - 28 -
6.4.2. Conduct of the study 30
6.4.3. Management of analyses and samples 35
6.4.3.1. Routine analyses during patient care 35

All documentation of **MaaT Pharma** is PROPRIETARY information and may not be forwarded to third parties without prior written consent from **MaaT Pharma.** Reproductions, either in part or in whole, may not be published or copied in any manner, without the explicit written consent of **MaaT Pharma**

6.4.3.2. Analyses required for the study 36
6.4.3.3. Management of fecal samples 36
6.4.3.4. Processing of samples 37
7. STATISTICAL CONSIDERATIONS 39
7.1. Main endpoints 39
7.1.1. Evaluation of AFMT efficacy in dysbiosis correction 39
7.1.2. Evaluation of AFMT efficacy in MDRB eradication 39
7.2. Secondary endpoints 41
7.2.1. Definition of a dysbiosis signature 41
7.2.2. Effect of dysbiosis correction on patient clinical status 42
7.2.3. Safety variables 42
7.2.3.1. Adverse events 42
7.2.3.2. Laboratory data 43
7.2.3.3. Vital signs 43
7.3. Inferential aspects for criteria of efficacy 43
7.3.1. Co-primary endpoint: dysbiosis correction due to AFMT 43
7.3.2. Co-primary endpoint: MDRB eradication 43
7.3.3. Secondary analyses : Association between S and health status 43
7.4. Interim analyses 44
7.4.1. Interim analysis 44
7.4.2. Futility analysis 45
7.5. Individual patient Meta-analysis for a controlled result of efficacy 45
7.5.1. Principle 45
7.5.2. Inferential Aspects 46
7.5.3. Secondary analysis : Generalization 46
7.5.4. Limitations 46
7.6. Procedures 47
8. DATA MANAGEMENT 47
9. SAFETY EVALUATION 47
9.1. Adverse event reporting system 47
9.1.1. Definitions 47
9.1.2. Investigator responsibilities 49
9.1.2.1. Regulatory obligations of the investigator (Art. R. 1123-54) 49
9.1.2.2. Protocol specificities 50
9.1.2.2.1. Events that do not require an immediate report by the investigator 50
9.1.2.2.2. Events that require an immediate report by the investigator 50
9.1.2.3. Obligation of the Investigator regarding safety reporting 51

All documentation of **MaaT Pharma** is PROPRIETARY information and may not be forwarded to third parties without prior written consent from **MaaT Pharma.** Reproductions, either in part or in whole, may not be published or copied in any manner, without the explicit written consent of **MaaT Pharma**

9.1.2.3.1. Adverse events ... 51
9.1.2.3.2. Serious adverse events ... 51
9.1.2.3.3. AE follow-up ... 52
9.1.3. Promoter responsibilities ... 53
9.1.3.1. Analysis and report of SAE ... 53
9.1.3.2. Analysis and report of other safety data ... 54
9.1.4. Data Safety Monitoring Board ... 54
10. DIRECT ACCESS TO SOURCE DATA AND DOCUMENTS ... 55
11. QUALITY ASSURANCE AND QUALITY CONTROL ... 55
12. ETHICS AND LEGAL CONSIDERATIONS ... 56
12.1. Notification/ submission to regulatory authorities (ANSM) ... 56
12.2. Submission to ethics committee (CPP) ... 56
12.3. Amendments ... 57
12.4. Data protection approval ... 57
12.5. Information note and patient consent ... 57
12.6. Final research report ... 58
13. PROCESSING AND ARCHIVING DATA ... 58
14. INSURANCE ... 58
15. PUBLICATION POLICY ... 58
References ... 60

All documentation of **MaaT Pharma** is PROPRIETARY information and may not be forwarded to third parties without prior written consent from **MaaT Pharma.** Reproductions, either in part or in whole, may not be published or copied in any manner, without the explicit written consent of **MaaT Pharma**

List of figures and tables

Figure 1: Study flow chart .... - 28 -

Table 1: Summary of patient monitoring 34
Table 2: List of exams usually performed during patient care 35
Table 3: List of analyses requested for the study 36
Table 4: Fecal samples management 37

All documentation of **MaaT Pharma** is PROPRIETARY information and may not be forwarded to third parties without prior written consent from **MaaT Pharma.** Reproductions, either in part or in whole, may not be published or copied in any manner, without the explicit written consent of **MaaT Pharma**

Abbreviations

AD	Alternative Dysbiosis
ADR	Adverse Drug Reaction
AE	Adverse event
AFMT	Autologous Fecal Microbiota Transplantation
Allo-HSCT	Allogenic Hematopoietic Stem Cell Transplantation
AML	Acute Myeloid Leukemia
AML-M3	Acute promyelocytic Leukemia
ANSM	Agence National de Sécurité du Médicament et des produits de santé
APC	Adenomatous Polyposis Coli
CD	Crohn's Disease
CDI	*Clostridium difficile* infection
CMV	Cytomegalovirus
CNIL	Commission Nationale de l'Informatique et des Libertés
CNOM	Conseil National de l'Ordre des Médecins
COD	Cause of Death
CODECOH	COnservation D'Eléments du Corps Humain
CP	Conditional Power
CPP	Comité de Protection des Personnes
CRA	Clinical Research Associate
CRF	Case Report Form
CRO	Contract Research Organization
DNA	Deoxyribonucleic Acid
DSMB	Data Safety Monitoring Board
EMA	European Medicines Agency
ESBL	Extended Spectrum Beta Lactamase
FAS	Full Analysis Set
FMT	Fecal Microbiota Transplantation
GCP	Good Clinical Practices
GRE	Glycopeptide Resistant Enterococcus
GVHD	Graft Versus Host Disease
HBV	Hepatite B Virus
HCV	Hepatite C Virus
HIV	Human Immunodeficiency Virus
HR MDS	High-risk myelodysplastic syndrome
HSR	Health Status Related
HTLV	Human T-Lymphotropic Virus
IBD	Inflammatory Bowel Disease
IBS	Irritable Bowel Syndrome
IC	Immunocompromised
ICU	Intensive Care Unit
IgA	Immunoglobulin A
IPDM	Individual Patient Meta Analysis
MB	Microbiota
MDRB	Multi-Drug Resistant Bacteria
MRSA	Methicillin-Resistant *Staphylococcus aureus*
PCR	Polymerase Chain Reaction
RCT	Randomized Controlled Trial
RNA	Ribonucleic Acid
ROS	Reactive Oxygen Species
RPPS	Répertoire Partagé des Professionnels de Santé
SADR	Serious Adverse Drug Reaction

All documentation of **MaaT Pharma** is PROPRIETARY information and may not be forwarded to third parties without prior written consent from **MaaT Pharma.** Reproductions, either in part or in whole, may not be published or copied in any manner, without the explicit written consent of **MaaT Pharma**

SAE	Serious Adverse Event
SAP	Statistical Analysis Plan
SMD	Standard Mean Deviation
SOP	Standard Operating Procedure
SUSAR	Suspected Unexpected Severe Adverse Reaction
TBI	Total Body Irradiation
TEAE	Treatment-Emergent Adverse Events
TH	T Helper
UC	Ulcerative Colitis
VRE	Vancomycin Resistant Enterococcus

All documentation of **MaaT Pharma** is PROPRIETARY information and may not be forwarded to third parties without prior written consent from **MaaT Pharma.** Reproductions, either in part or in whole, may not be published or copied in any manner, without the explicit written consent of **MaaT Pharma**

ADMINISTRATIVE STRUCTURE AND CONTACT INFORMATION

Sponsor information

Sponsor: MaaT Pharma, 317 avenue Jean Jaurès, 69007 LYON

Project Director: David Salako – dsalako@maat-pharma.com

Project Manager : Emilie Plantamura – eplantamura@maat-pharma.com

Medical Advisors: Etienne Varlan– evarlan@maat-pharma.com

Mohamad Mohty – mohamad.mohty@inserm.fr

Biostatistician : Professor Dr Philippe Lehert - philippe.lehert@gmail.com

Contract research organization (CRO):

- Global management: **AXONAL / BIOSTATEM –** odyssee@axonal.com
  Le Clémenceau 2
  215 avenue Georges Clémenceau
  F-92024 Nanterre Cedex, France
- Central Laboratory: **BIOFORTIS SAS,**
  3 route de la Chatterie
  44800 Saint Herblain, France.

Pharmacovigilance: **VIGIPHARM**
265 rue Maurice Béjart
34080 Montpellier, France

Study committee:

Data Safety Monitoring Board (see DSMB charter)

All documentation of **MaaT Pharma** is PROPRIETARY information and may not be forwarded to third parties without prior written consent from **MaaT Pharma.** Reproductions, either in part or in whole, may not be published or copied in any manner, without the explicit written consent of **MaaT Pharma**

SYNOPSIS

TITLE	PreventiOn of DYSbioSis complications with autologous fecal microbiota transplantation in acutE myeloid leukemia patients undergoing intensive treatment: a feasibility and safety study (ODYSSEE STUDY)
SPONSOR	**MaaT Pharma**
PRINCIPAL INVESTIGATOR	Pr Ollivier LEGRAND<br>Hématologie Clinique et Thérapie Cellulaire<br>Hôpital Saint-Antoine<br>Assistance Publique - Hôpitaux de Paris<br>184, rue du faubourg Saint-Antoine – 75012 Paris
PROTOCOL VERSION	V10.0
STUDY DESIGN	Single-arm multicenter prospective interventional trial in hospitalized patients (Phase I/II).
PRIMARY OBJECTIVE(S)	Efficacy of autologous fecal microbiota transplantation (AFMT) in dysbiosis correction and eradication of multi-drug resistant bacteria (MDRB) in patients with acute myeloid leukemia (AML)or high-risk myelodysplastic syndrom (HR MDS) undergoing chemotherapy and antibiotherapy
SECONDARY OBJECTIVES	1- Definition of a dysbiosis biosignature<br>2- Effect of dysbiosis correction on patient clinical status<br>3- Short and mean term safety of AFMT in AML / HR MDS patients receiving intensive chemo- and antibiotherapy and receiving AFMT intervention during hospitalization<br>4- Feasibility of the AFMT procedure and acceptability by the patient
POPULATION	Consenting AML or HR MDS patients at participating sites undergoing chemotherapy and antibiotherapy
INCLUSION CRITERIA	- Patients ≥ 18 and ≤ 75 years old with *de novo* diagnosis of AML or HR MDS for whom intensive induction chemotherapy is anticipated within 10 days after admission<br>- Patients willing to donate stool samples and to follow protocol recommendations<br>- Signature of informed and written consent
NON INCLUSION CRITERIA	- Acute promyelocytic leukemia (AML-M3)<br>- Known allergy or intolerance to trehalose or maltodextrin<br>- Pregnancy: positive urinary or blood test in female of chilbearing potential<br>- Severe disease with a life expectancy < 3 months<br>- Other ongoing interventional protocol that might interfere with the study<br>- Non eligibility for collection of autologous stools upon admission:<br>* Patients refusing to consent<br>* Antibiotherapy at the time of study inclusion ≥ 4 days<br>* Concomitant or previous diagnosis of a significant inflammatory bowel disease (UC, CD) or other progressive digestive disease requesting treatment or further medical exploration

All documentation of **MaaT Pharma** is PROPRIETARY information and may not be forwarded to third parties without prior written consent from **MaaT Pharma.** Reproductions, either in part or in whole, may not be published or copied in any manner, without the explicit written consent of **MaaT Pharma**

	* Presence of severe colitis of any etiology at the time of admission or severe digestive disorders (acute or chronic diarrhea) within 3 months preceding inclusion<br>* Presence of blood in faeces collected at the time of inclusion<br>* Patient getting a recent colonoscopy (within 3 months preceding inclusion)<br>- Detection of MDRB, pathogenic bacteria, parasites, norovirus and/or rotavirus during screening of autologous stool collected at baseline<br>- Non eligibility for inoculum transplantation: persistent mucositis, colitis, or haemorrhoids, presence of blood in more than 1 patient's faeces out of 3 the week preceding the transplantation<br>- Non feasibility of inoculum procedure: patient refusal; technical or biological mismatch of the inoculum<br>- Absence of effective contraceptive method for female of childbearing potential<br>- Lactation<br>- Inability to give an informed consent
VARIABLES	Outcome measurements :<br>- Dysbiosis measurement and characterization<br>- MDRB detection<br>- Health status measurement<br>- Biochemical analyses, immunomonitoring<br>- Measurement of safety and feasibility of AFMT
STUDY SIZE	20 subjects
STATISTICAL CONSIDERATIONS	- Main endpoints: evaluation of AFMT efficacy in dysbiosis correction and MDRB eradication<br>- Secondary endpoints:<br>* Definition of a dysbiosis biosignature<br>* Effect of dysbiosis correction on patient health status<br>* Safety and feasibility/acceptability evaluation of AFMT
TIME SCHEDULE	- Total duration of the study : 24 months<br>- Inclusion period : Maximum 12 months<br>- Participation period for a patient :<br>- main period : 70 days<br>- follow-up period: 1 year

All documentation of **MaaT Pharma** is PROPRIETARY information and may not be forwarded to third parties without prior written consent from **MaaT Pharma.** Reproductions, either in part or in whole, may not be published or copied in any manner, without the explicit written consent of **MaaT Pharma**

1. SCIENTIFIC JUSTIFICATION

1.1. Introduction

Humans represent a scaffold on which diverse microbial ecosystems are established. Immediately after birth, all mammals are initiated into a life-long process of colonization by foreign microorganisms that inhabit most environmentally exposed surfaces (such as the skin, mouth, gut and vagina) (1, 2). Shaped by millennia of evolution, some host–bacterial associations have developed into beneficial relationships, creating an environment for mutualism. A key example of such environment is provided by the vast numbers and diversity of bacteria that are found in the lower gastrointestinal tract of mammals (1, 3, 4). By young adulthood, both humans and other mammals support one of the most complex microbial ecosystems on the planet, with over 100 trillion bacteria in the distal gut (5, 6).

Symbiotic bacteria of the mammalian gut have long been appreciated for the benefits they provide to the host: they supply essential nutrients, metabolize indigestible compounds, defend against colonization by opportunistic pathogens and even contribute to the development of the intestinal architecture (7). Moreover, it seems that certain basic developmental features and functions of the mammalian immune system depend on interactions with the human microbiota (8). Unlike opportunistic pathogens, which elicit immune responses that result in tissue damage during infection, some symbiotic bacterial species have been shown to prevent inflammatory diseases during colonization. Surprisingly, the ‘normal’ microbiota also contains microorganisms that have been shown to induce inflammation under particular conditions. Therefore, the microbiota has the potential to exert both pro- and anti-inflammatory responses, and the composition of the bacterial communities in the gut may be intimately linked to the proper functioning of the immune system.

More than 80% of the gut microbiota cannot be cultivated under conventional culture techniques, leading their identification and functional analysis difficult. In recent years, deep-sequencing technology has made it possible to characterize the composition of intestinal microbial contents free of the selective biases of culture-based methods. Sequencing of bacterial ribosomal ribonucleic acid (rRNA) gene was developed in the 1970s (9). This gene was chosen because it strikes an appropriate balance of conservation and variability with enough variation present to distinguish between different species and strains, yet enough similarity to identify members belonging to the same phylogenetic group. In combination with subsequent genetic methodologies, including in situ hybridization and polymerase chain

All documentation of **MaaT Pharma** is PROPRIETARY information and may not be forwarded to third parties without prior written consent from **MaaT Pharma.** Reproductions, either in part or in whole, may not be published or copied in any manner, without the explicit written consent of **MaaT Pharma**

reaction (PCR), this method allows rapid identification of bacterial isolates from clinical samples (10). Recently, high-throughput sequencing technologies, so-called deep-sequencing methods allow investigators to characterize the composition of mixed bacterial samples. This has led to a surge of interest investigating how bacteria can contribute to health and disease.

1.2. Microbiota and cancer

The relationship between cancer and microbes is complex. Although cancer is generally considered to be a disease of host genetics and environmental factors, microorganisms are implicated in 20% of human malignancies (11). Microbes present at mucosal sites can become part of the tumor microenvironment of aero-digestive tract malignancies, and intratumoral microbes can affect cancer growth and spread in many ways: either by altering the balance of host cell proliferation and death, by guiding immune system function, or by influencing metabolism of host-produced factors, ingested food, and pharmaceutical agents (12).

There is increasing evidence that inflammation may play a critical permissive role at all stages of cancer development and progression. Consequently, diseases that are characterized by chronic inflammation are often associated with a marked increase in cancer incidence (e.g. colorectal carcinoma in inflammatory bowel disease). Given the ability of the commensal microbiota to modulate inflammation, it is reasonable to consider whether the microbiota contributes to the pathogenesis of human carcinogenesis (13). Among the cancers that have been linked to the microbiota are those involving the gastrointestinal tract and especially the colon where most of the microbes congregate, rather than the small intestine. Sporadic colorectal cancer is typically initiated by somatic mutations in the adenomatous polyposis coli (APC) gene and when transmitted as a loss-of-function germ-line mutation is conferred in a Mendelian fashion and associated with early-onset familial colorectal cancer. Mice that are heterozygous for mutated APC (APCMin/+) are the most tractable model for studying gene-environment interactions in the development of colorectal cancer because they develop polyps upon loss of heterozygosity of the wild-type APC allele and cancers upon the accumulation of additional mutations (14). Colonization of mice with enterotoxin-producing *B. fragilis*, a symbiont with pathogenic capabilities which is often carried by asymptomatic individuals, can promote cancer development in APCMin/+ mice (15). This occurs through β-catenin–driven epithelial cell proliferation by virtue of the toxin's ability to degrade E-cadherin (16). It is therefore possible that pathobionts and pathogens are important cofactors in the development

All documentation of **MaaT Pharma** is PROPRIETARY information and may not be forwarded to third parties without prior written consent from **MaaT Pharma.** Reproductions, either in part or in whole, may not be published or copied in any manner, without the explicit written consent of **MaaT Pharma**

of colorectal neoplasia. Whether this occurs through indirect (for example, effects on innate or adaptive immune functions even in the absence of overt inflammation or altered xenobiotic metabolism in conjunction with other environmental factors) and/or direct (for example, phenotypic exacerbation of an existing mutation or induction of a second genetic mutation) mechanisms is unknown. In humans, analysis of the microbiota in colon cancer tissue versus control tissue has also led to two independent reports of increased representation of *Fusobacterium* spp. and, in particular, *Fusobacterium nucleatum*, which has previously been associated with periodontal disease (17). Whether these bacteria actually play a role in tumor development or progression or simply adapt to the intestinal cancer niche is not known. It has recently been suggested that particular members of the microbiota, such as *B. fragilis* discussed above, in addition to having virulence factors, may remodel the microbiome, favoring inflammatory responses that promote epithelial cell transformation, leading to cancer (18). This in turn may foster the emergence of additional microbes that might be considered as “carcinogenic allies”.

Moreover, gut microbiota can also influence toxicity and metabolism of drugs. Thus, irinotecan is a topoisomerase-1 inhibitor that is used in combination with other chemotherapeutic agents to treat some cancers. Microbial-produced β-glucuronidase regulates levels of irinotecan’s bioactive form within the intestinal lumen and thus influences irinotecan’s toxicity. Oral bacterial β-glucuronidase inhibitors blunt the dose-limiting toxicities of irinotecan in mice and do not harm host cells or kill bacteria, which suggests that microbial metabolism is a plausible target in cancer care (19). The gut microbiota also affects the efficacy of chemotherapy. Oxaliplatin is a platinum-based chemotherapy used to treat several gastrointestinal malignancies. Together, the microbiota and immune system contribute to oxaliplatin’s efficacy (20). Thus, the gut microbiota primes myeloid cells for high-level Reactive oxygen species (ROS) production. The resultant intratumoral oxidative stress augments oxaliplatin-associated desoyribonucleic acid (DNA) damage, triggering cancer cell death (20). Cyclophosphamide, an alkylating agent used in hematologic malignancies and solid tumors, can injure the small intestinal epithelium. The ensuing barrier breach results in gut microbiota–dependent, T helper (TH) cell–mediated antitumor responses (21). Delineating the roles of gut microbiota in response to chemotherapy in model systems and undertaking epidemiologic studies with microbiome analysis in patients with and at risk for cancer will be critical for

All documentation of **MaaT Pharma** is PROPRIETARY information and may not be forwarded to third parties without prior written consent from **MaaT Pharma.** Reproductions, either in part or in whole, may not be published or copied in any manner, without the explicit written consent of **MaaT Pharma**

establishing the microbiota as an adjuvant therapy that enhances efficacy or attenuates toxicity of chemotherapies.

1.3. Microbiota in hematology and allogenic stem cell transplantation

Since the early 1970s, researchers have known that the commensal bacteria residing in the intestines, collectively termed the intestinal microbiota, are important mediators of the biology of allogenic hematopoietic stem cell transplantation (allo-HSCT) (22). Early studies in mice and humans suggested a link between an individual's intestinal microbiota and its propensity for graft-versus-host-disease (GVHD), the most important complication after allo-HSCT. These were followed by clinical trials that reported less GVHD when allo-HSCT was performed in an isolated, protective environment with laminar airflow and gut decontamination (23). Subsequent studies, however, could not confirm a clear benefit of these protective environments, and the practice of laminar airflow isolation was abandoned by some centers in the early 1990s (24). The reasons behind these inconsistent results remain unclear but could be due to variable success in total decontamination in the gut. In the early studies, mice transplanted in germ-free conditions (25) or treated with gut-decontaminating antibiotics (22) developed significantly less GVHD, which demonstrated that the microbiota can contribute to the development of GVHD-related lethality. Reports from Germany (26) and the Netherlands (27) showed that prophylactic complete gut decontamination prevented acute GVHD. Another study of the prophylactic use of the broad-spectrum antibiotic meropenem during episodes of neutropenia or fever reported a favorable effect on the morbidity of allo-HSCT (28). Clinical strategies to suppress the intestinal microbiota in an attempt to prevent GVHD initially showed considerable promise (29, 30); however, these strategies failed to demonstrate a consistent benefit (31-33). Thus, the best means of preventing GVHD by modulating intestinal microbiota have remained unclear. In the current era of rapidly developing new technologies for deep-sequencing of 16S rRNA, the microbiota has been re-examined in relation to allo-HSCT. Several groups have recently discovered important relationships between the microbiota and outcomes in allo-HSCT recipients (34-36).

Pre-transplantation conditioning regimens, which often include combinations of chemotherapy and total body irradiation (TBI), are critical for the success of transplants because they allow engraftment of allogeneic hematopoietic cells and often also treat the underlying malignancy. However, conditioning also disrupts the delicate interplay between host

All documentation of **MaaT Pharma** is PROPRIETARY information and may not be forwarded to third parties without prior written consent from **MaaT Pharma.** Reproductions, either in part or in whole, may not be published or copied in any manner, without the explicit written consent of **MaaT Pharma**

and microbiota by way of mucositis, other organ dysfunction, and increased susceptibility to infection. While mice transplanted in the absence of GVHD exhibit only mild changes in microbiota composition, murine GVHD is associated with many specific changes in the intestinal microbiota, including a loss of microbial diversity, dysbiosis (imbalance of intestinal microbiota), and the expansion of the bacterial Lactobacillales orders (including *Lactobacillus*, *Enterococcus*, and *Streptococcus* species) or Enterobacteriales (including *Escherichia*, *Klebsiella*, and *Enterobacter* species), the latter of which may adversely impact on GVHD. This is also accompanied by a corresponding loss of anaerobic bacteria from the phylum Firmicutes, including members of the Clostridiales order (36-38). In contrast to these findings in mice, allo-HSCT uncomplicated by GVHD in humans is associated with major changes in microbiota composition (39). This species difference may be explained by the frequent administration of antibiotics in patients after allo-HSCT, whereas antibiotics are not commonly used in murine models of allo-HSCT experiments. Thus, *Enterococcus*, *Streptococcus*, and various Enterobacteriales are commonly expanded after allo-HSCT, and their increased abundance can precede bloodstream infections by the same organism (40). Moreover, exposure to metronidazole during allo-HSCT increases the risk for developing enterococcal expansion. These findings may suggest that reduction of obligate anaerobic commensals leads to impaired suppression of Enterococcus. Separate from antibiotics, how might GVHD itself affect the intestinal microbiota? Recent reports indicate that the abundance of certain bacteria that play important homeostatic roles, especially Clostridiales, could be affected during GVHD. In concert with observations made in mice, patients who develop GVHD display microbiota shifts away from dominance of Clostridiales species to dominance by Lactobacillales or Enterobacteriales (35, 36). Furthermore, a stronger association was observed with death due to transplant-related causes, with no discernible association with mortality arising from relapse or progression of disease, suggesting that lack of microbial diversity is primarily linked to transplant-related deaths such as infection and GVHD (41). Interestingly, changes in nutritional intake, especially the malnutrition that plagues transplant patients, might be an explanation for these shifts in intestinal microbiota composition, as a pattern of loss of Clostridiales can be observed in volunteers given high-protein and low-carbohydrate diets (42) or diets derived entirely from animal products (43). The findings of bacterial shift from Clostridiales observed during GVHD may reflect maladaptation of these bacteria, given the fact that many of the Clostridiales perform hydrolysis and the bulk of fermentation of consumed non-digestible

All documentation of **MaaT Pharma** is PROPRIETARY information and may not be forwarded to third parties without prior written consent from **MaaT Pharma.** Reproductions, either in part or in whole, may not be published or copied in any manner, without the explicit written consent of **MaaT Pharma**

carbohydrates. Their metabolites are thought to produce health benefits (43), and in the setting of GVHD, changes in diet of the patients can affect metabolic functions of the bacteria. Clostridial species were recently found to prevent inflammation by upregulating regulatory T cells in the intestines (44), which invites speculation that GVHD may deplete anti-inflammatory cell populations by reducing the abundance of Clostridiales. Gut decontamination continues to be used in many centers, but there is no consensus regarding ideal choice of antibiotic coverage and its benefits remain controversial. Careful studies focusing on effects of different-spectrum antibiotics on bacterial commensals and transplant-related outcomes are needed.

1.4. Microbiota and hematological malignancies

While there is a growing body of evidence highlighting the interactions between the microbiota and allo-HSCT outcomes, little information is available about the role of the microbiota in patients with hematological malignancies treated with intensive chemotherapy outside the allo-HSCT setting (e.g. acute myeloid leukemia -AML- or high-risk myelodysplastic syndrom - HR MDS - patients).

AML are rare, but potentially fatal cancers in the adults. After diagnosis, treatment of AML in fit patients, will include an induction phase with intensive chemotherapy, followed by one or several cycles of intensive consolidation that may or may not include stem cell transplantation. The different treatment phases of AML or HR MDS require prolonged hospital stays in a protected environment due to the high risk of infectious complications. Indeed, opportunistic infections are frequent in these patients and can be life-threatening despite the use of prophylactic antibiotic treatments (gut decontamination, probabilistic antibiotherapy in case of fever of unknown origin, etc). Currently, no microbiological screening upon patient admission is mandatory but in practice, several analyses can be done according to the physician (bacteriological screening in case of fever; parasitological and viral (HIV, HCV, HBV, HTLV, CMV) screening depending on the context (patient's origin, international travels). Screening for MDRB (Multi-Drug Resistant Bacteria) is becoming more common in hospitals due to the arrival of patients from various geographic origins and would help to orientate effective antibiotherapy during chemotherapy-induced aplasia.

Treatments of hematological malignancies are recently known to impact gut microbial composition. Diversity indices are mathematical measures of species diversity in a community

All documentation of **MaaT Pharma** is PROPRIETARY information and may not be forwarded to third parties without prior written consent from **MaaT Pharma.** Reproductions, either in part or in whole, may not be published or copied in any manner, without the explicit written consent of **MaaT Pharma**

and provide information about bacterial microbiota composition such as total numbers (richness), proportions and evenness of species. These indices (Simpson, Simpson reciprocal and Shannon indexes) are simple tools used by bacteriologists to estimate microbial diversity that is known to be affected and reduced in case of dysbiosis and related to several diseases (45). Interestingly, recent study examined the microbial intestinal diversity of 80 patients receiving allo-HSCT with a conditioning regimen of chemotherapy and broad-spectrum antibiotics (41). Fecal samples were collected within 7 days following stem cell engraftment and subjects were classified using inverse Simpson index into high (>4), intermediate (2-4) and low (<2) diversity groups. Overall survival was followed within a 3-years follow-up period and was significantly different between the 3 groups. Mortality outcomes were significantly worse in patients with lower intestinal diversity with in particular a strong effect on mortality after multivariate adjustment for other clinical predictors. Overall survival at 3 years was 36%, 60%, and 67% for low, intermediate, and high diversity groups, respectively, indicating that intestinal microbiota diversity at engraftment is an independent predictor of mortality in allo-HSCT recipients.

Furthermore, composition and function of gut microbial communities were shown to be deeply impacted by chemotherapy without any antibiotherapy in a cohort study on 28 patients with non-Hodgkin lymphomas. Fecal samples collected before and after chemotherapy were analyzed by 16S sequencing. Alpha and beta-diversity comparisons of the gut microbiomes revealed taxonomic and functional shifts following chemotherapy (46). The latter results strongly suggest that not only antibiotherapy but also chemotherapy itself can modify the gut microbiota composition during hematological malignancies treatment, with subsequent consequences on co-morbidities.

1.5. Strategies to restore microbiota homeostasis during intensive therapy for hematological malignancies

Indeed, the development of strategies to manipulate the gut microbiota to suppress or decrease treatment-related complications in such patients would be a welcome addition to the standard of care armamentarium. Experience from studies in murine models found that certain intestinal Lactobacilli can reduce experimental GVHD (36) and allow for decreased severity of GVHD, as well as improved survival of recipients following the administration of probiotic bacteria (47).

All documentation of **MaaT Pharma** is PROPRIETARY information and may not be forwarded to third parties without prior written consent from **MaaT Pharma.** Reproductions, either in part or in whole, may not be published or copied in any manner, without the explicit written consent of **MaaT Pharma**

Thus, replenishing the microbiota through probiotic therapy may potentially offer a novel approach to attenuate toxicity and its associated risk for bloodstream infections. Fecal transplantation appears to be highly effective, with multiple studies reporting response rates approaching 90% in *Clostridium difficile* infected patients with *C. difficile* toxi-infection recurrences (48). Thus, pioneering physicians performed fecal transplants from healthy donors, often close relatives, to patients with recurrent *C. difficile* infections (49). The results were highly effective in roughly 90% of cases (50, 51). Recently, controlled studies demonstrated the effectiveness of fecal transplantation compared with conventional antibiotic administration (52, 53). Resolution of diarrhea associated with *C. difficile* infection without relapse after 10 weeks occurred in 93.8% of patients cured by infusion of donor faeces compared to patients with vancomycin (30.8%). Fecal microbiota in patients with *C. difficile* infection had a reduced bacterial diversity evaluated by the Simpson's reciprocal index, as compared with healthy persons. Infusion of donor faeces resulted in improvement in the microbial diversity that persisted over time, with quantitative changes in relevant groups of Bacteroidetes, Clostridium and Proteobacteria species (52).

Thus, it would be very encouraging if strategies to manipulate the gut microbiota could be developed to produce favorable conditions that could minimize treatment-related toxicities in patients with hematological malignancies undergoing intensive chemotherapy. Microbiota diversity could be a surrogate endpoint for monitoring dysbiosis correction after fecal microbiota transplantation.

1.6. Safety of fecal microbiota transplantation (FMT)

Recently, Rossen *et al*. performed a systematic review to analyse the clinical efficacy and safety of FMT used as clinical therapy (54). They were able to identify 45 studies; 34 on *Clostridium difficile*-infection (CDI), 7 on inflammatory bowel disease, 1 on metabolic syndrome, 1 on constipation, 1 on pouchitis and 1 on irritable bowel syndrome (IBS). In CDI, 90% resolution of diarrhea in 33 case series (n = 867) was reported, and 94% resolution of diarrhea after repeated FMT in a randomized controlled trial (RCT) (n = 16). In ulcerative colitis (UC) remission rates of 0% to 68% were found (n = 106). In Crohn's disease (CD) (n = 6), no benefit was observed. In IBS, 70% improvement of symptoms was found (n = 13). Reversal of symptoms was observed in constipation (100%) (n = 3). In pouchitis, none of the patients (n = 8) achieved remission. One randomized controlled trial (RCT) showed significant improvement

All documentation of **MaaT Pharma** is PROPRIETARY information and may not be forwarded to third parties without prior written consent from **MaaT Pharma.** Reproductions, either in part or in whole, may not be published or copied in any manner, without the explicit written consent of **MaaT Pharma**

of insulin sensitivity in metabolic syndrome (n = 10). Serious adverse events (SAE) were rare. SAE were reported in 34 out of 45 studies. In total, 35 (3.4%, all CDI cases) of 1029 patients, were reported to have died and 10 (0.97%) (out-) patients were hospitalised during follow-up. One patient died from aspiration during sedation for FMT administered *via* colonoscopy, which was considered to be related to the FMT procedure (54). Four patients were reported to have died from complicated CDI with small bowel involvement confirmed at autopsy (*n* = 1), a toxic megacolon due to persistent CDI one month after FMT (*n* = 1), and complicated CDI not further specified (*n* = 2) (55, 56). A severely ill patient treated with FMT for CDI, died of a peritonitis which could be related to treatment (57). In the other 29 patients the cause of death was not related to CDI illness or of unknown cause. Reasons for hospitalisation included: cecal perforation during FMT treated with colectomy (*n* = 1), symptomatic choledocholithiasis (*n* = 1) and not further specified in eight patients (52). Reported SAE associated with FMT were mostly self-limiting and occurred frequently within hours after infusion. Intestinal reported symptoms were: bloating, flatulence, belching and abdominal cramps, remaining IBS-like symptoms after CDI clearance post FMT, abdominal discomfort, irregularity of bowel movements and vomiting. In 11 patients (all treated for inflammatory bowel diseases (IBD); three for CD and eight for ulcerative colitis (UC)) fever, without other clinical symptoms or signs of sepsis, was reported during and up to one day after FMT (58-60). No causative agents were identified by blood culture, but a rise in C-reactive protein was measured in some of these patients. Fever disappeared within 3 days in all patients. Withdrawal due to treatment intolerance (leaking of enemas for three days) occurred in one adolescent (59). The 2 included RCTs reported mild adverse events attributed to FMT, including diarrhea, cramping, belching, nausea, abdominal pain, bloating, transient fever, and dizziness (53, 61-64). Serious adverse events were also described, including a case of Fournier gangrene (53), but none were attributed to the FMT. Case-series studies reported few harms. Eight case-series studies (reporting on 70 patients) did not explicitly mention harms; all others specifically commented on harms (or lack thereof). Possible procedure-related harms, including microperforation with colonoscopy (65) and gastrointestinal bleeding (66), peritonitis and pneumonia (57) with use of the upper gastrointestinal tract route, were rarely reported. One case-series study reported on FMT use in 80 immunocompromised patients (67). Although serious adverse events (2 deaths and 10 hospitalizations) occurred in 12 patients (15%), none seemed to be directly related to FMT. Non-serious adverse events were reported for 12 patients (15%); 4 were considered related

All documentation of **MaaT Pharma** is PROPRIETARY information and may not be forwarded to third parties without prior written consent from **MaaT Pharma.** Reproductions, either in part or in whole, may not be published or copied in any manner, without the explicit written consent of **MaaT Pharma**

to FMT, 5 were possibly related and three were unrelated to FMT. Because rare adverse events may be first reported as a case report, case reports for such events were also examined. One report described a patient with abdominal pain and hypotension 3 days after FMT by means of a gastrojejunostomy tube placed through an indwelling gastric tube (68). The patient had pneumoperitoneum, toxic megacolon, and polymicrobial bacteremia and subsequently died. Other potential harms included development of herpes zoster 2 months after FMT (69), recurrence of *Escherichia coli* bacteremia (70), a flare of previously quiescent ulcerative colitis (71), and 2 cases of norovirus gastroenteritis (72). In one of these cases, an endoscopy suite employee had norovirus-like symptoms the day before the FMT was administered in the suite. Long-term data on harms were not reported. Overall, based on the above studies, the safety profile of FMT proved to be excellent.

Litterature on FMT in immunocompromised patients is still scant, however preliminary data are encouraging. A retrospective study done on immunocompromised (IC) and non-immunocompromised (non-IC) patients who received FMT for recurrent CDI concludes that response to FMT is equivalent in IC and non-IC population. When comparing the percentage of SAEs between IC and non-IC patients, no significant difference was found (73). Until today, 15 published case reports or case series have described the use of FMT in IC patients for a total of 132 treated patients. Even if further prospective studies are warranted to fully evaluate the role and safety of FMT in IC patients, experience to data does not suggest these patients are at increased risk of infective complications (74).

1.7. Investigational medicinal product

During heterologous FMT, the possibility of transmission of infectious agents or of disease conditions may be related to change of the gut microbiota: a recent example was the transmission of weight gain from an obese donor of FMT to a lean receiver (75). It is known that transmission of human fecal microbiota from obese subjects to experimental rodents will transmit an obese and/or insulin resistant phenotype.

In France, a preparation conditioned for FMT is considered as a drug and therefore must follow the GCP (Good Clinical Practice) recommendations related to the medicament definition: special recommendations were published in March 2014 and June 2015 in a public document by the French agency (ANSM), written by experts (76, 77) with the purpose of

All documentation of **MaaT Pharma** is PROPRIETARY information and may not be forwarded to third parties without prior written consent from **MaaT Pharma.** Reproductions, either in part or in whole, may not be published or copied in any manner, without the explicit written consent of **MaaT Pharma**

minimizing the risk associated in FMT when a clinical research is engaged: fecal transplantation will follow before patients delivery, pharmaceutical preparation rules and framework.

In allogenic FMT, prior to donation, donors must undergo a thorough medical evaluation and laboratory screening to avoid the patient contamination. In the context of autologous FMT, screening will include the detection of gastrointestinal pathogens (multidrug resistant bacteria, pathogenic bacteria and parasites, cf paragraph 6.4.3.4) and norovirus / rotavirus that are intestinal viruses responsible for severe diarrhea and other gastrointestinal symptoms. The detection of one of these pathogens during screening of stool collected upon patient's admission will constitute an exclusion criterion of the study.

Critical points concerned mainly product preparation and donor's selection as well as the traceability process to establish when fecal microbiota transplantation is proposed to a patient. Unlike heterologous FMT, autologous FMT (AFMT) will have the advantage of presenting a simple traceability of the preparation and control of the inoculum during donor procedures and avoid fecal banking with multiple donors: AFMT may reduce the risk of potential transmission of diseases originated from a microbiota of an external donor.

As of today, only one study is available to compare efficacy of fresh and frozen feces in FMT (78). Both approaches show similar effect in the resolution of *C. difficile* induced diarrhea.Two other publications showed that frozen fecal material (conservation at -80°C) could be used with a similar efficiency compared to fresh material (53, 79). These two studies are limited as they do not provide comparison between frozen and fresh material but clearly demonstrate the efficacy of this approach in clearing recurrent CDI.

In our preclinical studies, we validated on murine models that microbiota reconstitution after re-implantation of a frozen inoculum with our cryoprotective diluent has comparable phylogenetic and metabolomic profile compared to the control (microbiota reconstitution with a fresh inoculum). Moreover, we validated conditions for the re-conditioning of frozen preparations to be later used for re-administration. Rapid thawing at 37°C appears as the modality that will favor the most favorable conditions for the reconstruction of a microbiota close to the initial stool compared to 4°C overnight thawing (see Investigational medicine product dossier (80)).

All documentation of **MaaT Pharma** is PROPRIETARY information and may not be forwarded to third parties without prior written consent from **MaaT Pharma.** Reproductions, either in part or in whole, may not be published or copied in any manner, without the explicit written consent of **MaaT Pharma**

In allogenic FMT, doses of faeces are usually around 50g per transplantation with delivery methods such as naso-gastric, naso-enteric, trans-colonoscopic, enema route or a combination of these. Our preclinical studies on pigs did not revealed any dose effect. We hypothetized that autologous FMT would allow a better colonization, as ecological niches with different microorganisms are already known by the host immune system. We therefore propose in our protocol 2 transplantations (with 30g of faeces each, with a 150mL corresponding volume of inoculum) allowing a first colonization followed by a strengthening of the system for the least abundant germs. We chose enema delivery method for our patients, other delivery routes having important side effects (risk of colonic perforation, undesirable additional anesthesia for leukemic patients) (see Investigator brochure (81)).

2. WORKING HYPOTHESIS AND PROPOSAL

We propose to use autologous transplantation of fecal microbiota to AML or HR MDS patients treated with intensive chemotherapy and antibiotics in order to restore the balance of their intestinal microbiome and thereby eradicate treatment-induced MDRB, infection-related complications, as well as sequelae to the gastrointestinal tract. Therefore, we propose to perform a single-arm multicentre prospective FMT trial in AML or HR MDS patients receiving intensive chemotherapy, and who are usually heavily treated with broad-spectrum antibiotics during aplasia that generate a profound status of dysbiosis. For this purpose, at the time of admission and diagnosis, patients will be requested to donate stools that will be comprehensively screened, and if deemed appropriate according to protocol criteria, conditioned and stored frozen until future processing and transplantation after aplasia completion.

3. OBJECTIVES

- Co-primary objectives:
  - Efficacy of AFMT in dysbiosis correction in AML or HR MDS patients undergoing chemotherapy and antibiotherapy
  - Efficacy of AFMT in MDRB eradication
- Secondary objectives:
  - Definition of a dysbiosis biosignature

All documentation of **MaaT Pharma** is PROPRIETARY information and may not be forwarded to third parties without prior written consent from **MaaT Pharma.** Reproductions, either in part or in whole, may not be published or copied in any manner, without the explicit written consent of **MaaT Pharma**

- Effect of dysbiosis correction on patient clinical status
- Short and mean term safety of AFMT in AML or HR MDS patients receiving intensive chemo- and antibiotherapy and receiving AFMT intervention during hospitalization
- Feasibility of the AFMT procedure and acceptability by the patient

4. OUTCOME MEASUREMENTS

4.1. Efficacy of AFMT in dysbiosis correction

Monitoring of microbiota deterioration following chemotherapy / antibiotherapy and dysbiosis correction after AFMT will be evaluated using indexes commonly used to characterize (Richness and diversity (such as Simpson or Shannon indexes) and between sample comparisons of the microbiota composition (Bray-Curtis distance, Sorensen, Pearson or Spearman indexes).

Microbiome diversity and richness will be measured at baseline, prior to the start of the induction chemotherapy, after induction chemotherapy/ antibiotherapy, post-AFMT and after consolidation chemotherapy/antibiotherapy. Metagenomic results of the 4 sequential samples will be compared in order to evaluate AFMT efficacy.

4.2. Efficacy of AFMT in MDRB eradication

Based on bacterial culture and analysis of resistance gene expression by metagenomics, we will evaluate the quantity of MDRB before, after chemotherapy/antibiotherapy and after AFMT.

4.3. Definition of a dysbiosis biosignature

A dysbiosis biosignature will be defined using association of:

- An innovative MaaT index based on microbial ecology (prevalence of pre-identified families / Genera / orders of bacteria ponderated by their beneficial or pathogenic effect on the host)
- Fecal and circulating inflammatory markers: calprotectin, neopterin, CRP
- Fecal marker of intestinal barrier integrity: zonulin

All documentation of **MaaT Pharma** is PROPRIETARY information and may not be forwarded to third parties without prior written consent from **MaaT Pharma.** Reproductions, either in part or in whole, may not be published or copied in any manner, without the explicit written consent of **MaaT Pharma**

- Monitoring of blood immune cells
- Patient's clinical status (gastro-intestinal symptoms: nausea, vomiting, diarrhea, consistency and numbers of stools per day)
- Patient's quality of life

4.4. Effect of dysbiosis correction on patient clinical status

- Rate of *C. difficile* and other resistant bacteria until the hospitalization for consolidation chemotherapy
- Infectious-related mortality during 1 year post inclusion
- Infectious-related morbidity: fever, gastrointestinal tract events as diarrhea (severity and frequence), rates and recurrence, abdominal pain, bloating after AFMT and until 6 months after inclusion.
- Duration of hospitalization, duration, dose or change variations of systemic antibiotherapy after AFMT until 6 months after patient's inclusion
- Impact on early mortality and on chemosensitivity by measure of remission rate (partial or complete) until 1 year after patient's inclusion
- Impact on global survival and disease-free survival at 70 days, 6 months and 12 months after patient's inclusion

4.5. Measurement of AFMT safety

Tolerance of AFMT by monitoring safety during the transplantation period and until the next hospitalization: type and rate of adverse effects, with documentation on severity, relationship/ imputability of adverse experience to AFMT. An adverse reaction is any unfavourable or unintended sign, symptom, or disease temporally associated with AFMT, whether or not considered linked to the procedure. A monitoring of adverse events (AE) and serious adverse events (SAE) will be performed during 12 months after patient's inclusion.

All documentation of **MaaT Pharma** is PROPRIETARY information and may not be forwarded to third parties without prior written consent from **MaaT Pharma.** Reproductions, either in part or in whole, may not be published or copied in any manner, without the explicit written consent of **MaaT Pharma**

4.6. Measurement of AFMT feasibility and acceptability

The AFMT feasibility and acceptability will be evaluated by the physician and the patient on a visual analogic scale (**Appendix 1**). Before and after chemotherapy clinical tolerance will be monitored in a logbook evaluating every day mood and quality of life, digestive symptoms (appetite, nausea, bloating, discomfort, pain), transit and stool characteristics using Bristol stool scale. The logbook will be given to the patient during study admission and carefully completed until the end of the study (**Appendix 2**). A health questionnaire EQ-5D-5L will be given to the patient at 4 timepoints (D0, D29, D40, and D70 see below) to evaluate more precisely the impact of AFMT on the quality of life.

5. SELECTION OF POPULATION FOR THE STUDY

5.1. Inclusion criteria

- Patients ≥ 18 and ≤ 75 years old with *de novo* diagnosis of AML or HR MDS for whom intensive induction chemotherapy is anticipated within 10 days after admissionPatients willing to donate stool samples and to follow protocol recommendations
- Signature of informed and written consent

5.2. Exclusion criteria

- Acute promyelocytic leukemia (AML-M3)
- Known allergy or intolerance to trehalose or maltodextrin
- Pregnancy: positive urinary or blood test in female of childbearing potential
- Severe disease with a life expectancy < 3 months
- Other on-going interventional protocol that might interfere with the study
- Non eligibility for collection of autologous stools upon admission:
  - Patients refusing to consent
  - Antibiotherapy at the time of study inclusion ≥ 4 days
  - Concomitant or previous diagnosis of a significant inflammatory bowel disease (UC, CD) or other progressive digestive disease requesting treatment or further medical exploration

All documentation of **MaaT Pharma** is PROPRIETARY information and may not be forwarded to third parties without prior written consent from **MaaT Pharma.** Reproductions, either in part or in whole, may not be published or copied in any manner, without the explicit written consent of **MaaT Pharma**

- Presence of severe colitis of any etiology at the time of admission or severe digestive disorders (acute or chronic diarrhea) within 3 months preceding inclusion
- Presence of blood in faeces collected at the time of inclusion
- Patient getting a recent colonoscopy (within 3 months preceding inclusion)

- Detection of MDRB, pathogenic bacteria, parasites, norovirus and/or rotavirus during screening of autologous stool collected immediately after the inclusion visit
- Non eligibility for inoculum transplantation: persistent mucositis, colitis, or haemorrhoids, presence of blood in more than 1 patient's faeces out of 3 the week preceding the transplantation
- Non feasibility of inoculum procedure: patient refusal; technical or biological mismatch of the inoculum
- Absence of effective contraceptive method for female of childbearing potential
- Lactation
- Inability to give an informed consent

6. STUDY DESIGN

6.1. Type of study

Single-arm multicenter prospective interventional trial in hospitalized patients (Phase I/II).

6.2. Study duration

Total duration of the study: 24 months

Inclusion period: Maximum 12 months (estimation)

The inclusion period will continue until the recruitment of 20 transplanted (AFMT) patients.

Participation period for a patient: Main period ≈ 70 days

Follow-up period: 1 year

6.3. Number of patients included in the study- Recruitment potential

Number of AML/ HR MDS patients: 20

All documentation of **MaaT Pharma** is PROPRIETARY information and may not be forwarded to third parties without prior written consent from **MaaT Pharma.** Reproductions, either in part or in whole, may not be published or copied in any manner, without the explicit written consent of **MaaT Pharma**

Recruitment of patients is estimated around 1 per month and per centre.

6.4. Experimental plan and practical procedures

This study aims to assess the feasibility and tolerance of AFMT in the setting of intensive chemotherapy delivered for the initial treatment of AML / HR MDS and the antibiotherapy given during aplasia

6.4.1. Study flow chart

Figure 1: Study flow chart

All documentation of **MaaT Pharma** is PROPRIETARY information and may not be forwarded to third parties without prior written consent from **MaaT Pharma.** Reproductions, either in part or in whole, may not be published or copied in any manner, without the explicit written consent of **MaaT Pharma**

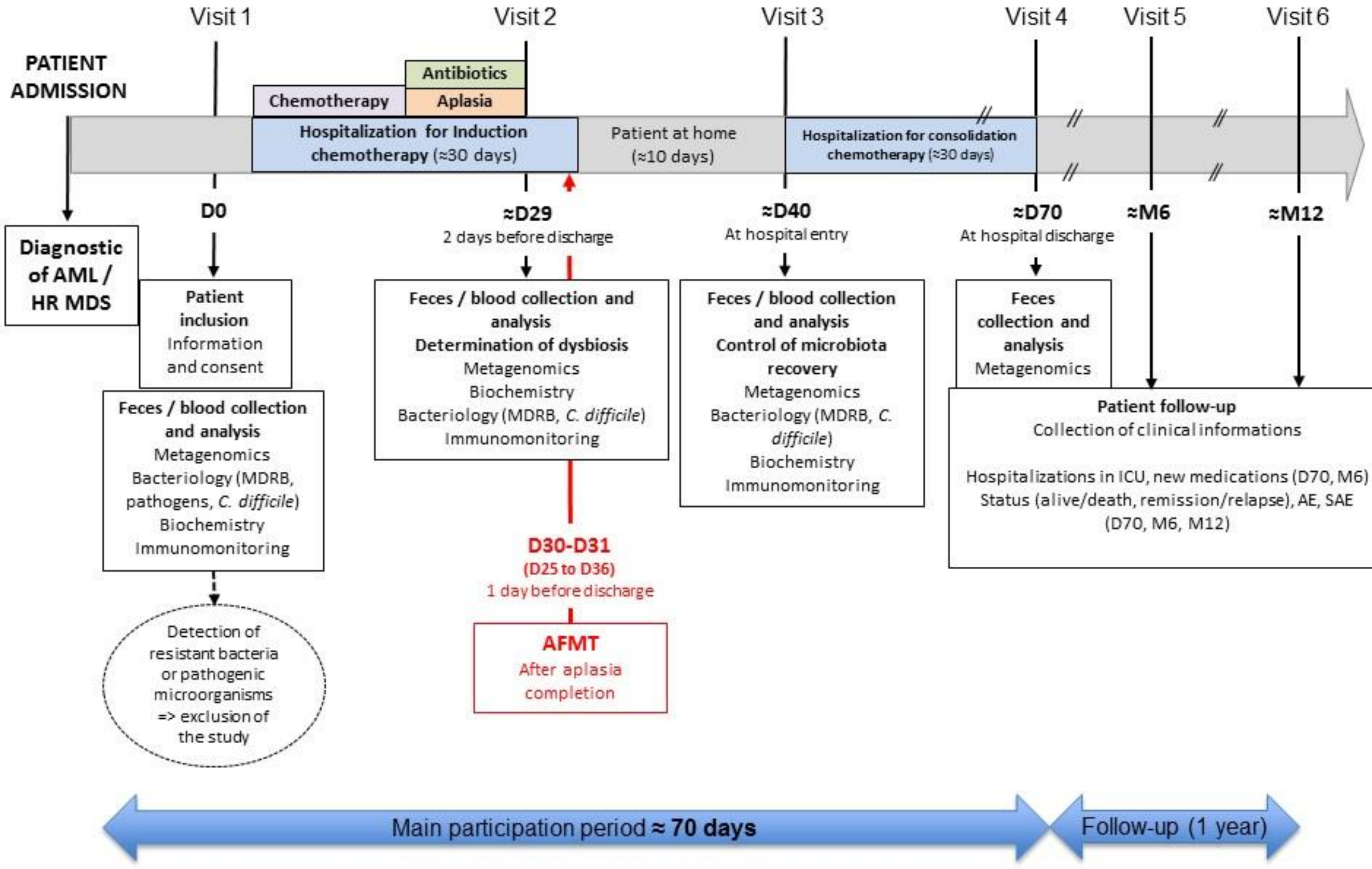

'harma. Reproductions, either in part or in

6.4.2. Conduct of the study

Day 0- Visit 1- Inclusion

Patient diagnosed with AML/ HR MDS will be asked to participate in this study by the investigating physician during his hospitalization for the treatment of AML/ HR MDS or during a consultation at day hospital before hospitalization. Prior to the initiation of any intensive chemotherapy and any antibiotherapy, the patient will be examined during a consultation by the physician to ensure that the patient fulfils inclusion and non-exclusion criteria (with the help of a questionnaire in **Appendix 3**). The patient will be enrolled in the study after receiving oral and written information and giving his consent (**Appendix 4**). Clinical and biological tests (cf table 2) will be performed as part as the normal patient care. Moreover, several microbiological analyses, selected according to ANSM recommendations and required for the study will be performed on the patient's faeces, collected with the help of a caregiver in special containers and following procedures provided by the sponsor. Collection of faeces and blood will be done as soon and as simultaneously as possible after patient's inclusion and before any chemotherapy. If needed, faeces can be collected at patient's home, before hospitalization. In that case, special containers will be provided and procedure will be explained to the patient during consultation. An user manual will also be provided. Blood sample will be collected at hospital admission.

All analyses performed on faeces and blood are:

- Research of MDRB (Methicillin-resistant *Staphylococcus aureus* (MRSA), Vancomycin- and glycopeptid-resistant enterococci (VRE, GRE), Extended-spectrum-β-lactamase (ESBLs) producing Gram-negative bacteria, carbapenemase-producing bacteria)
- Research of pathogenic bacteria (*Campylobacter sp*, *Listeria sp*., *Salmonella sp*., *Shigella sp*., *Vibrio sp*., *Yersinia sp*., Toxin+ *C. difficile*)
- Research of parasites (Strongyloides stercoralis, Cryptosporidium, Cyclospora, Isospora, Entamoeba histolytica, Giardia intestinalis, Microsporidia, Dientamoeba fragilis, Blastocystis fragilis).

Microbiological analysis will be performed in BIOFORTIS SAS according to standard operating procedures (SOP) of the laboratory. Detection of MDRB, *C. difficile*, pathogenic bacteria, parasites, norovirus and/or rotavirus in the stool collected during the inclusion visit is an exclusion criterion of the study.

All documentation of **MaaT Pharma** is PROPRIETARY information and may not be forwarded to third parties without prior written consent from **MaaT Pharma.** Reproductions, either in part or in whole, may not be published or copied in any manner, without the explicit written consent of **MaaT Pharma**

- Biochemical analysis (calprotectin, neopterin, zonulin and secretory Immunoglobulin A (IgA) assay on faeces) will be performed by BIOFORTIS SAS.
- Monitoring of blood immune cells will be performed by BIOFORTIS SAS.
- Metagenomic analyses will be performed by a state of the art sequencing company.
- Other analyses done under the responsibility of the clinician in accordance with the "Arrêté du 4 novembre" (82) will allow defining patient's status without restricting the indication of AFMT.
- A sample of faeces will be conditioned and stored in Accinov for future AFMT on the patient.

The patient will be given the logbook and the EQ-5D-5L questionnaire to evaluate its gastrointestinal symptoms and its quality of life.
The patient is next hospitalized for the beginning of his treatment (induction chemotherapy) and will be clinically and biologically monitored according to the standard procedures of the hematology department. During this period, results from microbiological screenings will be delivered to the physician who will decide if the patient is eligible to AFMT.

D29 (between D24 and D34, depending on patient's condition) – Visit 2 – Faeces analysis for dysbiosis characterization

After aplasia completion ,faeces and blood will be collected by caregivers within 3 days before antibiotics discontinuation (around Day 29) (**Procedures will be provided by BIOFORTIS SAS**) for biochemical assay, bacteriology (detection of MDRB and *C. difficile*), determination of dysbiosis using metagenomic analysis and analysis of blood immune cells. Detection of MDRB and/or *C. difficile* will not constitute a criterion for exclusion but will allow the measure of the potential impact of chemotherapy and antibiotherapy on the emergence of these bacteria. The patient will fullfil the EQ-5D-5L questionnaire to evaluate its gastrointestinal symptoms and its quality of life.The patient is next prepared for the following AFMT according to the procedure (**Appendix 1**).

All documentation of **MaaT Pharma** is PROPRIETARY information and may not be forwarded to third parties without prior written consent from **MaaT Pharma.** Reproductions, either in part or in whole, may not be published or copied in any manner, without the explicit written consent of **MaaT Pharma**

D30 and D31 (between D25 and D36 depending on patient's condition) - Fecal transplantation

The AFMT will be performed 24 hours after antibiotics discontinuation and after aplasia completion. The patient will receive 2 inocula of 150 mL corresponding to a total of 60g (2 x 30g) of faeces, according to the procedure (**Appendix 1**). A rectal preparation for AFMT will include 2 enemas with "Normacol lavement" the night before and the morning of the procedure, enhancing the potential for FMT to provide a "fresh start" in repopulating the colonic habitat of the patient. Indeed, the lavage will enhance FMT success by flushing out residual faeces, antibiotics, and *C. difficile* bacteria, toxins and spores (83). The rectal preparation and AFMT will not be performed in case of persistent mucositis, colitis, or haemorrhoids, that constitute exclusion criteria. A third inoculum is available in case of technical incident (rejection of the inoculum by the patient within 1 hour after transplantation). Fecal transplantation is performed by a rectal probe introduced in the patient's rectum. A tubing, ending with a rectal probe, is directly connected to allow the inoculum to reach the colon. The patient will remain in lying position for 1 hour to ensure a good transplantation and avoid any loss. Patient's vital signs (blood pressure, pulse, temperature, respiratory rate) will be monitored during all the time of the transplantation process, until the expulsion of the remaining transplant but at least 2 hours after AFMT. Monitoring will also focus on the recovery of intestinal transit, onset of abdominal pain, occurrence of fever before the patient's hospital discharge. In case of severe adverse event, the hospitalization will be extended to treat the event. In case of procedure-related SAE or SADR (Serious Adverse Drug Reaction) during the first AFMT, the second AFMT will not be done. Post-transplant clinical tolerance will be monitored with a questionnaire (**Appendix 1**) and the logbook (**Appendix 2**).

D40 (between D35 and D 45 depending on patient's condition)- Visit 3 - Hospitalization for consolidation chemotherapy

At this time, the patient will report to the physician any adverse reaction or severe event occurred since his hospital withdrawal using the logbook during admission visit. Particular attention will be given for occurrence of severe diarrhea, fever, abdominal pain, infectious events and concomittant medications. The patient will also receive a EQ-5D-5L questionnaire. Prior to the initiation of consolidation chemotherapy, faeces and blood will be collected for analysis (**Procedures will be provided by BIOFORTIS SAS** - biochemical and bacteriological

All documentation of **MaaT Pharma** is PROPRIETARY information and may not be forwarded to third parties without prior written consent from **MaaT Pharma.** Reproductions, either in part or in whole, may not be published or copied in any manner, without the explicit written consent of **MaaT Pharma**

analysis, control of the microbiota recovery by metagenomic and analysis of blood immune cells).

D70 (between D60 and D80)- Visit 4 - End of hospitalization for consolidation chemotherapy - End of main follow-up

At the end of the hospitalization for consolidation chemotherapy, patient's clinical information will be collected: status (alive, death with cause of death (COD); remission or relapse), AE, SAE, severe diarrhea, fever, abdominal pain, infectious diseases, and concomittant medications. The patient will also receive a EQ-5D-5L questionnaire. Faeces will be collected for analysis (**Procedures will be provided by BIOFORTIS SAS** – microbiota sequencing by metagenomic).

Month 6 (between M5 and M7)- Visit 5 - Follow-up patient.

During the visit, the patient will report any AE or SAE to the physician. Patient's clinical information will be collected : status (alive, death with COD; remission or relapse), number of hospitalizations in intensive care unit (ICU), concomittant medications.

Month 12 (between M11 and M13)- Visit 6 - Follow-up patient – End of the study

During the visit, the patient will report any SAE to the physician. Patient's clinical information will be collected : status (alive, death with COD; partial or complete remission or relapse).

All documentation of **MaaT Pharma** is PROPRIETARY information and may not be forwarded to third parties without prior written consent from **MaaT Pharma.** Reproductions, either in part or in whole, may not be published or copied in any manner, without the explicit written consent of **MaaT Pharma**

	<D0	D0	D0 to D30	D29	D30	D30 to D40	D40	D40 to D70	D70	M6	M12
Diagnostic of AML/ HR MDS (Cytology, cytogenetic, molecular biology, immunophenotyping results)	C										
Medical history	C										
Clinical exam (Temperature, blood pressure, heart rate, body mass index	C	C		C	C		C				
Clinical symptoms / status	C	C	C	C	C	C	C	C	C	C	C
Consent		R									
Laboratory evaluation (cf table 2)	C	C		C			C				
Collection of blood for immunomonitoring		R		R			R				
Collection of faeces for metagenomic analyses		R		R			R		R		
Collection of faeces for detection of multi-drug resistant bacteria		R		R			R				
Collection of faeces for detection of pathogenic bacteria, viruses and parasites		R									
Collection of faeces for *Clostridium difficile* detection		R		R			R				
Collection of faeces for biochemistry assay (calprotectin, neopterin, zonulin, secretory IgA)		R		R			R				
EQ-5D-5L questionnaire		R		R			R		R		
Faeces evaluation with Bristol Stool chart (logbook)		R	R	R	R	R	R	R	R		
Monitoring of AE/ SAE		R	R	R	R	R	R	R	R	R	R (only SAE)

R = exams required for the study
C= exams performed during usual patient care that will be collected for the study

Table 1: Summary of patient monitoring

All documentation of **MaaT Pharma** is PROPRIETARY information and may not be forwarded to third parties without prior written consent from **MaaT Pharma.** Reproductions, either in part or in whole, may not be published or copied in any manner, without the explicit written consent of **MaaT Pharma**

6.4.3. Management of analyses and samples

6.4.3.1. Routine analyses during patient care

During hospitalization and patient care, clinical and laboratory monitoring (standardized analyses detailed below, **table 2**) are usually done by the physician and analyzed locally in the hospital. The results of these analyses performed at D0, D29, and D40 (days of stool collection) will be collected and reported in the case report form (CRF). Laking of one or several parameters will not constitute an exclusion criterion.

MONITORING ROUTINELY DONE DURING HOSPITALIZATION	
**HEMATOLOGY**	**CLINICAL SURVEY**
**Diagnosis of AML** / HR MDS**:**<br>Cytology, cytogenetic, molecular biology, immunophenotyping results, number of blasts at inclusion<br><br>**Hematological response:**<br>Complete remission, partial remission, primary failure, chemoresistance, relapse (dates)	Temperature, blood pressure, heart rate, body mass index
**MEDICAL HISTORY**	**CLINICAL SYMPTOMS**
Age, sex, profession, habitation, birth delivery, smocking habits, antecedents of infectious diseases, previous AB or drugs influencing the microbiota (PPI) within 3 months (molecule, dose, duration, dates), previous stays in laminar air flow rooms (duration, dates)	Fever, major episodes of sepsis with associated treatments (molecules, doses, duration, dates), diarrhea (frequency and severity), vomiting, abdominal pain, signs of acute denutrition, intervention of psychiatrist for mood disorders
**THERAPEUTIC STRATEGY**	
Gut decontamination (antibacterial and/of antifungal therapy: date, dose, molecules, duration), chemotherapy schedule (molecules, doses, duration, dates), curative and prophylactic antibiotics (molecules, doses, duration, dates), laminar air flow or conventional rooms (dates), sterile food	
**LABORATORY EVALUATION**	
**BIOCHEMISTRY**	**MICROBIOLOGY**
Blood cell count<br>Coagulation profile<br>Ionogram<br>Serum glucose<br>Blood Ca^{2+}, Mg^{2+}, uric acid<br>Renal function (creatinine, clearance)<br>Liver function (AST, ALT, GGT, ALP, total and direct bilirubin)<br>Ferritin<br>Inflammation: C reactive protein<br>Nutritional status: albumin, pre-albumin<br>Protein electrophoresis	Fungi: urine, throat (yeasts and molds), galactomannan antigenemia<br><br>Virus: serology and/or PCR for EBV, CMV, HIV, HPV, HCV, HBV (82)<br><br>Bacteria: blood, urine, throat samples for bacterial culture

Table 2: List of exams usually performed during patient care

All documentation of **MaaT Pharma** is PROPRIETARY information and may not be forwarded to third parties without prior written consent from **MaaT Pharma.** Reproductions, either in part or in whole, may not be published or copied in any manner, without the explicit written consent of **MaaT Pharma**

6.4.3.2. Analyses required for the study

ADDITIONNAL TESTS/ INFORMATIONS REQUESTED FOR THE STUDY	
**FOLLOW-UP AFTER AFMT**	**MICROBIOLOGY ON FAECES**
Status at last follow-up (alive, death with cause of death; remission or relapse) until the end of the hospitalization for consolidation chemotherapy<br>Patient will report severe events (AB effects, fever…) to the physician.	Numbers of stools and stool consistency evaluated by Stool Bristol scale during hospitalization<br><br>*C. difficile* Multi-drug resistant bacteria<br>Pathogenic bacteria and parasites
**BIOCHEMISTRY ON FAECES**	**METAGENOMIC ANALYSIS**
Calprotectin, neopterin, zonulin, secretory IgA on faeces	Genetic sequencing of bacterial genes (shotgun: all metagenome sequencing)<br>Diversity index of bacterial microbiota<br>Dysbiosis characterization
**BLOOD ANALYSIS**	
Monitoring of blood immune cells	

Table 3: List of analyses requested for the study

The patients will be followed for assessment of study endpoints and recording adverse events that could happen after intervention until the end of the hospitalization for consolidation chemotherapy. All clinical severe events will be recorded in the CRF during any patient transfer to other site or service (Emergency care). Information of type, dose, duration of antibiotherapy, immunotherapy and chemotherapy will be given to explore the possible correlation between treatments, dysbiosis status and its severity. Relationship of adverse events to AFMT will be assigned by the investigator with the help of the data. All serious adverse reaction will be reported according to the regulatory procedure of adverse reaction declaration.

6.4.3.3. Management of fecal samples

All documentation of **MaaT Pharma** is PROPRIETARY information and may not be forwarded to third parties without prior written consent from **MaaT Pharma.** Reproductions, either in part or in whole, may not be published or copied in any manner, without the explicit written consent of **MaaT Pharma**

Place of collection	Hospital / Patient's home			
If faeces collection at home	The patient collects his faeces at home the day before his hospitalization and bring back the collection pot in a cooler (within 24h after collection) in order to proceed to sampling by caregivers. Faeces are kept in the collection pot and in a cooler.		Faeces collected by the patient at home in the collection pot are sent within 24h after collection to the hospital by a carrier.	
Sampling	Care giver will sample faeces for analyses with a special spoon according to the procedures provided by BIOFORTIS SAS			
	**Metagenomic analysis**	**Bacteriology**	**Biochemistry**	**For inoculum (AFMT)**
Weight of samples	3 x 1g	20- 30g	20-30g of faeces	Remains of the faeces (at least 100g)
Material	Fecal containers	Fecal container	Fecal container	Fecal container
Conditions of transport and delivery modes	- 80°C - Carrier	4°C- Carrier within 24h	4°C- Carrier within 24h	Room temperature - Carrier
Storage location and conditions	Storage - 80°C - Further analysis by sequencing platform	Immediate analysis- BIOFORTIS SAS	Storage -80°C and further analysis – BIOFORTIS SAS	Preparation of inocula for AFMT-ACCINOV

Table 4: Fecal samples management

6.4.3.4. Processing of samples

Faeces will be collected at hospital or at patient's home. Faeces collected at patient's home will be returned to the hospital by the patient or by a special carrier.
A fecal collection pot (the same provided at hospital) and a cooler will be provided to the patient with an user manual during the consultation. The patient will collect his faeces at home the day before his hospitalization and will bring back the collection pot in order to proceed to sampling by caregivers.

Faeces and blood will be sampled by caregiver. Then, the samples will be coded, anonymized, stored and sent to the central laboratory by a carrier as described in **Appendix 5**. Samples will be conserved during all study duration, in BIOFORTIS SAS and Accinov.

All documentation of **MaaT Pharma** is PROPRIETARY information and may not be forwarded to third parties without prior written consent from **MaaT Pharma.** Reproductions, either in part or in whole, may not be published or copied in any manner, without the explicit written consent of **MaaT Pharma**

According to our central lab procedures, the faeces conservation at room temperature for 24h before sampling and a delay of maximum 48h between faeces collection and microbiological analysis are acceptable.
For the inoculum preparation, a delay of maximum 72h at room temperature between faeces collection and inoculum process has been validated, as described in the IMPD.

In case of patient's request, biological resources will be destroyed according to internal procedures of each centre.
Processing of fecal samples will be as follow (cf **Appendix 6** for analysis details):
- In BIOFORTIS SAS :

- Research of MDRB (Methicillin-resistant *Staphylococcus aureus* (MRSA), Vancomycin- and glycopeptid-resistant enterococci (VRE, GRE), Extended-spectrum-β-lactamase (ESBLs) producing Gram-negative bacteria, carbapenemase-producing bacteria)
- Research of pathogenic bacteria (*Campylobacter sp*, *Listeria sp.*, *Salmonella sp.*, *Shigella sp.*, *Vibrio sp.*, *Yersinia sp.*, *C. difficile*)
- Research of parasites (Strongyloides stercoralis, Cryptosporidium, Cyclospora, Isospora, Entamoeba histolytica, Giardia intestinalis, Microsporidia).
- Assay of fecal calprotectin, neopterin, zonulin and secretory IgA
- Storage of metagenomic samples at -80°C. All samples will be sent to a sequencing platform for metagenomic sequencing for interim analysis as per DSMB charter and final analysis at the end of the study.

- In Accinov: preparation of inocula for AFMT

Processing of blood samples (2 x 9mL tubes) in BIOFORTIS SAS will be as follow:

- Extraction of peripheral blood mononuclear cells and storage in liquid nitrogen until analysis by flow cytometry at the end of the study
- Extraction of plasma and storage in a -80°C freezer until analysis by biochemistry multiplex assays at the end of the study or during the intermediate analysis

All documentation of **MaaT Pharma** is PROPRIETARY information and may not be forwarded to third parties without prior written consent from **MaaT Pharma.** Reproductions, either in part or in whole, may not be published or copied in any manner, without the explicit written consent of **MaaT Pharma**

7. STATISTICAL CONSIDERATIONS

7.1. Main endpoints

7.1.1. Evaluation of AFMT efficacy in dysbiosis correction

The microbiota diversity will be measured by the Simpson diversity index (S). S will be calculated at the three main times D0, D30 and D40 corresponding respectively to baseline (Sb, before antibiotherapy and chemotherapy), post-treatment (Sd, dysbiosis maximum deterioration post-antibiotherapy) and final value post-AFMT (Sf). Our hypothesis is that after an expected deterioration due to chemotherapy, the microbiota diversity should return to its baseline Sb after AFMT (thus Sf/Sb = 1). To simplify the models, we related diversity at deterioration and final values to their ratio on baseline. In what follows, Sd and Sf denote the dimensionless ratios Sd/Sb and Sf/Sb respectively.

As Simpson index constitutes a particular way at measuring microbiota, other indexes of diversity (Shannon) and germs repartition (Bray-Curtis, Sorensen, Pearson, or Spearman indexes) will be calculated.

Initial Power sample

By assuming repeated measurement between Sf and the two previous S values, we assume that the correlation R (Sd, Sf) is at least R=0.5. Without an accurate estimate of the standard deviation of Sd and Sf, we will consider in this first-in-man trial that a change observed as at least half of its standard deviation should be considered as clinically meaningful as defined by Cohen's rules (84). In these conditions, a sample size of at least 30 patients should provide a power of 0.875 to detect a difference of a clinically relevant difference $\Delta=|Sf-Sd|$, at a one-tailed significance level of 0.05.

7.1.2. Evaluation of AFMT efficacy in MDRB eradication

Based on bacterial culture and analysis of resistance gene expression by metagenomics, we will evaluate the prevalence and the quantity of MDRB before (D0), after chemotherapy/antibiotherapy (D29) and after AFMT (D40).

Prevalence of MDRB will be compared between D40 and D29 to evaluate AFMT efficacy in MDRB eradication.

All documentation of **MaaT Pharma** is PROPRIETARY information and may not be forwarded to third parties without prior written consent from **MaaT Pharma.** Reproductions, either in part or in whole, may not be published or copied in any manner, without the explicit written consent of **MaaT Pharma**

Further to recruitment difficulties, the company would limit the number of cases. The following table provides the sample size needed for some beta values (from 0.1 until 0.25) and for some effect size (defined as the standardized mean difference). It is apparent that small values of effect sizes need at least 44 patients (for effect size=0.4). However, when the effect size is at least 0.6 and with a beta risk of at least 0.20, 20 patients should constitute an acceptable size.

Effect Size	Power=	0.1	0.15	0.20	0.25
0.4		66.00	57.00	49.00	44.00
0.5		43.00	36.00	32.00	28.00
0.6		30.00	25.00	22.00	20.00
0.7		22.00	19.00	16.00	15.00
0.8		17.00	15.00	13.00	11.00
0.9		13.00	12.00	10.00	10.00
1		11.00	10.00	9.00	8.00

We conclude from this table that a sample of 20 patients might constitute an acceptable sample size for this pilot study. If necessary, an exploratory analysis can be conducted from n=12 patients to evaluate the level of the standardized mean difference.

<u>Related Power sample</u>

For this co-primary endpoint, there are no consistant epidemiologic data estimating the MDRB prevalence after induction treatment in AML/ HR MDS patients. The power for MDRB eradication based on 20 patients (calculated for the primary endpoint) is as follows:

**Prevalence after AFMT**	**Prevalence before AFMT**		
	**0.15**	**0.175**	**0.2**
**0.01**	0.557	0.649	0.729
**0.02**	0.440	0.535	0.624
**0.03**	0.352	0.445	0.535
**0.04**	0.284	0.370	0.458

All documentation of **MaaT Pharma** is PROPRIETARY information and may not be forwarded to third parties without prior written consent from **MaaT Pharma.** Reproductions, either in part or in whole, may not be published or copied in any manner, without the explicit written consent of **MaaT Pharma**

**0.05**	0.230	0.308	0.391

It is apparent that 20 patients do not provide enough power in the assumption where the difference between before and after prevalence is small. A maximum power is expected when the before and after prevalence are 0.2 and .01, respectively.

Conclusion,

A sample size of 20 patients does not reduce the interest of the study, for the main endpoint (change of dysbiosis), however, the power of the secondary endpoints (including MDRB) will inevitably be reduced. The exploratory feature of this study does allow this relaxed value, and alpha value can be increased (or one-tailed tests should be used) to increase power.

7.2. Secondary endpoints

7.2.1. Definition of a dysbiosis signature

The current knowledge of dysbiosis measurement remains very limited, so far. The main measurement is based on diversity itself measured by Simpson Index, which constitutes today the most usual measure, although probably limited to a particular aspect of the microbiota dispersion. Other parameters of diversity exist (in particular, the Shannon Index), but other aspects of the microbiota will be investigated. A first generalization of diversity will be co-abundance, defined as the probability of co-existence of two species. This calculation generalizes the diversity index, restrained to the co-existence of the same species. Another generalization is the Utility function associated with each species and characterized by either a linear utility or a utility area (included between two thresholds). In particular, the calculation of these parameters becomes complex when the number of species increases, thus one of the important objectives of the statistical analysis based on these data will be to determine the optimal level of species hierarchy Tree, allowing a sufficient precision. These endpoints, which are exploratory, will be defined with accuracy in the Statistical Analysis Plan (SAP). In what follows, we call them Alternative Dysbiosis (AD) measurements

All documentation of **MaaT Pharma** is PROPRIETARY information and may not be forwarded to third parties without prior written consent from **MaaT Pharma.** Reproductions, either in part or in whole, may not be published or copied in any manner, without the explicit written consent of **MaaT Pharma**

7.2.2. Effect of dysbiosis correction on patient clinical status

Previous researches provided some evidence of the association between the observed disorder or disturbance of the normal microbiota and the patient health status, infectious and digestive status. In the following, we call these variables Health Status Related HSR variables. In particular, one of them is the MDRB measured at each visit of the trial. For simplification of description of the following development, we keep the general term HSR to represent any health-status related variable measured, although the MDRB reduction will constitute the most important endpoint among any possible HSR.

7.2.3. Safety variables

7.2.3.1. Adverse events

Each AE will be coded to a "preferred term" and associated "primary system-organ class" according to an established and validated adverse reaction dictionary (MedDRA) before the treatment code is broken. The AE endpoint will be the number of subjects experiencing at least one event, regardless of relationship of the event to the study drug.

Adverse events will be categorized into three categories:

- The treatment emergent adverse event (TEAE),
- The pre-AFMT adverse event,
- The post-AFMT adverse event.

Definitions are given below.

Treatment-Emergent Adverse Events (TEAEs) will be defined as events occurring on or after the first study drug intake. Additionally, events present before the first study drug intake, but worsening or becoming serious under treatment will be considered as TEAEs. Although every effort will be made to establish the onset date and time, events with missing onset date will be considered as TEAEs.

Events occurring, worsening or becoming serious before the first study drug intake will be considered as pre-AFMT adverse events and events occurring, worsening or becoming serious 1 day or more after the last study AFMT will be considered as post-treatment adverse events.

All documentation of **MaaT Pharma** is PROPRIETARY information and may not be forwarded to third parties without prior written consent from **MaaT Pharma.** Reproductions, either in part or in whole, may not be published or copied in any manner, without the explicit written consent of **MaaT Pharma**

7.2.3.2. Laboratory data

Standard clinical laboratory tests as described in Section 6.4.2. will be performed by the central laboratory BIOFORTIS SAS.

7.2.3.3. Vital signs

Vital signs and physical examination will include blood pressure, temperature, heart rate and body mass index measurements.

7.3. Inferential aspects for criteria of efficacy

7.3.1. Co-primary endpoint: dysbiosis correction due to AFMT

The statistical distribution of Simpson index (S) will be fitted to a Normal or log-Normal distribution.

The significance of the AFMT effect will be assessed in assessing the significance of the differences Δ= |Sf – Sd| by using a paired t-test.

In case where S is distributed as a log-normal distribution, the ration Sf/Sd will be tested instead Δ= Sf – Sd, by using a log transform. The transformation Log(Sf) – Log(Sd) = Log(Sf/Sd) allows to evaluate the difference of Sf and Sd in terms of ratio (Sf/Sd).

7.3.2. Co-primary endpoint: MDRB eradication

The significance of the difference of prevalence between final (f), deterioration time (d) and baseline time (b) will be assessed a paired t-test. As for the main endpoint, Log transformed values will be used in case the MDRB is distributed according a Log-Normal distribution.

7.3.3. Secondary analyses : Association between S and health status

The assumed hypothesis is that the deterioration of the microbiota (MB) is associated with patient health status. In what follows, we consider one variable H in particular among the candidate HSR set, with Hb, Hd and Hf designating the three values measured at baseline, deterioration and final times, respectively. Let us consider the following model:

Hi = k_0 + k_b.Hi-1 + k_c.Si + η.center   for i=b,d,t [1]

All documentation of **MaaT Pharma** is PROPRIETARY information and may not be forwarded to third parties without prior written consent from **MaaT Pharma.** Reproductions, either in part or in whole, may not be published or copied in any manner, without the explicit written consent of **MaaT Pharma**

This model is a mixed model by considering the 3 measurements correlated within the patient random factor. The estimates are k0, kb, kc, and the standard deviation η. This model assumes the instantaneous association between the MB diversity S and the health status, and tests the existence of an association between H and S, however, without possibility to test either relationship H→S or S→H.

However, this model can be tested in two different ways:

Hi+1 = k0 + kb.Hi + kc.Si + η.center where for i=b,d,t [2]

In this model [2], health status (HS) at time (i+1) is assumed dependent on MB diversity at time i. This model will test the effect of MB diversity as a directional effect of lag=1 on HS, in other words, MB status affects HS with some delay, such result providing some evidence of a causal effect of MB on HS.

Another formulation should be

Si = k0 + kb.Si-1 + kc.Hi-1 + η.center where for i=b,d,t [3]

In this model [3], health status at time (i) is assumed to affect MB diversity at time i+1. This model will test the effect of HS effect as a directional effect of lag=1 on MB diversity. In other words, HS status affects MB with some delay, such result providing some evidence of a causal effect of HS → MB.

Free survival analysis

Global survival and free survival disease at day 70 (+/- 10 days) and 12 months after inclusion will be analyzed by Kaplan-Meyer analysis and other survival analysis for comparison: log-rank and cox proportional hazard regression models. These analyses will be described in detail in the Statistical Analysis Plan.

7.4. Interim analyses

7.4.1. Interim analysis

An interim analysis will be performed after the inclusion of 9 to 12 patients and will analyze data collected until the main follow-up completion (70 days after patient's inclusion).

All documentation of **MaaT Pharma** is PROPRIETARY information and may not be forwarded to third parties without prior written consent from **MaaT Pharma.** Reproductions, either in part or in whole, may not be published or copied in any manner, without the explicit written consent of **MaaT Pharma**

In this study, the measurement of clinical relevance is based on the standardized mean difference or cohen effect size. Although a cut-off value of Standard Mean Deviation SMD >0.5 is widely admitted for controlled trials, the observed standard deviation should be larger than expected and the SMD should underestimate the clinical relevance. Similarly the correlation R (St,Sf) is unknown and may strongly impact the power of the study. In these conditions, interim analyses will aim at an estimate of R (St, Sf) and the standard deviation, and may help to reconsider the sample size, should these values be different from the assumed values when calculating the power. The interim analysis will be conducted accordingly to the rules described in DSMB Charter.

7.4.2. Futility analysis

A one-stage futility stopping will be based on conditional power (CP), the probability to detect a significant result at the end of the study, given the results observed at an intermediate time. This analysis is planned after a possible adaptation of sample size. CP will be estimated through B-values (85, 86).

This analysis will be carried out when at least 40% of the planned sample are available, and futility threshold will be CPmin= 0.10 involving a slight increase of type 2 error (β= 0.1/0.9 = 0.111-0.10 ≅ 0.01) (87). None of these interim analyses require a type-I adjustment. Further details will be described in the Statistical Analysis Plan (SAP) as appropriate.

7.5. Individual patient Meta-analysis for a controlled result of efficacy

7.5.1. Principle

This trial will assess the existence of an effect of AFMT on MB recovery, from MB deterioration caused by medication. However, no control group was provided. Thus it is not possible at this stage to separate the assumed MB recovery caused by AFMT from a natural evolution that should otherwise have been measured in a control placebo group. Another observational study [ULYSSE study] has been planned conjointly with the same patient selection and measurements. The observational study will then provide us with a control group through an individual patient data (IPD) meta-analysis.

All documentation of **MaaT Pharma** is PROPRIETARY information and may not be forwarded to third parties without prior written consent from **MaaT Pharma.** Reproductions, either in part or in whole, may not be published or copied in any manner, without the explicit written consent of **MaaT Pharma**

7.5.2. Inferential Aspects

We plan to conduct an Individual Patient Meta-analysis (IPDM) aiming at assessing the AFMT effect compared with no treatment in recovering the initial MB previously deteriorated by medication.

The inferential model will be:

$HR_i = k_0 + k_b.H_i + k_c.S_i + k_t.FMT + \eta.center$ where for i=b,d,t [4]

This model is similar to [1] except that a treatment effect (FMT) is coded 1 or 0, following that the patient has been administered AFMT (in this study) or not (in the observational study). As the decision to treat or not has not been randomized and originates from two separate studies, the comparison between baseline values of the two studies will identify variables Xi for which differences were identified on the two studies, and the used model will be adjusted for these variables as shown in the following model [5]

$HR_i = k_0 + k_b.H_i + k_c.S_i + k_t.FMT + \eta.center + \Sigma w_i X_i$ where for i=b,d,t [5]

The above mentioned models [2] and [3] assessing the direction of the effect HS-S will be conducted by adding the main treatment (FMT) effect.

7.5.3. Secondary analysis : Generalization

In the above model [5], H was elected within the set of HSR variables related with the patient health Status. There are several variables in this group called H1, ….Hn. On the other hand, S was the Simpson index considered as the most usual measurement of the MB normality. However, this index is one among the multiple measurements of the MB, for which additional research is possible (cf section 7.2.2). By considering these possible variables S1,….Sm, the search of the effect of MB on Health, consists into the multivariate effect of the vector (S1,…Sm) on the vector (H1,….Hn). This secondary analysis will be determined at the Statistical Analysis Plan level.

7.5.4. Limitations

This meta-analysis has limitations: the study effect is confounded with the treatment effect, as each study assessed the placebo (or in fact with no treatment) and autologous FMT, respectively. In that sense, it will not be possible to conclude into a treatment effect. For this reason, in this exploratory analysis, we have planned the two studies in a way that selection,

All documentation of **MaaT Pharma** is PROPRIETARY information and may not be forwarded to third parties without prior written consent from **MaaT Pharma.** Reproductions, either in part or in whole, may not be published or copied in any manner, without the explicit written consent of **MaaT Pharma**

design, and other important features should be as comparable as possible to assume an absence of study effect.

7.6. Procedures

The interim analyses will be conducted in a way to avoid any perceived bias about the results.

A SAP will be provided before starting the analysis.

Data entry will be organized as to provide a real time estimate of the existing sample.

8. DATA MANAGEMENT

- CRF management: CRF redaction and CRF filling and completion will be supported by local Clinical Research Associate (CRA) for each centre of investigation
- Data entry will be done in each centre in a common frame
- Data fusion to create a single unique database will be performed with the help of the statistician, or by a CRO before starting statistical analysis
- A final data procedure for starting statistical analysis will be written in the statistical plan included in the protocol.

9. SAFETY EVALUATION

9.1. Adverse event reporting system

9.1.1. Definitions

According to the code of Public Health (R1123-39):

- Adverse event (AE)

An AE is defined as any untoward medical occurrence in a patient or clinical investigation subject administered a pharmaceutical product and that does not necessarily have a causal relationship with this treatment. An AE can therefore be any unfavourable and unintended sign, symptom or disease temporally associated with the use of a investigational medicinal product, whether or not related to the investigational medicinal product.

- Serious adverse event (SAE)

All documentation of **MaaT Pharma** is PROPRIETARY information and may not be forwarded to third parties without prior written consent from **MaaT Pharma.** Reproductions, either in part or in whole, may not be published or copied in any manner, without the explicit written consent of **MaaT Pharma**

A SAE is defined as any untoward medical occurrence that at any dose:

- results in death,
- is life-threatening,

Note: The term “life-threatening” in the definition of “serious” refers to an event in which the subject was at risk of death at the time of the event; it does not refer to an event which hypothetically might have caused death if it were more severe.

- requires in patient hospitalization or prolongation of existing hospitalization,
- results in persistent or significant disability/incapacity,
- is a congenital anomaly and/or birth defect,
- jeopardizes the participant and may require medical or surgical treatment to prevent one of the preceding outcomes.

Medical and scientific judgment should be exercised in deciding whether expedited reporting is appropriate in other situations, such as important medical events that may not be immediately life-threatening or result in death or hospitalization but may jeopardize the subject or may require intervention to prevent one of the other outcomes listed in the definition above.

- <u>New development</u>

Any new safety data:

- which could significantly alter the benefice/risk balance evaluation of the advanced therapy product or the study
- or which could lead to consider modifications regarding the advanced therapy or the conduct of the study

For examples:

- A SAE that could be linked to the investigation and diagnostic procedures of the study and that could change the course of this study
- A significant risk for the study population (e.g. lack of efficiency of the investigational medicinal product used in the treatment of a life-threatening disease)
- Significant results from animal studies recently completed

All documentation of **MaaT Pharma** is PROPRIETARY information and may not be forwarded to third parties without prior written consent from **MaaT Pharma.** Reproductions, either in part or in whole, may not be published or copied in any manner, without the explicit written consent of **MaaT Pharma**

- Early discontinuation or temporary interruption for safety reasons of a clinical trial conducted in another country with the same investigational medicinal product
- An unexpected SAE to a non-investigational medicinal product required to conduct the study (e.g. challenge agents, emergency treatment)

The AE intensity is evaluated by the investigator according to the following classification:

- **Mild:** Awareness of signs or symptoms, but easily tolerated and are of minor irritant type causing no loss of time from normal activities. Symptoms do not require therapy or a medical evaluation; signs and symptoms are transient.
- **Moderate:** Events introduce a low level of inconvenience or concern to the participant and may interfere with daily activities, but are usually improved by simple therapeutic measures; moderate experiences may cause some interference with functioning
- **Severe:** Events interrupt the participant's normal daily activities and generally require systemic drug therapy or other treatment; they are usually incapacitating

9.1.2. Investigator responsibilities

9.1.2.1. Regulatory obligations of the investigator (Art. R. 1123-54)

All SAE should be reported immediately to the sponsor except for those SAE that the protocol (cf section 9.1.2.2.1) identifies as not needing immediate reporting.
Thus, investigators must report all events with one of the following criteria:
- Death
- Impairment of vital prognosis
- Require or extend hospitalization
- Long-lasting damages
- Congenital abnormalities/ birth defect
- Any AE judged as clinically significant

All documentation of **MaaT Pharma** is PROPRIETARY information and may not be forwarded to third parties without prior written consent from **MaaT Pharma.** Reproductions, either in part or in whole, may not be published or copied in any manner, without the explicit written consent of **MaaT Pharma**

9.1.2.2. Protocol specificities

9.1.2.2.1. Events that do not require an immediate report by the investigator

In the frame of this protocol, the following events will not require an immediate notification to the Sponsor and therefore will not be subject to expedited reporting to the Competent Authorities and Ethics Committees:

- Fever > 38°C occurring within 7 days of AFMT
- Abdominal pain within 7 days of AFMT
- Bloating within 7 days of AFMT
- Diarrhea within 7 days of AFMT

Moreover, a predefined list of events specifically known to be related to chemotherapy was set up. These events will not require an immediate notification to the Sponsor and therefore will not be subject to expedited reporting process.

- Neutropenia
- Thrombocytopenia
- Electrolytes deficiency
- Hypoalbuminemia
- Tumor lysis syndrome
- Hepatic cholestasis / cytolysis

9.1.2.2.2. Events that require an immediate report by the investigator

- Septic choc
- Intestinal perforation
- Death
- Extension of hospitalization

All documentation of **MaaT Pharma** is PROPRIETARY information and may not be forwarded to third parties without prior written consent from **MaaT Pharma.** Reproductions, either in part or in whole, may not be published or copied in any manner, without the explicit written consent of **MaaT Pharma**

9.1.2.3. Obligation of the Investigator regarding safety reporting

9.1.2.3.1. Adverse events

All Adverse Events regardless of seriousness or relationship to Investigational Product, spanning from the first visit planned in the Clinical Trial Protocol/signature of the informed consent form, up to the last visit planned in the protocol, are to be recorded on the corresponding page(s) included in the CRF.

Whenever possible, symptoms should be grouped as a single syndrome or diagnosis. The Investigator should specify the date of onset, intensity, action taken with respect to Investigational Product, corrective treatment/therapy given, additional investigations performed, outcome and his/her opinion as to whether there is a reasonable possibility that the Adverse Event was caused by the Investigational Product.

9.1.2.3.2. Serious adverse events

In the case of a Serious Adverse Event the Investigator must immediately:

- fill in the CRF (within 1 day) the information related to the Serious Adverse Event in the appropriate Serious Adverse Event Form and fax it immediately to the pharmacovigilance officer:

Email: odyssee@vigipharm.fr

Fax: 04 67 10 72 53

- send (preferably by fax or e-mail) the photocopy of all examinations carried out and the dates on which these examinations were performed, to the Pharmacovigilance officer:

Email: odyssee@vigipharm.fr

Fax: 04 67 10 72 53

Care should be taken to ensure that the subject's identity is protected and the subject's identifiers in the Clinical Trial are properly mentioned on any copy of source document provided to the Sponsor. For laboratory results, include the laboratory normal ranges.

All documentation of **MaaT Pharma** is PROPRIETARY information and may not be forwarded to third parties without prior written consent from **MaaT Pharma.** Reproductions, either in part or in whole, may not be published or copied in any manner, without the explicit written consent of **MaaT Pharma**

All further data updates should be recorded in the CRF as appropriate, and further documentation as well as additional information (for Lab data, concomitant Medication, subject status…) should be sent (by fax or e-mail) to the Monitoring Team within 1 day of knowledge. In addition, any effort should be made to further document each Serious Adverse Event that is fatal or life threatening within the week (7 days) following initial notification.

The Investigator must comply with any applicable site-specific requirements related to the reporting of SAEs (and in particular deaths) involving his/her subjects to the Ethics Committee/Institutional Review Board (EC/IRB) that approved the trial.

9.1.2.3.3. AE follow-up

The Investigator should take all appropriate measures to ensure the safety of the subjects, notably he/she should follow up the outcome of any Adverse Events (clinical signs, laboratory values or other, etc.) until the return to normal or consolidation of the subject's condition;

In case of any Serious Adverse Event, the subject must be followed up until clinical recovery is complete and laboratory results have returned to normal, or until progression has been stabilized. This may imply that follow-up will continue after the subject has left the Clinical Trial and that additional investigations may be requested by the Monitoring Team;

In case of any Serious Adverse Event brought to the attention of the Investigator at any time after the clinical trial and considered by him/her to be caused by the Investigational Product with a reasonable possibility, this should be reported to the Monitoring Team.

The information that must be transmitted to the sponsor using the SAE report form are:

- Patient identification
- Reporter identification
- Gravity and severity of the SAE
- Precise description of the SAE (diagnosis, symptoms, timing, actions required, results, evolution, including body site and severity) as well as the criterion (or criteria) for regarding the report as serious should be given.
- Start date (and time) of the SAE
- Stop date (and time) or duration of the SAE

All documentation of **MaaT Pharma** is PROPRIETARY information and may not be forwarded to third parties without prior written consent from **MaaT Pharma.** Reproductions, either in part or in whole, may not be published or copied in any manner, without the explicit written consent of **MaaT Pharma**

- Ongoing diseases or patient medical history

-Treatments received by the patient (advanced therapy medicinal product, concomitant treatment)

- A causal relationship between SAE and the investigational medicinal product, any concomitant medication, procedures or specific exams of the study

- Outcome: information on recovery and any sequelae; what specific tests and/or treatment have been required and their results; for a fatal outcome, cause of death and a comment on its possible relationship to the suspected reaction should be provided. Any autopsy or other post-mortem findings should also be provided when available. Other information: anything relevant to facilitate assessment of the case, such as medical history including allergy, drug or alcohol abuse; family history; findings from special investigations.

If the investigator considers that the SAE is linked to the study or to the investigational medicinal product, he will transmit all documents that could help the promoter (medical reports, biological and further exam results…). All these documents must be anonymized. The investigator has to respond to any request for additional information from the promoter.

9.1.3. Promoter responsibilities

9.1.3.1. Analysis and report of SAE

The promoter evaluates the safety of each advanced therapy medicinal products throughout the study. He assesses the severity of all AE reported by the investigators, their causal relationship with the advanced therapy medicinal product, any concomitant medication, and the expected or unexpected nature of AE. Any suspicion of unexpected SAE or development is reported by the promoter, within the legal timeframe, to the Agence National de Sécurité du Médicament et des produits de santé (ANSM) and to the Ethics Committee (Comité de Protection des Personnes, CPP). The initial report must be made within 7 calendar days of the SAE reception in case of death or life-threatening event. The initial report must be realized within 15 calendar days of SAE reception for other situations. The monitoring report must be made on the 8 following days. Any Suspected Unexpected Severe Adverse Reaction (SUSAR) is reported electronically in the European database EudraVigilance related to adverse drug reactions established by the European Medicines Agency (EMA).

All documentation of **MaaT Pharma** is PROPRIETARY information and may not be forwarded to third parties without prior written consent from **MaaT Pharma.** Reproductions, either in part or in whole, may not be published or copied in any manner, without the explicit written consent of **MaaT Pharma**

The promoter informs all concerned investigators of any data that could adversely impact the safety of individuals implicated in biomedical research.

9.1.3.2. Analysis and report of other safety data

Any new safety data:

- which could significantly alter the benefice/risk balance evaluation of the advanced therapy product or the study
- or which could lead to consider modifications regarding the advanced therapy or the conduct of the study

The report of safety data must be done within 15 calendar days of the promoter first learnt the event. Additional relevant information should be transmitted within a further period of 8 days.

9.1.4. Data Safety Monitoring Board

A study quality control will be performed to verify:

- The ability of the centres for investigation
- The respect of the protocol and its amendment, the notification of deviations
- The quality of the case report forms and its adequation with the patient personal data
- The respect of procedures for ADR declaration

CRA of each centre will be involved according to the good clinical practice SOP of the site.

A **Data Safety Monitoring Board** (DSMB) will be instituted.

The purpose of the DSMB is to ensure that the safety of study subjects is protected while the scientific goals of the study are being met. Specifically, the DSMB is in charge of monitoring the safety of procedures for the participants and the quality of the data, as well as the appropriate termination of studies either when significant benefits or risks have been uncovered or when it appears that the clinical trial cannot be concluded successfully.

DSMB members will sign confidentiality agreements covering DSMB activities. The study sponsor will provide remuneration.

The committee will be composed of experts representative of medicine, epidemiology and clinical trial methodology. Each DSMB member will be expected to serve for the duration of the trial.

All documentation of **MaaT Pharma** is PROPRIETARY information and may not be forwarded to third parties without prior written consent from **MaaT Pharma.** Reproductions, either in part or in whole, may not be published or copied in any manner, without the explicit written consent of **MaaT Pharma**

Members of the DSMB will not participate as investigators in the study under review. By agreeing to be a board member, the member is stating that there is no conflict of interest with regard to the trial under review by the DSMB.

The responsibilities of the DSMB and its members are:

- to ensure the ongoing safety for study subjects
- to review all study documents provided by the sponsor
- to review the conduct of the study, including protocol violations
- to review data on participant recruitment
- to protect the confidentiality of the study data and the DSMB discussions
- to make recommendations to continue, modify or terminate the study
- to be the referee for the imputability of adverse reactions

Minutes from DSMB meetings will be transmitted to ANSM.

10. DIRECT ACCESS TO SOURCE DATA AND DOCUMENTS

The investigators will permit trial-related monitoring, audits and regulatory inspections by providing direct access to source data/ documents.

Any party (e.g., domestic and foreign regulatory authorities, sponsors, monitors, investigators, clinical research assistant and auditors) with direct access should take all reasonable precautions within the constraints of the applicable regulatory requirements (L.1121-3 and R.5121-13, Code of Public Health) to maintain the confidentiality of subjects' identities and sponsor's proprietary information.

All data collected by these persons during quality controls or audit will be anonymized.

11. QUALITY ASSURANCE AND QUALITY CONTROL

The sponsor is responsible for implementing and maintaining quality assurance and quality control systems with written SOPs to ensure that trials are conducted and data are generated, documented (recorded), and reported in compliance with the protocol, Good Clinical Practices, and the applicable regulatory requirements.

All documentation of **MaaT Pharma** is PROPRIETARY information and may not be forwarded to third parties without prior written consent from **MaaT Pharma.** Reproductions, either in part or in whole, may not be published or copied in any manner, without the explicit written consent of **MaaT Pharma**

The sponsor is responsible for securing agreement from all involved parties to ensure direct access to all trial- related sites, source data/ documents, and reports for the purpose of monitoring and auditing by the sponsor, and inspection by domestic and foreign regulatory authorities.
Quality control will be applied to each stage of data handling to ensure that all data are reliable and have been processed correctly.
The investigator should ensure the accuracy, completeness, legibility and timeliness of the data reported to the sponsor in the CRF and in all required reports. Any change or correction to a CRF should be dated, initialled, and explained (if necessary) and should not obscure the original entry (e.g. an audit trail should be maintained). The sponsor should provide guidance to investigators and/ or should have written procedures to assure that changes or corrections in CRF are documented and necessary.

12. ETHICS AND LEGAL CONSIDERATIONS

The promoter is defined by the law 2004-806 (August 9th, 2004).
Before starting the research, all investigators will provide to the promoter their personal signed and dated curriculum vitae, containing their registration number at the National Medical Council (CNOM) and their number RPPS (Répertoire Partagé des Professionnels de Santé).

12.1. Notification/ submission to regulatory authorities (ANSM)

Before initiating the research, the promoter should submit any required applications to the appropriate authorities (ANSM) for review, acceptance, and permission to begin the trial. The regulation of the research (L.1123-12) aims to ensure that the rights, safety and well being of trial subjects are protected and the results of clinical trials are credible. The agency plays a central role in ensuring application of good clinical practices.

12.2. Submission to ethics committee (CPP)

In accordance with the Code of Public Health (L. 1123-6), the research protocol will be submitted by the promoter to an ethics committee (CPP). The committee's opinion will be notified to the ANSM by the promoter before the start of the study.

All documentation of **MaaT Pharma** is PROPRIETARY information and may not be forwarded to third parties without prior written consent from **MaaT Pharma.** Reproductions, either in part or in whole, may not be published or copied in any manner, without the explicit written consent of **MaaT Pharma**

12.3. Amendments

A substantial amendment is defined as an amendment to the terms of the application, or to the protocol or any other supporting documentation, that is likely to affect to a significant degree:

- the safety or physical or mental integrity of the subjects of the study;
- the scientific value of the study;
- the conduct or management of the study; or
- the quality or safety of any investigational medicinal product used in the trial.

For all studies, it is the responsibility of the sponsor to determine whether an amendment is substantial and to inform the Ethics committee and the competent authority about substantial amendments to the protocol with submission of all relevant documents in support of such amendments. The sponsor may not implement such amendments without a favourable opinion and authorization.

12.4. Data protection approval

The subject's personal data and Investigator's personal data which may be included in the Sponsor database shall be treated in compliance with all applicable laws and regulations.

When archiving or processing personal data pertaining to the Investigator and/or to the subjects, the Sponsor shall take all appropriate measures to safeguard and prevent access to this data by any unauthorized third party.

12.5. Information note and patient consent

An information note explaining the project in readily accessible terms and a consent form have been written. All information will be explained to the patient during a preliminary interview before the admission and the patient will give his written consent.

All documentation of **MaaT Pharma** is PROPRIETARY information and may not be forwarded to third parties without prior written consent from **MaaT Pharma.** Reproductions, either in part or in whole, may not be published or copied in any manner, without the explicit written consent of **MaaT Pharma**

12.6. Final research report

Upon completion of the trial, the investigator should provide the sponsor with all required reports and a final research report co-authored by the investigator and the biostatistician. This report will be examined and signed by all investigators after approval. The report will promptly be sent to the promoter after the end of the research. A report must be sent to the regulatory authorities and to the ethics committee within 1 year of the end of the research. This delay should be reduced to 90 days in case of early termination of the study.

13. PROCESSING AND ARCHIVING DATA

All documents of the research (e.g. authorization letter from ANSM, opinion of the ethics committee, successive numbered versions of the protocol, correspondence with the promoter, written consents of the patients, completed and validated CRF, all specific appendix of the study, the final research report, potential audit certificates, database) have to be retained for a period of at least 15 years after completion of the trial.

14. INSURANCE

MaaT Pharma is the promoter of the study. In accordance with the law on biomedical research, MaaT Pharma has provided insurance with **CNA Insurance compagny limited** throughout the course of the study to guaranty its legal liability. (L-2004-806, Art L.1121-10 from the Code of Public Health).

15. PUBLICATION POLICY

The Investigator undertakes not to make any publication or release pertaining to the Study and/or results of the Study without the Sponsor's prior written consent, being understood that the Sponsor will not unreasonably withhold its approval.
If the Study is being conducted at multiple sites, the Sponsor agrees that, consistent with scientific standards, first presentation or publication of the results of the Study shall be made only as part of a publication of the results obtained by all sites performing the Protocol. However, if no multicenter publication has occurred within twelve (12) months of the completion

All documentation of **MaaT Pharma** is PROPRIETARY information and may not be forwarded to third parties without prior written consent from **MaaT Pharma.** Reproductions, either in part or in whole, may not be published or copied in any manner, without the explicit written consent of **MaaT Pharma**

of this Study at all sites, the Investigator shall have the right to publish or present independently the results of this Study subject to the review procedure set forth herein. The Investigator shall provide the Sponsor with a copy of any such presentation or publication derived from the Study for review and comment at least thirty (30) days in advance of any presentation or submission for publication. In addition, if requested by the Sponsor, any presentation or submission for publication shall be delayed for a limited time, not to exceed ninety (90) days, to allow for filing of a patent application or such other measures as the Sponsor deems appropriate to establish and preserve its proprietary rights. The Investigator shall not use the name(s) of the Sponsor and/or its employees in advertising or promotional material or publication without the prior written consent of the Sponsor. The Sponsor shall not use the name(s) of the Investigator and/or the collaborators in advertising or promotional material or publication without having received his/her and/or their prior written consent(s).
The sponsor has the right at any time to publish the results of the study.

All documentation of **MaaT Pharma** is PROPRIETARY information and may not be forwarded to third parties without prior written consent from **MaaT Pharma.** Reproductions, either in part or in whole, may not be published or copied in any manner, without the explicit written consent of **MaaT Pharma**

References

1. Ley RE, Peterson DA, Gordon JI. Ecological and evolutionary forces shaping microbial diversity in the human intestine. *Cell* 2006;124:837-848.
2. Dethlefsen L, McFall-Ngai M, Relman DA. An ecological and evolutionary perspective on human-microbe mutualism and disease. *Nature* 2007;449:811-818.
3. Hooper LV. Bacterial contributions to mammalian gut development. *Trends Microbiol* 2004;12:129-134.
4. Mazmanian SK, Kasper DL. The love-hate relationship between bacterial polysaccharides and the host immune system. *Nat Rev Immunol* 2006;6:849-858.
5. Frank DN, Pace NR. Gastrointestinal microbiology enters the metagenomics era. *Curr Opin Gastroenterol* 2008;24:4-10.
6. Ley RE, Hamady M, Lozupone C, et al. Evolution of mammals and their gut microbes. *Science* 2008;320:1647-1651.
7. Hooper LV, Gordon JI. Commensal host-bacterial relationships in the gut. *Science* 2001;292:1115-1118.
8. Macpherson AJ, Harris NL. Interactions between commensal intestinal bacteria and the immune system. *Nat Rev Immunol* 2004;4:478-485.
9. Woese CR, Fox GE. Phylogenetic structure of the prokaryotic domain: the primary kingdoms. *Proc Natl Acad Sci U S A* 1977;74:5088-5090.
10. Amann RI, Ludwig W, Schleifer KH. Phylogenetic identification and in situ detection of individual microbial cells without cultivation. *Microbiol Rev* 1995;59:143-169.
11. de Martel C, Ferlay J, Franceschi S, et al. Global burden of cancers attributable to infections in 2008: a review and synthetic analysis. *Lancet Oncol* 2012;13:607-615.
12. Schwabe RF, Jobin C. The microbiome and cancer. *Nat Rev Cancer* 2013;13:800-812.
13. Grivennikov SI, Greten FR, Karin M. Immunity, inflammation, and cancer. *Cell* 2010;140:883-899.
14. Lee SH, Hu LL, Gonzalez-Navajas J, et al. ERK activation drives intestinal tumorigenesis in Apc(min/+) mice. *Nat Med* 2010;16:665-670.
15. Sears CL. Enterotoxigenic Bacteroides fragilis: a rogue among symbiotes. *Clin Microbiol Rev* 2009;22:349-369, Table of Contents.
16. Wu S, Rhee KJ, Zhang M, Franco A, Sears CL. Bacteroides fragilis toxin stimulates intestinal epithelial cell shedding and gamma-secretase-dependent E-cadherin cleavage. *J Cell Sci* 2007;120:1944-1952.
17. Castellarin M, Warren RL, Freeman JD, et al. Fusobacterium nucleatum infection is prevalent in human colorectal carcinoma. *Genome Res* 2012;22:299-306.
18. Sears CL, Pardoll DM. Perspective: alpha-bugs, their microbial partners, and the link to colon cancer. *J Infect Dis* 2011;203:306-311.
19. Wallace BD, Wang H, Lane KT, et al. Alleviating cancer drug toxicity by inhibiting a bacterial enzyme. *Science* 2010;330:831-835.
20. Iida N, Dzutsev A, Stewart CA, et al. Commensal bacteria control cancer response to therapy by modulating the tumor microenvironment. *Science* 2013;342:967-970.
21. Viaud S, Saccheri F, Mignot G, et al. The intestinal microbiota modulates the anticancer immune effects of cyclophosphamide. *Science* 2013;342:971-976.
22. van Bekkum DW, Roodenburg J, Heidt PJ, van der Waaij D. Mitigation of secondary disease of allogeneic mouse radiation chimeras by modification of the intestinal microflora. *J Natl Cancer Inst* 1974;52:401-404.
23. Navari RM, Buckner CD, Clift RA, et al. Prophylaxis of infection in patients with aplastic anemia receiving allogeneic marrow transplants. *Am J Med* 1984;76:564-572.
24. Martin PJ, McDonald GB, Sanders JE, et al. Increasingly frequent diagnosis of acute gastrointestinal graft-versus-host disease after allogeneic hematopoietic cell transplantation. *Biol Blood Marrow Transplant* 2004;10:320-327.

All documentation of **MaaT Pharma** is PROPRIETARY information and may not be forwarded to third parties without prior written consent from **MaaT Pharma.** Reproductions, either in part or in whole, may not be published or copied in any manner, without the explicit written consent of **MaaT Pharma**

25. Jones JM, Wilson R, Bealmear PM. Mortality and gross pathology of secondary disease in germfree mouse radiation chimeras. *Radiat Res* 1971;45:577-588.
26. Beelen DW, Elmaagacli A, Muller KD, Hirche H, Schaefer UW. Influence of intestinal bacterial decontamination using metronidazole and ciprofloxacin or ciprofloxacin alone on the development of acute graft-versus-host disease after marrow transplantation in patients with hematologic malignancies: final results and long-term follow-up of an open-label prospective randomized trial. *Blood* 1999;93:3267-3275.
27. Vossen JM, Guiot HF, Lankester AC, et al. Complete suppression of the gut microbiome prevents acute graft-versus-host disease following allogeneic bone marrow transplantation. *PLoS One* 2014;9:e105706.
28. Perez-Simon JA, Garcia-Escobar I, Martinez J, et al. Antibiotic prophylaxis with meropenem after allogeneic stem cell transplantation. *Bone Marrow Transplant* 2004;33:183-187.
29. Storb R, Prentice RL, Buckner CD, et al. Graft-versus-host disease and survival in patients with aplastic anemia treated by marrow grafts from HLA-identical siblings. Beneficial effect of a protective environment. *N Engl J Med* 1983;308:302-307.
30. Vossen JM, Heidt PJ, van den Berg H, Gerritsen EJ, Hermans J, Dooren LJ. Prevention of infection and graft-versus-host disease by suppression of intestinal microflora in children treated with allogeneic bone marrow transplantation. *Eur J Clin Microbiol Infect Dis* 1990;9:14-23.
31. Passweg JR, Tiberghien P, Cahn JY, et al. Graft-versus-leukemia effects in T lineage and B lineage acute lymphoblastic leukemia. *Bone Marrow Transplant* 1998;21:153-158.
32. Petersen FB, Buckner CD, Clift RA, et al. Infectious complications in patients undergoing marrow transplantation: a prospective randomized study of the additional effect of decontamination and laminar air flow isolation among patients receiving prophylactic systemic antibiotics. *Scand J Infect Dis* 1987;19:559-567.
33. Russell JA, Chaudhry A, Booth K, et al. Early outcomes after allogeneic stem cell transplantation for leukemia and myelodysplasia without protective isolation: a 10-year experience. *Biol Blood Marrow Transplant* 2000;6:109-114.
34. Eriguchi Y, Uryu H, Nakamura K, et al. Reciprocal expression of enteric antimicrobial proteins in intestinal graft-versus-host disease. *Biol Blood Marrow Transplant* 2013;19:1525-1529.
35. Holler E, Butzhammer P, Schmid K, et al. Metagenomic analysis of the stool microbiome in patients receiving allogeneic stem cell transplantation: loss of diversity is associated with use of systemic antibiotics and more pronounced in gastrointestinal graft-versus-host disease. *Biol Blood Marrow Transplant* 2014;20:640-645.
36. Jenq RR, Ubeda C, Taur Y, et al. Regulation of intestinal inflammation by microbiota following allogeneic bone marrow transplantation. *J Exp Med* 2012;209:903-911.
37. Eriguchi Y, Takashima S, Oka H, et al. Graft-versus-host disease disrupts intestinal microbial ecology by inhibiting Paneth cell production of alpha-defensins. *Blood* 2012;120:223-231.
38. Heimesaat MM, Nogai A, Bereswill S, et al. MyD88/TLR9 mediated immunopathology and gut microbiota dynamics in a novel murine model of intestinal graft-versus-host disease. *Gut* 2010;59:1079-1087.
39. Taur Y, Xavier JB, Lipuma L, et al. Intestinal domination and the risk of bacteremia in patients undergoing allogeneic hematopoietic stem cell transplantation. *Clin Infect Dis* 2012;55:905-914.
40. Ubeda C, Taur Y, Jenq RR, et al. Vancomycin-resistant Enterococcus domination of intestinal microbiota is enabled by antibiotic treatment in mice and precedes bloodstream invasion in humans. *J Clin Invest* 2010;120:4332-4341.
41. Taur Y, Jenq RR, Perales MA, et al. The effects of intestinal tract bacterial diversity on mortality following allogeneic hematopoietic stem cell transplantation. *Blood* 2014;124:1174-1182.
42. Russell WR, Gratz SW, Duncan SH, et al. High-protein, reduced-carbohydrate weight-loss diets promote metabolite profiles likely to be detrimental to colonic health. *Am J Clin Nutr* 2011;93:1062-1072.
43. David LA, Maurice CF, Carmody RN, et al. Diet rapidly and reproducibly alters the human gut microbiome. *Nature* 2014;505:559-563.
44. Atarashi K, Tanoue T, Oshima K, et al. Treg induction by a rationally selected mixture of Clostridia strains from the human microbiota. *Nature* 2013;500:232-236.

All documentation of **MaaT Pharma** is PROPRIETARY information and may not be forwarded to third parties without prior written consent from **MaaT Pharma.** Reproductions, either in part or in whole, may not be published or copied in any manner, without the explicit written consent of **MaaT Pharma**

45. Morgan XC, Huttenhower C. Chapter 12: Human microbiome analysis. *PLoS Comput Biol* 2012;8:e1002808.
46. Montassier E, Gastinne T, Vangay P, et al. Chemotherapy-driven dysbiosis in the intestinal microbiome. *Aliment Pharmacol Ther* 2015;42:515-528.
47. Gerbitz A, Schultz M, Wilke A, et al. Probiotic effects on experimental graft-versus-host disease: let them eat yogurt. *Blood* 2004;103:4365-4367.
48. Aroniadis OC, Brandt LJ. Intestinal microbiota and the efficacy of fecal microbiota transplantation in gastrointestinal disease. *Gastroenterol Hepatol (N Y)* 2014;10:230-237.
49. Eiseman B, Silen W, Bascom GS, Kauvar AJ. Fecal enema as an adjunct in the treatment of pseudomembranous enterocolitis. *Surgery* 1958;44:854-859.
50. Gough E, Shaikh H, Manges AR. Systematic review of intestinal microbiota transplantation (fecal bacteriotherapy) for recurrent Clostridium difficile infection. *Clin Infect Dis* 2011;53:994-1002.
51. Kassam Z, Lee CH, Yuan Y, Hunt RH. Fecal microbiota transplantation for Clostridium difficile infection: systematic review and meta-analysis. *Am J Gastroenterol* 2013;108:500-508.
52. van Nood E, Vrieze A, Nieuwdorp M, et al. Duodenal infusion of donor feces for recurrent Clostridium difficile. *N Engl J Med* 2013;368:407-415.
53. Youngster I, Sauk J, Pindar C, et al. Fecal microbiota transplant for relapsing Clostridium difficile infection using a frozen inoculum from unrelated donors: a randomized, open-label, controlled pilot study. *Clin Infect Dis* 2014;58:1515-1522.
54. Rossen NG, MacDonald JK, de Vries EM, et al. Fecal microbiota transplantation as novel therapy in gastroenterology: A systematic review. *World J Gastroenterol* 2015;21:5359-5371.
55. Garborg K, Waagsbo B, Stallemo A, Matre J, Sundoy A. Results of faecal donor instillation therapy for recurrent Clostridium difficile-associated diarrhoea. *Scand J Infect Dis* 2010;42:857-861.
56. Bowden TA, Jr., Mansberger AR, Jr., Lykins LE. Pseudomembraneous enterocolitis: mechanism for restoring floral homeostasis. *Am Surg* 1981;47:178-183.
57. Aas J, Gessert CE, Bakken JS. Recurrent Clostridium difficile colitis: case series involving 18 patients treated with donor stool administered via a nasogastric tube. *Clin Infect Dis* 2003;36:580-585.
58. Angelberger S, Reinisch W, Makristathis A, et al. Temporal bacterial community dynamics vary among ulcerative colitis patients after fecal microbiota transplantation. *Am J Gastroenterol* 2013;108:1620-1630.
59. Kunde S, Pham A, Bonczyk S, et al. Safety, tolerability, and clinical response after fecal transplantation in children and young adults with ulcerative colitis. *J Pediatr Gastroenterol Nutr* 2013;56:597-601.
60. Kump PK, Grochenig HP, Lackner S, et al. Alteration of intestinal dysbiosis by fecal microbiota transplantation does not induce remission in patients with chronic active ulcerative colitis. *Inflamm Bowel Dis* 2013;19:2155-2165.
61. Silverman MS, Davis I, Pillai DR. Success of self-administered home fecal transplantation for chronic Clostridium difficile infection. *Clin Gastroenterol Hepatol* 2010;8:471-473.
62. Surawicz CM, Brandt LJ, Binion DG, et al. Guidelines for diagnosis, treatment, and prevention of Clostridium difficile infections. *Am J Gastroenterol* 2013;108:478-498; quiz 499.
63. Bakken JS, Borody T, Brandt LJ, et al. Treating Clostridium difficile infection with fecal microbiota transplantation. *Clin Gastroenterol Hepatol* 2011;9:1044-1049.
64. Owens DK, Lohr KN, Atkins D, et al. AHRQ series paper 5: grading the strength of a body of evidence when comparing medical interventions--agency for healthcare research and quality and the effective health-care program. *J Clin Epidemiol* 2010;63:513-523.
65. Patel NC, Griesbach CL, DiBaise JK, Orenstein R. Fecal microbiota transplant for recurrent Clostridium difficile infection: Mayo Clinic in Arizona experience. *Mayo Clin Proc* 2013;88:799-805.
66. MacConnachie AA, Fox R, Kennedy DR, Seaton RA. Faecal transplant for recurrent Clostridium difficile-associated diarrhoea: a UK case series. *QJM* 2009;102:781-784.
67. Kelly CR, Ihunnah C, Fischer M, et al. Fecal microbiota transplant for treatment of Clostridium difficile infection in immunocompromised patients. *Am J Gastroenterol* 2014;109:1065-1071.
68. Solari PR, Fairchild PG, Noa LJ, Wallace MR. Tempered enthusiasm for fecal transplant. *Clin Infect Dis* 2014;59:319.

All documentation of **MaaT Pharma** is PROPRIETARY information and may not be forwarded to third parties without prior written consent from **MaaT Pharma.** Reproductions, either in part or in whole, may not be published or copied in any manner, without the explicit written consent of **MaaT Pharma**

69. Kleger A, Schnell J, Essig A, et al. Fecal transplant in refractory Clostridium difficile colitis. *Dtsch Arztebl Int* 2013;110:108-115.
70. Quera R, Espinoza R, Estay C, Rivera D. Bacteremia as an adverse event of fecal microbiota transplantation in a patient with Crohn's disease and recurrent Clostridium difficile infection. *J Crohns Colitis* 2014;8:252-253.
71. De Leon LM, Watson JB, Kelly CR. Transient flare of ulcerative colitis after fecal microbiota transplantation for recurrent Clostridium difficile infection. *Clin Gastroenterol Hepatol* 2013;11:1036-1038.
72. Schwartz M, Gluck M, Koon S. Norovirus gastroenteritis after fecal microbiota transplantation for treatment of Clostridium difficile infection despite asymptomatic donors and lack of sick contacts. *Am J Gastroenterol* 2013;108:1367.
73. Mandalia A, Ward A, Tauxe W, Kraft CS, Dhere T. Fecal transplant is as effective and safe in immunocompromised as non-immunocompromised patients for Clostridium difficile. *Int J Colorectal Dis* 2015.
74. Di Bella S, Gouliouris T, Petrosillo N. Fecal microbiota transplantation (FMT) for Clostridium difficile infection: focus on immunocompromised patients. *J Infect Chemother* 2015;21:230-237.
75. Alang N, Kelly CR. Weight gain after fecal microbiota transplantation. *Open Forum Infect Dis* 2015;2.
76. ANSM. La transplantation de microbiote fécal et son encadrement dans les essais cliniques. 2014.
77. ANSM. La transplantation de microbiote fécal et son encadrement dans les essais cliniques. 2015.
78. Lee CH, Steiner T, Petrof EO, et al. Frozen vs Fresh Fecal Microbiota Transplantation and Clinical Resolution of Diarrhea in Patients With Recurrent Clostridium difficile Infection: A Randomized Clinical Trial. *JAMA*. 2016;315(2):142-149.
79. Hamilton MJ, Weingarden AR, Sadowsky MJ, Khoruts A. Standardized frozen preparation for transplantation of fecal microbiota for recurrent Clostridium difficile infection. *Am J Gastroenterol* 2012;107:761-767.
80. Schwintner C., Plantamura E. Dossier du Médicament Expérimental: "Inoculum microbien MAAT001 associé au système MAAT002". 2016.
81. Schwintner C., Plantamura E. Brochure investigateur "Inoculum microbien MAAT001 associé au système MAAT002". 2016
82. Vallet B. Arrêté du 4 novembre 2014 relatif au dépistage des maladies infectieuses lors des prélèvements à des fins thérapeutiques autologues prévu à l'article R. 1211-22-1 du code de la santé publique et modifiant l'arrêté du 14 mai 2010 fixant le contenu des informations permettant d'utiliser des éléments et produits du corps humain à des fins thérapeutiques. *Journal Officiel de la République Française* 2014;0263:19182.
83. Rohlke F, Stollman N. Fecal microbiota transplantation in relapsing Clostridium difficile infection. *Therap Adv Gastroenterol* 2012;5:403-420.
84. Cohen J. *Statistical power analysis for the Behavioral Sciences*; 1988.
85. Lan KK, Wittes J. The B-value: a tool for monitoring data. *Biometrics* 1988;44:579-585.
86. Lan KK, Zucker DM. Sequential monitoring of clinical trials: the role of information and Brownian motion. *Stat Med* 1993;12:753-765.
87. Proschan MA. Statistical methods for monitoring clinical trials. *J Biopharm Stat* 1999;9:599-615.

All documentation of **MaaT Pharma** is PROPRIETARY information and may not be forwarded to third parties without prior written consent from **MaaT Pharma.** Reproductions, either in part or in whole, may not be published or copied in any manner, without the explicit written consent of **MaaT Pharma**

Sample circuit

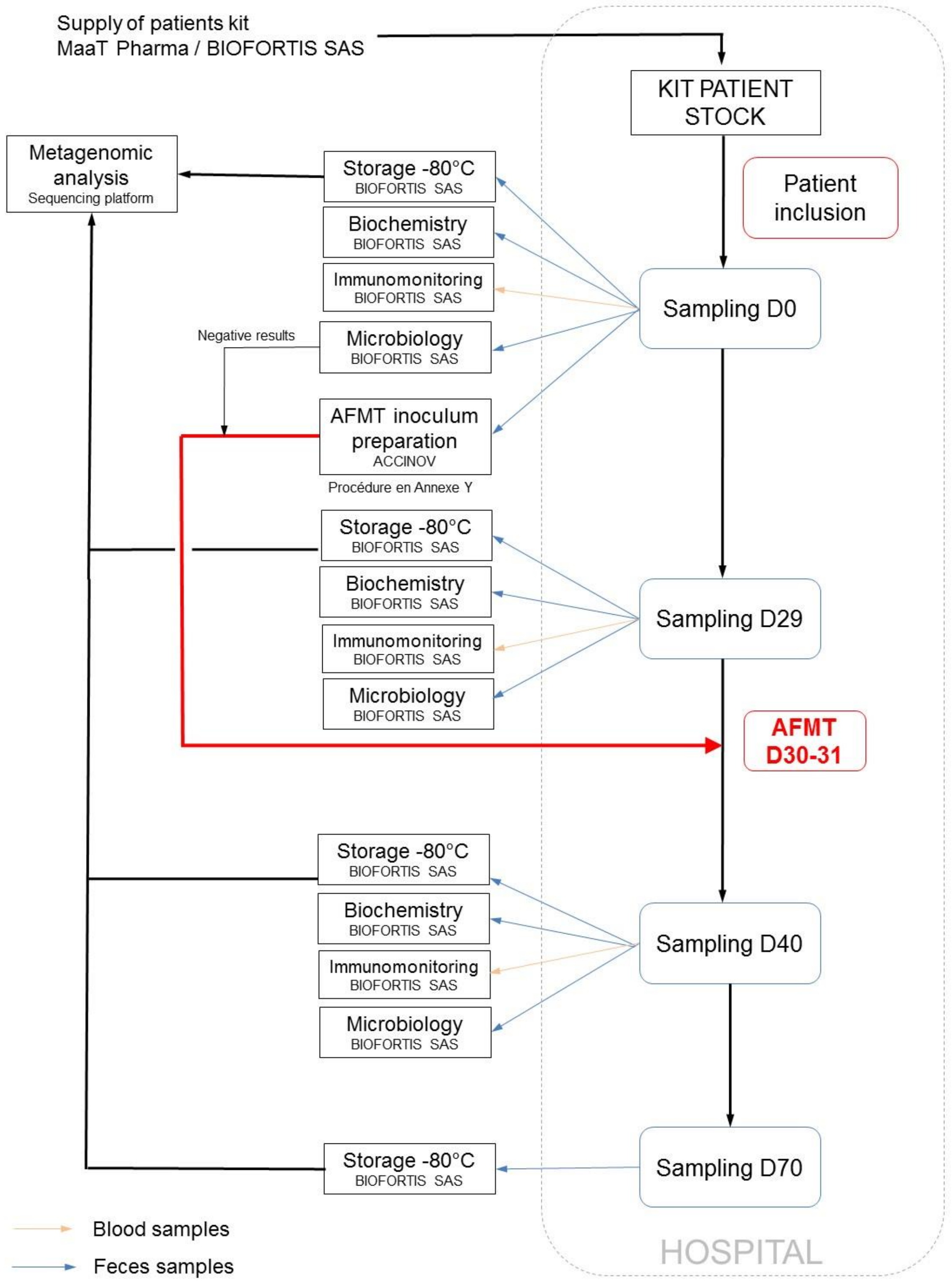

All documentation of **MaaT Pharma** is PROPRIETARY information and may not be forwarded to third parties without prior written consent from **MaaT Pharma.** Reproductions, either in part or in whole, may not be published or copied in any manner, without the explicit written consent of **MaaT Pharma**

REALISATION ET EVALUATION DE LA TRANSPLANTATION DE MICROBIOTE FECAL AUTOLOGUE

A remplir par la personne ayant procédé à la transplantation

Etiquette patient

N° identification patient:

La veille de la transplantation

La transplantation s'effectue après arrêt des antibiotiques. Le jour de l'arrêt des antibiotiques, un prélèvement de selles doit être effectué en vue de l'analyse métagénomique et biochimique pour caractérisation de la dysbiose. Un lavement avec Normacol doit être effectué le soir de l'arrêt des antibiotiques.

Contacter MaaT Pharma (et/ ou le transporteur) au _ _ . _ _ . _ _ . _ _ . _ _ pour convenir de l'envoi de l'inoculum pour le lendemain matin.

Date et heure de dernière prise des antibiotiques:

le |__|__| / |__|__| / |__|__|__|__| à |__|__| h |__|__|

Faire un lavement avec Normacol au patient.

A quelle heure le lavement a-t-il été effectué? à |__|__| h |__|__|

Le lendemain, 2 heures avant la transplantation, faire un lavement avec Normacol au patient.

A quelle heure le lavement a-t-il été effectué? à |__|__| h |__|__|

Attendre l'évacuation des selles.

Le patient a-t-il évacué les selles? Oui ☐ Non ☐

Procéder à la transplantation (page 2).

All documentation of **MaaT Pharma** is PROPRIETARY information and may not be forwarded to third parties without prior written consent from **MaaT Pharma.** Reproductions, either in part or in whole, may not be published or copied in any manner, without the explicit written consent of **MaaT Pharma**

1ère TRANSPLANTATION

Date de la 1ère transplantation: |__||__| / |__||__| / |__||__||__||__|

Nom, prénom et fonction de l'opérateur: _

Installer le patient sur son lit, allongé sur le côté gauche. Veiller à surélever les jambes et le bassin du patient.

Préparer l'inoculum : vérifier que la poche est toujours congelée. Si non, utiliser une autre poche. Si une fuite est constatée sur la poche, désinfecter la zone souillée, rebuter la poche en DASRI et prévenir le médecin investigateur.

Connecter la sonde rectale sur la tubulure de 1,50m par les connecteurs coniques mâle et femelle. Connecter la tubulure sur la poche. Si un défaut d'emballage et/ou de dispositif est constaté sur la sonde ou la tubulure, rebuter le dispositif concerné et en utiliser un autre.

Placer la poche dans le bain-marie 5 minutes à 37°C.

Important : L'inoculum à transplanter au patient doit être décongelé **5 min à 37°C** dans un bain-marie puis doit être **immédiatement** administré au patient.

Heure de début de décongélation : le |__||__| / |__||__| / |__||__||__||__| à |__||__| h |__||__|

Heure de fin de décongélation : à |__||__| h |__||__|

Une fois l'inoculum décongelé, accrocher la poche à une potence, purger l'air en faisant descendre l'inoculum dans la tubulure et insérer la sonde. Laisser l'inoculum s'écouler.

Heure de début de la transplantation : |__||__| h |__||__|

Le patient doit rester dans cette position pendant 20 minutes. Une fois que tout l'inoculum a été administré, installer le patient sur le ventre pendant 15 min, puis sur le dos pendant 15 min et enfin sur le côté droit pendant 15 min. Ces différentes positions permettent à l'inoculum de s'écouler dans le colon et permettent une meilleure transplantation.

Demander au patient d'essayer de ne pas expulser le transplant pendant au moins 4h et de rester allongé dans la mesure du possible.

All documentation of **MaaT Pharma** is PROPRIETARY information and may not be forwarded to third parties without prior written consent from **MaaT Pharma.** Reproductions, either in part or in whole, may not be published or copied in any manner, without the explicit written consent of **MaaT Pharma**

Heure de fin de la transplantation : |__||__| h |__||__|

Totalité de l'inoculum injectée : Oui ☐ Non ☐

Si non, préciser pourquoi :

_ _

_ _

_ _

Le patient a-t-il évacué l'inoculum Oui ☐ Non ☐

Si oui, préciser l'heure : |__||__| h |__||__|

Indiquer, si possible, le volume approximatif de perte |__||__||__| mL

All documentation of **MaaT Pharma** is PROPRIETARY information and may not be forwarded to third parties without prior written consent from **MaaT Pharma.** Reproductions, either in part or in whole, may not be published or copied in any manner, without the explicit written consent of **MaaT Pharma**

Evaluation de la faisabilité de la procédure de transplantation

A remplir par la personne ayant procédé à la transplantation

Entourer le chiffre correspondant

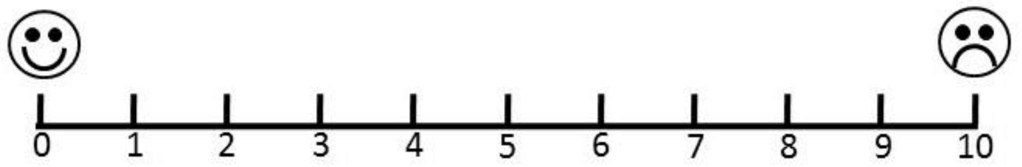

Très facile Très difficile

<u>Commentaires (difficultés, problèmes rencontrés) :</u>

<u>Signature opérateur :</u>

All documentation of **MaaT Pharma** is PROPRIETARY information and may not be forwarded to third parties without prior written consent from **MaaT Pharma.** Reproductions, either in part or in whole, may not be published or copied in any manner, without the explicit written consent of **MaaT Pharma**

A REMPLIR PAR LE PATIENT

Entourer le chiffre correspondant

<u>Comment évaluez-vous la procédure de transplantation :</u>

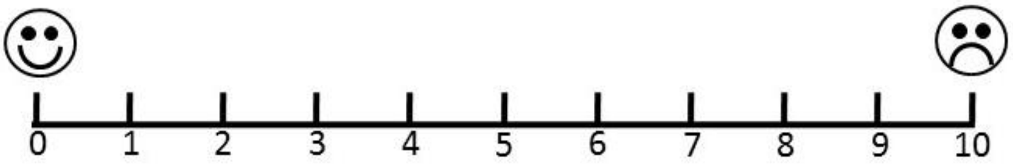

Très confortable Très inconfortable

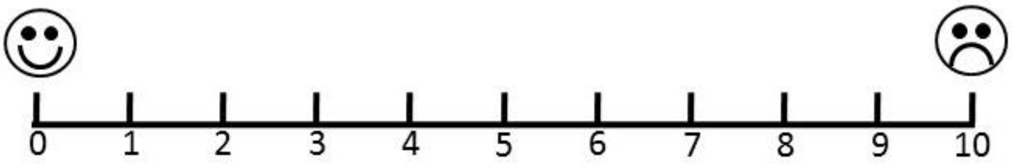

Pas de douleur Très douloureux

<u>Comment vous êtes-vous senti immédiatement après la transplantation :</u>

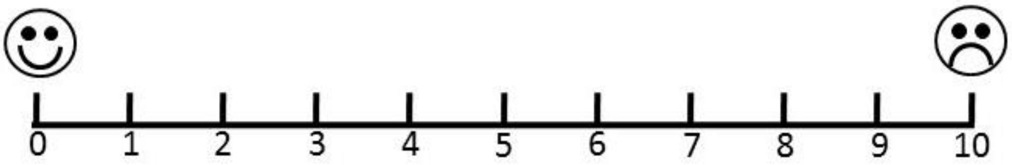

Très bien Très mal

<u>Comment vous êtes-vous senti 4 heures après la transplantation</u> :

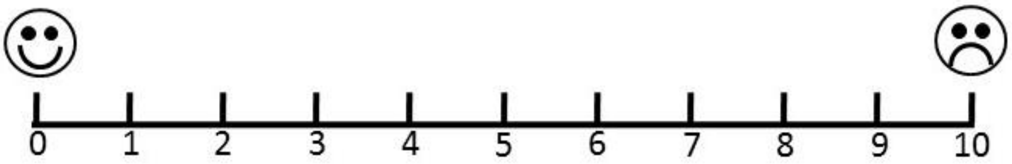

Très bien Très mal

All documentation of **MaaT Pharma** is PROPRIETARY information and may not be forwarded to third parties without prior written consent from **MaaT Pharma.** Reproductions, either in part or in whole, may not be published or copied in any manner, without the explicit written consent of **MaaT Pharma**

2ème TRANSPLANTATION

Date de la 1ère transplantation: |__||__| / |__||__| / |__||__||__||__|

Nom, prénom et fonction de l'opérateur: _

Installer le patient sur son lit, allongé sur le côté gauche. Veiller à surélever les jambes et le bassin du patient.

Préparer l'inoculum : vérifier que la poche est toujours congelée. Si non, utiliser une autre poche. Si une fuite est constatée sur la poche, désinfecter la zone souillée, rebuter la poche en DASRI et prévenir le médecin investigateur.

Connecter la sonde rectale sur la tubulure de 1,50m par les connecteurs coniques mâle et femelle. Connecter la tubulure sur la poche. Si un défaut d'emballage et/ou de dispositif est constaté sur la sonde ou la tubulure, rebuter le dispositif concerné et en utiliser un autre.

Placer la poche dans le bain-marie 5 minutes à 37°C.

Important : L'inoculum à transplanter au patient doit être décongelé **5 min à 37°C** dans un bain-marie puis doit être **immédiatement** administré au patient.

Heure de début de décongélation : le |__||__| / |__||__| / |__||__||__||__| à |__||__| h |__||__|

Heure de fin de décongélation : à |__||__| h |__||__|

Une fois l'inoculum décongelé, accrocher la poche à une potence, purger l'air en faisant descendre l'inoculum dans la tubulure et insérer la sonde. Laisser l'inoculum s'écouler.

Heure de début de la transplantation : |__||__| h |__||__|

Le patient doit rester dans cette position pendant 20 minutes. Une fois que tout l'inoculum a été administré, installer le patient sur le ventre pendant 15 min, puis sur le dos pendant 15 min et enfin sur le côté droit pendant 15 min. Ces différentes positions permettent à l'inoculum de s'écouler dans le colon et permettent une meilleure transplantation.

Demander au patient d'essayer de ne pas expulser le transplant pendant au moins 4h et de rester allongé dans la mesure du possible.

Heure de fin de la transplantation : |__||__| h |__||__|

Totalité de l'inoculum injectée : Oui ☐ Non ☐

All documentation of **MaaT Pharma** is PROPRIETARY information and may not be forwarded to third parties without prior written consent from **MaaT Pharma.** Reproductions, either in part or in whole, may not be published or copied in any manner, without the explicit written consent of **MaaT Pharma**

Si non, préciser pourquoi :

____

____

____

Le patient a-t-il évacué l'inoculum Oui ☐ Non ☐

Si oui, préciser l'heure : |__||__| h |__||__|

Indiquer, si possible, le volume approximatif de perte |__||__||__| mL

Evaluation de la faisabilité de la procédure de transplantation

A remplir par la personne ayant procédé à la transplantation

Entourer le chiffre correspondant

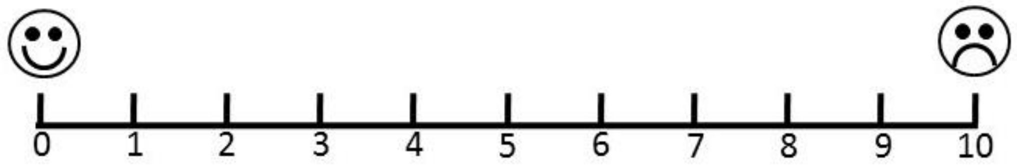

Très facile Très difficile

<u>Commentaires (difficultés, problèmes rencontrés) :</u>

____

____

____

<u>Signature opérateur</u>

A REMPLIR PAR LE PATIENT

Entourer le chiffre correspondant

All documentation of **MaaT Pharma** is PROPRIETARY information and may not be forwarded to third parties without prior written consent from **MaaT Pharma.** Reproductions, either in part or in whole, may not be published or copied in any manner, without the explicit written consent of **MaaT Pharma**

Comment évaluez-vous la procédure de transplantation :

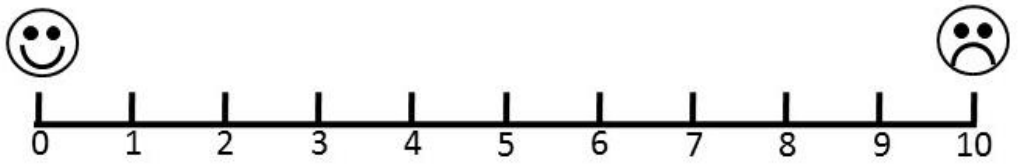

Très confortable | Très inconfortable

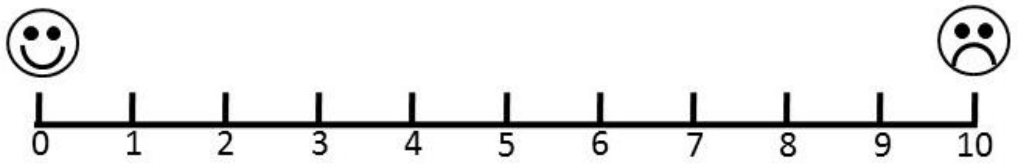

Pas de douleur | Très douloureux

Comment vous êtes-vous senti immédiatement après la transplantation :

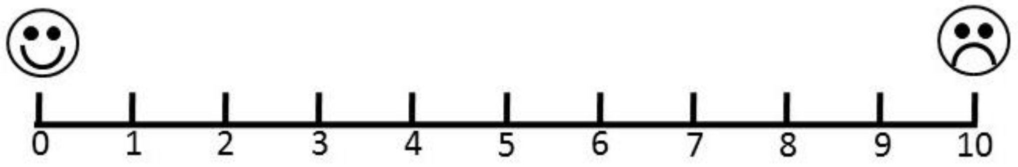

Très bien | Très mal

Comment vous êtes-vous senti 4 heures après la transplantation :

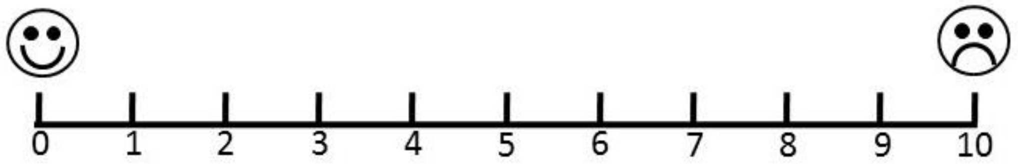

Très bien | Très mal

Comment vous êtes-vous senti le lendemain de la transplantation :

All documentation of **MaaT Pharma** is PROPRIETARY information and may not be forwarded to third parties without prior written consent from **MaaT Pharma.** Reproductions, either in part or in whole, may not be published or copied in any manner, without the explicit written consent of **MaaT Pharma**

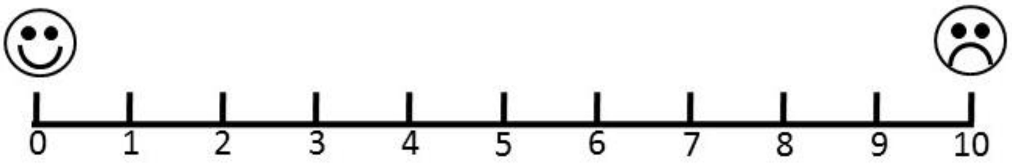

Très bien Très mal

Commentaires :

_ _

_ _

_ _

All documentation of **MaaT Pharma** is PROPRIETARY information and may not be forwarded to third parties without prior written consent from **MaaT Pharma.** Reproductions, either in part or in whole, may not be published or copied in any manner, without the explicit written consent of **MaaT Pharma**

JOURNAL DE BORD PATIENT

A remplir par le médecin :

Nom Prénom : ………………………………….

Numéro d'inclusion: …………………...

Date d'inclusion du patient : |__||__| / |__||__| / |__||__||__||__|

Etiquette Patient

INSTRUCTIONS :

Remplir ce formulaire à chaque visite (V1, V2, V3, V4, V5), votre réponse doit se rapporter aux 24 heures précédentes. Ne pas se tromper de colonne. Reporter les évènements notables dans la partie « remarques ».

Dates des prélèvements de selles :

1er prélèvement : |__||__| / |__||__| / |__||__||__||__|

2ème prélèvement : |__||__| / |__||__| / |__||__||__||__|

3ème prélèvement : |__||__| / |__||__| / |__||__||__||__|

Date de la 1ère transplantation fécale :

|__||__| / |__||__| / |__||__||__||__|

Date de la 2ème transplantation fécale :

|__||__| / |__||__| / |__||__||__||__|

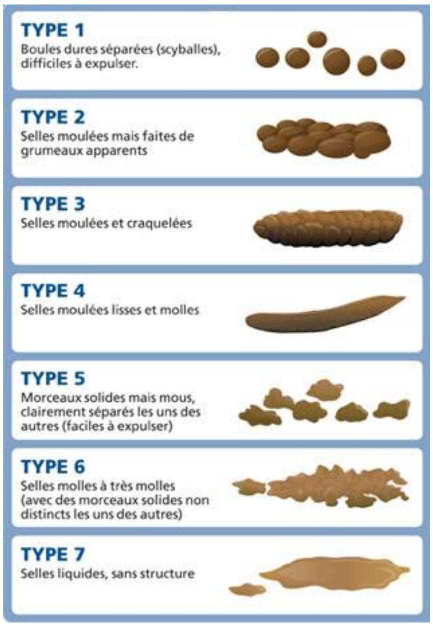

<u>Echelle de Bristol</u>

All documentation of **MaaT Pharma** is PROPRIETARY information and may not be forwarded to third parties without prior written consent from **MaaT Pharma.** Reproductions, either in part or in whole, may not be published or copied in any manner, without the explicit written consent of **MaaT Pharma**

A remplir par le patient:

**Dates**	__/__<br>V1	__/__<br>V2	__/__<br>V3	__/__<br>V4	__/__<br>V5
**Nombre de selles**					
**Consistance des selles**<br>(cf Echelle de Bristol)					
**Présence de sang dans les selles**<br>(0 non ; 1 oui)					
**Douleurs abdominales**<br>(0 aucune ; 1 légères ; 2 moyennes ; 3 fortes)					
**Nausées**<br>(0 aucunes ; 1 légères ; 2 moyennes ; 3 fortes)					
**Ballonnements**<br>(0 aucun ; 1 légers ; 2 moyens ; 3 forts)					
**Bien-être général**<br>(0 bon ; 1 moyen ; 2 médiocre ; 3 mauvais ; 4 très mauvais)					
**Appétit**<br>(0 important ; 1 moyen ; 2 léger ; 3 aucun)					
**Fièvre élevée > 38 °C**<br>(0 non ; 1 oui)					

Remarques:

_ _
_ _
_ _
_ _
_ _
_ _
_ _

All documentation of **MaaT Pharma** is PROPRIETARY information and may not be forwarded to third parties without prior written consent from **MaaT Pharma.** Reproductions, either in part or in whole, may not be published or copied in any manner, without the explicit written consent of **MaaT Pharma**

QUESTIONNAIRE VISITE D'INCLUSION

Ce document sert de support au médecin investigateur pour la visite d'inclusion.

Les réponses à ce questionnaire sont à reporter sur le CRF.

Date de naissance : |__||__| / |__||__| / |__||__||__||__

Sexe : □ **Homme** □ **Femme**

Poids : |__||__| , |__||__| **kg**

Taille : |__| , |__||__| m

Date : |__||__| / |__||__| / |__||__||__||__|

Numéro d'inclusion:

Etiquette Patient

Etes-vous né(e) par césarienne ? Oui ☐ Non ☐

<u>Avez-vous dans votre vie :</u>

Eu une maladie nécessitant un suivi médical régulier ? Oui ☐ Non ☐

Si oui, laquelle (lesquelles) ? …………………………………………………………………

Eté hospitalisé(e) ? Oui ☐ Non ☐

Si oui, pour quelle(s) indication(s) ? Quelle(s) date(s) ?…………………………………….

Eu un précédent diagnostic de cancer ou de maladie maligne (en dehors de la leucémie aiguë)? Oui ☐ Non ☐

Si oui, laquelle (lesquelles) ?…………………………………………………………………

<u>Dans les 6 derniers mois, avez-vous :</u>

Eté opéré(e) au cours d'une hospitalisation et/ou subi une anesthésie générale ?
Oui ☐ Non ☐

Si oui, pour quelle indication ? ....

All documentation of **MaaT Pharma** is PROPRIETARY information and may not be forwarded to third parties without prior written consent from **MaaT Pharma.** Reproductions, either in part or in whole, may not be published or copied in any manner, without the explicit written consent of **MaaT Pharma**

Eu une gastro-entérite fébrile (diarrhée avec fièvre) ? Oui ☐ Non ☐

Eu une endoscopie (fibroscopie, gastroscopie, coloscopie…) ?

Oui ☐ Non ☐

Pris des médicaments ? Oui ☐ Non ☐

Si oui, lesquels ? (date, durée, dose)

....

....

....

Pris des antibiotiques, des antiviraux ou des antifongiques ? Oui ☐ Non ☐

Si oui, quel traitement, dose et dates ? …………………………………………………

Pour quelle(s) indication(s) ?...

Pris des inhibiteurs de la pompe à protons (Mopral®, Inexium®, Lanzor®, Eupantol®…) ?

Oui ☐ Non ☐

Si oui, quel traitement, dose et dates ? ……………………………………………….

Pris des probiotiques ou prébiotiques ? Oui ☐ Non ☐

Si oui, quel traitement, dose et dates ? ……………………………………………….

Pour les femmes :

Etes-vous enceinte ? Oui ☐ Non ☐

Habitudes alimentaires :

Avez-vous un régime particulier ? Oui ☐ Non ☐

Si oui, lequel ? ……………………………………………………………………………..

Etes-vous végétarien ou végétalien ? Oui ☐ Non ☐

All documentation of **MaaT Pharma** is PROPRIETARY information and may not be forwarded to third parties without prior written consent from **MaaT Pharma.** Reproductions, either in part or in whole, may not be published or copied in any manner, without the explicit written consent of **MaaT Pharma**

Terrain gastro-intestinal :

Avez-vous un transit intestinal régulier ? Oui ☐ Non ☐

En moyenne, combien de selles avez-vous par jour ? ……….. selles / jour

A quel moment de la journée allez-vous à la selle habituellement ?

Matin ☐ Midi ☐ Soir ☐

Avez-vous des intolérances à certains aliments (gluten, lactose…) ? Oui ☐ Non ☐

Si oui, lesquelles ? …………………………………………………………………………..

Avez-vous :

Une maladie inflammatoire chronique de l'intestin (maladie de Crohn, rectocolite hémorragique) ? Oui ☐ Non ☐

Une maladie coeliaque ? Oui ☐ Non ☐

Un syndrome de l'intestin irritable, constipation chronique idiopathique ou de diarrhée chronique ? Oui ☐ Non ☐

Cancer colique ou polype colique ? Oui ☐ Non ☐

Etes-vous sujet aux hémorroïdes ? Oui ☐ Non ☐

Avez-vous eu une gastro-entérite (diarrhée de début aigu et de durée de moins d'une semaine) dans les 3 derniers mois ? Oui ☐ Non ☐

Avez-vous déjà eu du sang dans les selles ? Oui ☐ Non ☐

Si oui, avez-vous eu des examens complémentaires ? Oui ☐ Non ☐

Quel a été le résultat de ces examens ?...

Etes-vous allergique ou intolérant à la maltodextrine? Oui ☐ Non ☐

All documentation of **MaaT Pharma** is PROPRIETARY information and may not be forwarded to third parties without prior written consent from **MaaT Pharma.** Reproductions, either in part or in whole, may not be published or copied in any manner, without the explicit written consent of **MaaT Pharma**

Etes-vous allergique ou intolérant au tréhalose? Oui ☐ Non ☐

Autre :

Faites-vous l'objet d'une mesure de protection légale (tutelle, curatelle, sauvegarde de justice) ? Oui ☐ Non ☐

Le _ _ / _ _ / _ _

Nom, prénom, fonction de la personne ayant rempli le questionnaire:

_ _

All documentation of **MaaT Pharma** is PROPRIETARY information and may not be forwarded to third parties without prior written consent from **MaaT Pharma.** Reproductions, either in part or in whole, may not be published or copied in any manner, without the explicit written consent of **MaaT Pharma**

NOTICE D'INFORMATION DU PATIENT

Etude de la sécurité et faisabilité de la transplantation de microbiote fécal autologue pour restaurer la microdiversité intestinale et prévenir les complications de la dysbiose chez des patients atteints de leucémie aiguë myéloïde sous chimiothérapie intensive

ETUDE ODYSSEE

Cette recherche est organisée par MaaT Pharma
317 avenue Jean Jaurès
69007 LYON

Madame, Mademoiselle, Monsieur,

Le Docteur ………………………………………… (nom, prénom), exerçant à l'hôpital …………………………….. vous propose de participer à une recherche concernant votre maladie.

Dès la naissance, le corps humain est en constante interaction avec une quantité considérable de microbes, le microbiote (composé de bactéries, parasites, champignons et virus, anciennement appelé flore), avec lequel il vit en harmonie. Le microbiote au niveau de l'intestin est composé de 100 000 milliards de bactéries. Il constitue un véritable organe car les espèces bactériennes qui le composent exercent des fonctions cruciales pour notre santé comme l'élimination des substances étrangères à l'organisme (et potentiellement toxiques) ou le maintien à distance de pathogènes qui nous contaminent. Elles assurent également la dégradation des aliments ingérés pour une meilleure absorption intestinale et un métabolisme optimal. Ces milliards de bactéries jouent un rôle clef dans la maturation des défenses immunitaires. Les espèces bactériennes qui composent le microbiote intestinal diffèrent toutefois d'un individu à l'autre et la présence ou l'absence de telle ou telle bactérie semble influencer la survenue de certaines maladies ou au contraire nous protéger.
Dans certaines conditions, l'équilibre normal du microbiote intestinal peut être perturbé (dysbiose). La chimiothérapie et les antibiotiques prescrits dans le cadre du traitement de votre maladie font partie des agents pouvant perturber le microbiote intestinal.

All documentation of **MaaT Pharma** is PROPRIETARY information and may not be forwarded to third parties without prior written consent from **MaaT Pharma.** Reproductions, either in part or in whole, may not be published or copied in any manner, without the explicit written consent of **MaaT Pharma**

La transplantation fécale est une thérapie visant à restaurer l'équilibre de la flore intestinale. Cette technique consiste à réintroduire les selles (c'est-à-dire la flore ou le microbiote) d'un donneur sain dans le tube digestif d'un patient receveur en vue de rééquilibrer la flore intestinale altérée de ce dernier. Dans le cadre de l'étude, la transplantation est dite autologue : le donneur et le receveur sont la même personne, c'est-à-dire vous-même.

Cette étude permettra de mieux comprendre les effets de la restauration d'un microbiote « sain » par transplantation fécale autologue (c'est-à-dire que le donneur et le receveur sont la même personne, c'est-à-dire vous-même) *sur la possibilité d'augmenter la résistance face à la maladie et de diminuer l'incidence de certaines complications infectieuses.*

Il est important de lire attentivement cette note avant de prendre votre décision : n'hésitez pas à poser des questions et demander des explications à votre médecin. Si vous décidez de participer à cette recherche, un consentement écrit vous sera demandé.

1) Quel est le but de cette recherche ?

Cette recherche porte sur l'évaluation de la transplantation autologue de selles dans la prise en charge des patients atteints de leucémie. Cette étude est la première en France à étudier la transplantation autologue, au contraire des transplantations à partir de selles de donneurs qui sont, à ce jour, bien caractérisées. L'objectif de cette étude est d'évaluer la faisabilité et les conséquences de la transplantation fécale sur la composition de votre flore intestinale et ses effets potentiels sur les complications infectieuses qui peuvent intervenir au cours de l'évolution de votre maladie.

Pour répondre à la question posée dans cette recherche, il est prévu d'inclure 20 personnes présentant une leucémie aiguë myéloïde ou un syndrome myélodysplasique à haut risque.

2) En quoi consiste la recherche ?

Vous allez recevoir une cure de chimiothérapie et des antibiotiques tel que défini dans le cadre de votre traitement. Avant le début de cette cure, des échantillons de selles et de sang seront récoltés, conservés de façon appropriée et analysés afin d'étudier la composition microbienne de votre flore intestinale. Ces échantillons de selles pourront être collectés soit à votre domicile avec le matériel adéquat fourni, soit à l'hôpital. A la fin de votre hospitalisation (après la chimiothérapie et la prise d'antibiotiques), de nouveaux échantillons de selles et de sang seront récoltés afin d'analyser les conséquences de votre traitement sur la composition de la flore. Avant votre sortie de l'hôpital, vous recevrez 2 transplantations à partir des selles

All documentation of **MaaT Pharma** is PROPRIETARY information and may not be forwarded to third parties without prior written consent from **MaaT Pharma.** Reproductions, either in part or in whole, may not be published or copied in any manner, without the explicit written consent of **MaaT Pharma**

collectées à votre admission. Les transplantations se feront sous forme de lavement. Un volume de 150mL de suspension bactérienne préparée à partir de vos selles sera introduit dans votre tube digestif grâce à une sonde rectale. A votre réadmission à l'hôpital pour la cure suivante de chimiothérapie et à la fin de celle-ci, des échantillons de selles et de sang seront collectés afin d'évaluer le rétablissement de votre flore intestinale.

3) Quel est le calendrier de la recherche ?

La recherche durera jusqu'à 24 mois et votre participation sera de 12 mois maximum.
Si vous décidez de participer à cette étude et que vous signez et datez le formulaire de consentement éclairé durant la première consultation, votre médecin vous demandera une série d'examens qui sont ceux de la prise en charge habituelle de votre maladie que vous souhaitiez ou non participer à l'étude. Il vous fournira également un pot de collection des selles ainsi qu'une notice explicative afin de procéder au recueil de vos selles, soit à l'hôpital, soit à votre domicile. Dans le cas d'une collecte à domicile, une glacière vous sera fournie pour le transport des échantillons vers l'hôpital, le jour de votre hospitalisation. Un transporteur pourra également être prévu pour l'envoi des selles à l'hôpital.
Un examen des selles sera réalisé (analyse des gènes bactériens pour identification). Une partie des selles sera conservée en vue de la transplantation. Un journal de bord vous sera donné afin que vous puissiez enregistrer au fur et à mesure les symptômes intestinaux que vous pourriez présenter. A la fin de votre hospitalisation pour la chimiothérapie, un échantillon de selles sera récolté pour de nouvelles analyses. Les deux jours suivants, vous recevrez les transplantations fécales. Après la transplantation, vous serez surveillé(e) jusqu'au lendemain matin avant de retourner à votre domicile.
A votre retour à l'hôpital pour la cure de consolidation et à la fin de celle-ci, deux échantillons de selles seront collectés pour analyses afin d'évaluer l'efficacité de la transplantation. Vous serez ensuite pris en charge par l'hôpital dans le cadre de votre traitement.
Des échantillons de sang seront également collectés au début et à la fin de votre cure d'induction, ainsi qu'au début de la cure de consolidation. Ces échantillons seront conservés et analysés en fin d'étude.

4) Quels sont les bénéfices et les contraintes liés à votre participation ?

La dysbiose intestinale étant impliquée dans la survenue d'effets indésirables lors du traitement des leucémies ou myélodysplasies (diarrhées, infections…), nous espérons que la

All documentation of **MaaT Pharma** is PROPRIETARY information and may not be forwarded to third parties without prior written consent from **MaaT Pharma.** Reproductions, either in part or in whole, may not be published or copied in any manner, without the explicit written consent of **MaaT Pharma**

transplantation permettra d**'améliorer votre résistance à la maladie et de diminuer la survenue de complications et plus particulièrement des complications infectieuses**.

En participant à cette recherche, aucun frais supplémentaire ne vous sera demandé.
Si vous acceptez de participer, vous devrez respecter les points suivants :

- Informer le médecin de l'utilisation de tout médicament ainsi que de tout événement survenant pendant l'étude (diarrhée importante, fièvre, douleurs abdominales sévères…)
- Rapporter les documents spécifiques à la recherche (journal de bord)
- Ne pas prendre part à un autre projet de recherche sans l'accord de votre médecin

5) Quels sont les risques prévisibles de la recherche ?

Parmi les données disponibles concernant la transplantation fécale à partir de donneurs (dite hétérologue) dans d'autres maladies, aucun effet secondaire notable n'a été noté. Aucun examen complémentaire invasif, contraignant ou douloureux ne vous sera demandé au cours de l'étude.

6) Que vont devenir les prélèvements effectués pour la recherche ?

Les échantillons biologiques prélevés lors de cette étude seront vos selles et votre sang. Une partie des prélèvements sera utilisée pour faire une analyse immédiate. Une autre partie sera conservée afin de réaliser des analyses à la fin de la durée totale de l'étude.
A l'exception de la selle collectée à votre admission pour la préparation de votre médicament, et pour laquelle la réglementation nous impose de conserver des échantillons pour la production de votre médicament, les prélèvements biologiques seront conservés chez BIOFORTIS SAS et Accinov pour une durée maximale de 1 an après la fin de l'étude. Les échantillons non analysés à la fin de l'étude seront anonymisés et utilisés à des fins de recherche. Le promoteur a fait une déclaration d'activité CODECOH (Conservation D'Eléments du COrps Humain) auprès du Ministre chargé de la Recherche.
Vous avez la possibilité à tout moment de demander au médecin qui vous suit dans le cadre de la recherche la destruction de ces prélèvements biologiques ou de vous opposer à toute utilisation ultérieure.

7) Quelles sont les éventuelles alternatives médicales ?

All documentation of **MaaT Pharma** is PROPRIETARY information and may not be forwarded to third parties without prior written consent from **MaaT Pharma.** Reproductions, either in part or in whole, may not be published or copied in any manner, without the explicit written consent of **MaaT Pharma**

La transplantation proposée dans cette étude vient en adjonction au traitement habituel de la leucémie, **en vue de corriger un déséquilibre de la flore intestinale pour lequel aucun traitement n'existe actuellement**. En cas de non-participation à l'étude, le traitement de votre maladie serait identique, la transplantation mise à part.

8) Quelles sont les modalités de prise en charge médicale à la fin de votre participation ?

A la fin ou à la sortie de l'étude, le traitement de votre leucémie sera continué de manière habituelle en suivant les recommandations internationales. Votre médecin pourra décider à tout moment de l'arrêt de votre participation ; il vous en expliquera les raisons.

9) Si vous participez, que vont devenir les données recueillies pour la recherche ?

Dans le cadre de la recherche biomédicale, un traitement de vos données personnelles va être mis en œuvre et rentrera dans une étude globale sur environ 20 patients pour permettre d'analyser les résultats de la recherche au regard de l'objectif de cette dernière qui vous a été présenté.

A cette fin, les données médicales vous concernant et les données relatives à vos habitudes de vie, seront transmises anonymement au promoteur de la recherche ou aux personnes ou sociétés agissant pour son compte, en France. Ces données seront identifiées par un numéro de code et vos initiales. Ces données anonymisées pourront également être transmises aux autorités de santé françaises. Pour tout arrêt de participation sans retrait de consentement, les données recueillies précédemment à cet arrêt seront utilisées sauf si vous ne le souhaitez pas.

10) Comment cette recherche est-elle encadrée ?

MaaT Pharma a souscrit une assurance (Police N°FNAMT 101 917 66) garantissant sa responsabilité civile et celle de tout intervenant auprès de la compagnie **CNA Insurance Company limited** dont l'adresse est 37 rue de Liège 75008 PARIS.

MaaT Pharma a pris toutes les dispositions prévues par la loi relative à la protection des personnes se prêtant à des recherches biomédicales, loi Huriet (n°88-1138) du 20 décembre 1988 modifiée par la loi de santé publique (n°204-806) du 9 août 2004.

All documentation of **MaaT Pharma** is PROPRIETARY information and may not be forwarded to third parties without prior written consent from **MaaT Pharma.** Reproductions, either in part or in whole, may not be published or copied in any manner, without the explicit written consent of **MaaT Pharma**

MaaT Pharma a obtenu l'avis favorable du Comité de Protection des Personnes Ile de France pour cette recherche le 05/04/2016 et une autorisation de l'Agence Nationale de Sécurité du Médicament et des produits de santé (ANSM).

11) Quels sont vos droits ?

Vous avez le droit de refuser de participer à cette étude. Votre décision n'entraînera aucun préjudice sur la qualité des soins et des traitements que vous êtes en droit d'attendre. Vous pourrez tout au long de la recherche demander des explications sur le déroulement de la recherche au médecin qui vous suit.
Vous pouvez vous retirer à tout moment de la recherche sans justification, sans conséquence sur la suite de votre traitement ni la qualité des soins qui vous seront fournis et sans conséquence sur la relation avec votre médecin. A l'issue de ce retrait, vous pourrez être suivi par la même équipe médicale.

Conformément aux dispositions de la CNIL (loi relative à l'informatique, aux fichiers et aux libertés), vous disposez d'un droit d'accès et de rectification. Vous disposez également d'un droit d'opposition à la transmission des données couvertes par le secret professionnel susceptibles d'être utilisées dans le cadre de cette recherche et d'être traitées. Ces droits s'exercent auprès du médecin en charge de la recherche qui seul connaît votre identité. Vous pouvez également accéder directement ou par l'intermédiaire du médecin de votre choix à l'ensemble de vos données médicales en application des dispositions de l'article L1111-7 du Code de la Santé Publique.

Votre dossier médical restera confidentiel et ne pourra être consulté que sous la responsabilité du médecin s'occupant de votre traitement ainsi que par les autorités de santé et par des personnes dûment mandatées par le promoteur MaaT Pharma pour la recherche et soumises au secret professionnel.

A l'issue de la recherche et après analyse des données relatives à cette recherche, vous pourrez être informé(e) des résultats globaux par l'intermédiaire du médecin qui vous suit dans le cadre de cette recherche.

All documentation of **MaaT Pharma** is PROPRIETARY information and may not be forwarded to third parties without prior written consent from **MaaT Pharma.** Reproductions, either in part or in whole, may not be published or copied in any manner, without the explicit written consent of **MaaT Pharma**

Si vous acceptez de participer à la recherche après avoir lu toutes ces informations et discuté tous les aspects avec votre médecin, vous devrez signer et dater le formulaire de consentement éclairé se trouvant à la fin de ce document.

All documentation of **MaaT Pharma** is PROPRIETARY information and may not be forwarded to third parties without prior written consent from **MaaT Pharma.** Reproductions, either in part or in whole, may not be published or copied in any manner, without the explicit written consent of **MaaT Pharma**

PARTICIPATION VOLONTAIRE- CONSENTEMENT ECLAIRE

Je soussigné(e),
Nom …………………………………… Prénom : ……………………………………

Accepte librement et volontairement de participer à l'étude « **ODYSSEE »** dont MaaT Pharma est le promoteur et qui m'a été proposée par le Docteur ………………………………………………., médecin investigateur de l'étude.

En application des dispositions du Code de la Santé Publique, j'ai été informé(e) des objectifs de cette étude, de sa durée, de ses conditions de réalisation, ainsi que de mes conditions de participation.

J'ai pris connaissance du document d'information. J'ai pu disposer d'un temps de réflexion suffisant entre l'information et ma décision de participer à cette étude. J'ai eu l'opportunité d'en discuter avec autrui, de poser des questions complémentaires et j'ai obtenu des réponses satisfaisantes à chacune d'entre elles. Une copie des documents d'information et de recueil de consentement m'a été remise.

J'ai compris que des données me concernant seront récoltées pendant toute ma participation à cette étude, y compris les données concernant mes traitements médicaux et mon état de santé, et que le médecin investigateur et le promoteur de l'étude se portent garant de la confidentialité de ces données.

J'accepte que les données me concernant, recueillies à l'occasion de cette étude, puissent faire l'objet d'un traitement automatisé par les organisateurs de l'essai. Le droit d'accès et de rectification prévu par la loi « Informatique et libertés » s'exerce à tout moment auprès des responsables de l'essai directement ou par l'intermédiaire de votre médecin dans le cadre de l'étude. Le médecin qui me suit s'engage à me fournir à tout moment les informations supplémentaires dont je pourrais avoir besoin.

Je déclare sur l'honneur être affilié à un régime de sécurité sociale ou bénéficiaire d'un tel régime, conformément à l'article L.1121-11 du CSP.
Mon consentement ne décharge en rien l'investigateur et le promoteur de l'ensemble de leurs responsabilités et je conserve tous mes droits garantis par la loi.

De plus, j'accepte que les échantillons de selles et de sang prélevés soient analysés et conservés tel que décrit dans le présent document.

J'ai bien noté qu'à l'issue de la recherche, je serai informé(e) par l'investigateur, à ma demande, des résultats globaux de celle-ci.

All documentation of **MaaT Pharma** is PROPRIETARY information and may not be forwarded to third parties without prior written consent from **MaaT Pharma.** Reproductions, either in part or in whole, may not be published or copied in any manner, without the explicit written consent of **MaaT Pharma**

Fait à ……………………………………………

______________________________ ____________________________________
Signature du patient Date

PARTIE A COMPLETER PAR LE PATIENT

Je soussigné(e), Dr ………………………………………………………………………………………………
Confirme avoir fourni oralement les informations nécessaires sur l'étude et avoir fourni un exemplaire du document d'information au participant.
Je confirme qu'aucune pression n'a été exercée pour que le patient accepte de participer à l'étude et que je suis prêt à répondre à toutes les questions supplémentaires, le cas échéant.

______________________________ ____________________________________
Signature de l'investigateur Date

PARTIE A COMPLETER PAR LE MEDECIN INVESTIGATEUR

Ce formulaire est établi conformément à la loi 88-1138 du 20 décembre 1988 et la loi 2004-806 du 9 aout 2004

Ce document est à réaliser en 2 exemplaires, dont l'original doit être conservé 15 ans par l'investigateur, le 2ème remis à la personne donnant son consentement.

All documentation of **MaaT Pharma** is PROPRIETARY information and may not be forwarded to third parties without prior written consent from **MaaT Pharma.** Reproductions, either in part or in whole, may not be published or copied in any manner, without the explicit written consent of **MaaT Pharma**

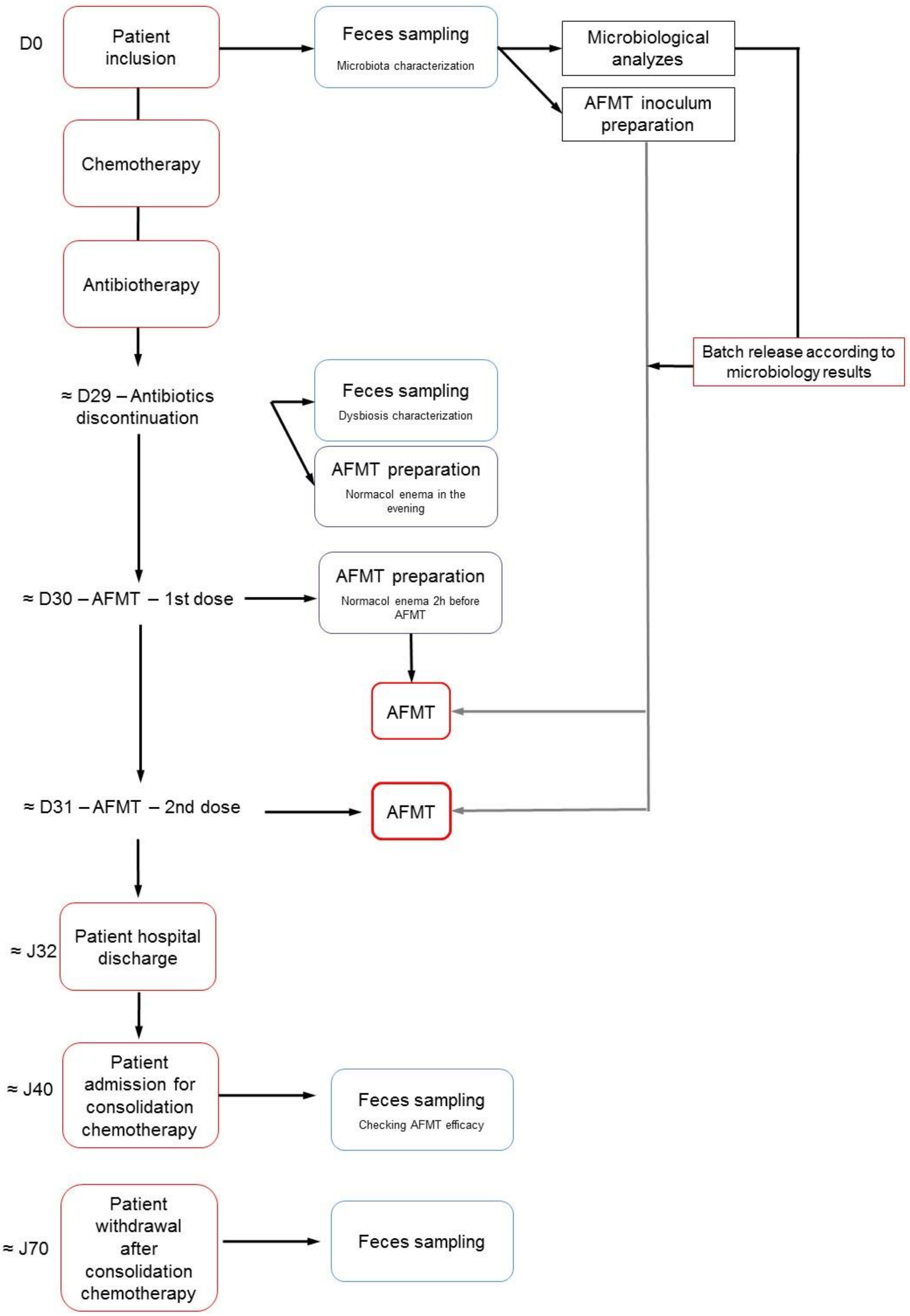

All documentation of **MaaT Pharma** is PROPRIETARY information and may not be forwarded to third parties without prior written consent from **MaaT Pharma.** Reproductions, either in part or in whole, may not be published or copied in any manner, without the explicit written consent of **MaaT Pharma**

Laboratory testing methods

**Biochemistry (feces)**	Calprotectin		ELISA assay
	Zonulin		ELISA assay
	Neopterin		ELISA assay
	IgA		ELISA assay
**Microbiology (feces)**	*C. difficile*		PCR
	Norovirus		PCR
	Rotavirus		Immunochromatography
	MDRB	MRSA	PCR
		VRE et GRE	Culture (2 specific media)
		ESBLs	Culture (2 specific media)
		Carbapenemases	Culture (2 specific media)
	Pathogenic bacteria	*Campylobacter sp*	PCR
		Listeria sp	Culture (ALOA)
		Salmonella sp	PCR
		Shigella sp	PCR
		Vibrio sp	Culture (after enrichment)
		Yersinia sp	Culture (Cefsulodin-Irgasan-Novobiocine)
	Parasites	Strongyloides stercoralis, Cyclospora, Isospora, Entamoeba histolytica, Giardia intestinalis, Cryptosporidium, Microsporidies, Dientamoeba fragilis, Blastocystis hominis	Faeces concentration – coproculture and PCR
**Metagenomic (feces)**			Shotgun (on Illumina HiSeq)
**Immunomonitoring (blood)**	Immune cells	Phenotype	Flow cytometry
	Plasma	Biochemistry	ELISA assays, (Anti)oxidant assays

All documentation of **MaaT Pharma** is PROPRIETARY information and may not be forwarded to third parties without prior written consent from **MaaT Pharma.** Reproductions, either in part or in whole, may not be published or copied in any manner, without the explicit written consent of **MaaT Pharma**
