# Supplementary material for: Gut microbiota diversity after autologous fecal microbiota transfer in acute myeloid leukemia patients
Source: Nat Commun. 2021 May 25;12:3084. doi: 10.1038/s41467-021-23376-6 (PMC8149453; doi:10.1038/s41467-021-23376-6)
Supplement: Supplementary file 2 — Reporting summary [file 41467_2021_23376_MOESM2_ESM.pdf]

nature research

Corresponding author(s): Dr Florent Malard

Last updated by author(s): Jan 21, 2021

Reporting Summary

Nature Research wishes to improve the reproducibility of the work that we publish. This form provides structure for consistency and transparency in reporting. For further information on Nature Research policies, see our Editorial Policies and the Editorial Policy Checklist.

Statistics

For all statistical analyses, confirm that the following items are present in the figure legend, table legend, main text, or Methods section.

n/a	Confirmed	
☐	☒	The exact sample size (*n*) for each experimental group/condition, given as a discrete number and unit of measurement
☐	☒	A statement on whether measurements were taken from distinct samples or whether the same sample was measured repeatedly
☐	☒	The statistical test(s) used AND whether they are one- or two-sided<br>*Only common tests should be described solely by name; describe more complex techniques in the Methods section.*
☒	☐	A description of all covariates tested
☐	☒	A description of any assumptions or corrections, such as tests of normality and adjustment for multiple comparisons
☐	☒	A full description of the statistical parameters including central tendency (e.g. means) or other basic estimates (e.g. regression coefficient) AND variation (e.g. standard deviation) or associated estimates of uncertainty (e.g. confidence intervals)
☐	☒	For null hypothesis testing, the test statistic (e.g. *F*, *t*, *r*) with confidence intervals, effect sizes, degrees of freedom and *P* value noted<br>*Give P values as exact values whenever suitable.*
☒	☐	For Bayesian analysis, information on the choice of priors and Markov chain Monte Carlo settings
☒	☐	For hierarchical and complex designs, identification of the appropriate level for tests and full reporting of outcomes
☒	☐	Estimates of effect sizes (e.g. Cohen's *d*, Pearson's *r*), indicating how they were calculated

Our web collection on statistics for biologists contains articles on many of the points above.

Software and code

Policy information about availability of computer code

Data collection	The CRFs tracking and the data entry tracking have been made by Ennov Clinical®7.5
Data analysis	Statistical analysis performed using R and GraphPad Prism 7.<br>Bioinformatics analyses were performed on Gut Print® platform with the in-house MgRunner v1.1.2 pipeline. In brief, after quality filtering using Trimmomatic v0.36 38, host sequence decontamination was performed using Bowtie2 v2.3.0 39. Taxonomic profiling was performed with Kraken v.0.10.5-beta40 and the RefSeq genomic database (release 84, 2017, http://www.ncbi.nlm.nih.gov/refseq/). The measure of α- and β diversity indexes was performed in R Statistical Software (R Core Team 2015, version 3.4.4, http://www.R-project.org) using vegan v2.4-2 and phyloseq 1.19.1 packages. Gene-based analyses were performed using the Integrated Gene Catalogue (IGC)41. Antibiotic resistance analyses were performed through gene mapping with Bowtie2 v2.3.0 on MEGARes v1.0.1 (https://megares.meglab.org/) database.

For manuscripts utilizing custom algorithms or software that are central to the research but not yet described in published literature, software must be made available to editors and reviewers. We strongly encourage code deposition in a community repository (e.g. GitHub). See the Nature Research guidelines for submitting code & software for further information.

Data

Policy information about availability of data

All manuscripts must include a data availability statement. This statement should provide the following information, where applicable:

- Accession codes, unique identifiers, or web links for publicly available datasets
- A list of figures that have associated raw data
- A description of any restrictions on data availability

The protocol, regulatory documents, and other relevant study materials were submitted to the journal with the manuscript.
Raw data associated with figures 2, 4, 5, 6, S1, S2, S4, S6, S7 and S8 were provided in excel sheet.

Sequence data that support the findings have been deposited in the NCBI SRA database under the BioProject accession PRJNA715586, and available at http://www.ncbi.nlm.nih.gov/bioproject/715586).

Field-specific reporting

Please select the one below that is the best fit for your research. If you are not sure, read the appropriate sections before making your selection.

- [x] Life sciences
- [ ] Behavioural & social sciences
- [ ] Ecological, evolutionary & environmental sciences

For a reference copy of the document with all sections, see nature.com/documents/nr-reporting-summary-flat.pdf

Life sciences study design

All studies must disclose on these points even when the disclosure is negative.

Sample size

Further to recruitment difficulties, the Sponsor wanted to limit the number of cases (35 patients planned initially). It is apparent that small values of effect sizes need at least 44 patients (for effect size = 0.4 and power = 0.25). However, when the effect size is at least 0.6 and with a beta risk of at least 0.20, 20 patients should constitute an acceptable size.

A sample size of 20 patients did not reduce the interest of the study for the main endpoint (correction of dysbiosis). By assuming repeated measurement between Sf (Simpson index at V3) and the two previous S values (Simpson index at V1 (Sb) and V2 (Sd)), it was assumed that the correlation R (St, Sf) was at least R=0.5 with St = (Sb, Sd). It was also considered in this first-in-man trial that a change observed as at least its Sd should be considered as clinically meaningful as defined by Cohen's rules. In these conditions, a sample size of at least 20 patients should provide a power of 0.875 to detect a biologically relevant difference, at a one-tailed significance level of 0.05.

Data exclusions

No data excluded

Replication

No replication was performed as ODYSSEE was a proof-of-concept study evaluating the feasibility and safety of autologous microbiotherapeutic product in Acute Myeloid Leukemia patients.

Randomization

No randomization was performed as ODYSSEE was a single-arm study, evaluating the feasibility and safety of autologous microbiotherapeutic product in Acute Myeloid Leukemia patients

Blinding

No blinding was performed as ODYSSEE was a single-arm study, evaluating the feasibility and safety of autologous microbiotherapeutic product in Acute Myeloid Leukemia patients.

Reporting for specific materials, systems and methods

We require information from authors about some types of materials, experimental systems and methods used in many studies. Here, indicate whether each material, system or method listed is relevant to your study. If you are not sure if a list item applies to your research, read the appropriate section before selecting a response.

Materials & experimental systems

n/a	Involved in the study	
x		Antibodies
x		Eukaryotic cell lines
x		Palaeontology and archaeology
x		Animals and other organisms
	x	Human research participants
	x	Clinical data
x		Dual use research of concern

Methods

n/a	Involved in the study	
x		ChIP-seq
x		Flow cytometry
x		MRI-based neuroimaging

Human research participants

Policy information about studies involving human research participants

Population characteristics	A total of 62 patients between 24 and 69 years of age with de novo diagnosis of AML were screened from 7 French medical centers between June 2016 and July 2017.<br>Patients were aged from 24 to 69 years (mean: 52.8). 57% patients were males, 43% were females. All patients were diagnosed with primary Acute Myeloid Leukemia. In the 25 treated patients, AML risk was favourable for 12%, intermediate for 80% and unfavourable for 8% of patients.
Recruitment	Patients were screened from 7 French medical centers between June 2016 and July 2017. Patients must fulfil all inclusion and exclusion criteria to be included. No potential selection bias was identified.<br>Patient diagnosed with AML/ HR MDS were asked to participate in this study by the investigating physician during his hospitalization for the treatment of AML/ HR MDS or during a consultation at day hospital before hospitalization. Prior to the initiation of any intensive chemotherapy and any antibiotherapy, the patient was examined during a consultation by the physician to ensure that the patient fulfiled inclusion and non-exclusion criteria. The patient was enrolled in the study after receiving oral and written information and giving his consent. Several microbiological analyses, selected according to ANSM recommendations and required for the study were performed on the patient's faeces, collected with the help of a caregiver in special containers and following procedures provided by the sponsor. Collection of faeces was done as soon and as simultaneously as possible after patient's inclusion and before any chemotherapy. If needed, faeces can be collected at patient's home, before hospitalization. In that case, special containers were provided and procedure was explained to the patient during consultation. Blood samples were collected at hospital admission.<br>The patient was given the logbook and the EQ-5D-5L questionnaire to evaluate its gastrointestinal symptoms and its quality of life.<br>The patient was next hospitalized for the beginning of his treatment (induction chemotherapy) and was be clinically and biologically monitored according to the standard procedures of the hematology department. During this period, results from microbiological screenings were delivered to the physician who will confirm if the patient is eligible to AFMT or not.
Ethics oversight	The ODYSSEE study was approved by the ethics committee "Ile de France V" on April 5th, 2016.

Note that full information on the approval of the study protocol must also be provided in the manuscript.

Clinical data

Policy information about clinical studies

All manuscripts should comply with the ICMJEguidelines for publication of clinical research and a completedCONSORT checklist must be included with all submissions.

Clinical trial registration	NCT02928523
Study protocol	Protocol submitted during the submission of the artcile and will be published in supplemental data
Data collection	First patient first visit: 20 JUN 2016 - Last Patient last visit: 25 MAY 2018<br>The data were collected in a paper CRF at investigational sites by the physicians. Paper CRFs were collected by the CRO and entered in the database by data entry operators from the CRO, with a double quality control to ensure the conformity of the data.
Outcomes	Efficacy of AFMT in Dysbiosis Correction/Microbiota Evolution<br>For the primary variable evaluation of AFMT efficacy in dysbiosis correction/microbiota evolution, as this was the first study in humans and as the concept of dysbiosis is not unanimously defined, the Simpson index, already broadly described in the literature, was used as a first approach to focus on microbiota diversity and to evaluate the microbiota evolution/recovery following chemotherapy/antibiotics therapy and AFMT. Microbiome diversity was measured at baseline at genus and species levels, prior to the start of the induction chemotherapy (D0), after induction chemotherapy/antibiotherapy (D29), post-AFMT (D40) and after consolidation chemotherapy/antibiotherapy (D70).<br>Efficacy of AFMT in MDRB eradication:<br>Based on bacterial culture and analysis of resistance gene expression by metagenomics, the quantity of MDRB before (D0), after chemotherapy/antibiotherapy (D29) and after AFMT (D40) was evaluated. The efficacy of AFMT in eradication of MDRB was not demonstrated conclusively in culture - this evaluation was difficult to conduct due to limitations in the culture method. It is perhaps not surprising that few MDRB were detected at D29 since most of the patients had received antibiotics that could limit the MDRB detection.<br>Measurement of Feasibility of the AFMT Procedure<br>The technical feasibility of the AFMT procedure was evaluated in terms of evacuation of each inoculum, and volume loss and retention time of each AFMT.<br>Patient Quality of Life Assessment<br>Health questionnaire EQ-5D-5L (including components on mobility, self-care, usual activities, pain/discomfort, anxiety and depression and overall health) was given to the patient at 4 timepoints (D0, D29, D40 and D70) to evaluate more precisely the impact of AFMT on quality of life.<br>Description of patient clinical status: Haematological response at D29, D40, D70, 6 months' follow-up, 12 months' follow-up and at the end of the study was assessed in terms of CR, PR, overall remission (OR), chemoresistance and progression in the investigator's opinion.<br>Safety Endpoints: Adverse events<br>Pre-AFMT AE data were collected on all 62 patients in the enrolled population, but AE data from subsequent periods were based on the 25 patients in the FAS who all received AFMT. Descriptive statistics were presented for AEs and SAEs by treatment period (pre-

AFMT, within 24 hours of the AFMT and more than 24 hours after AFMT) .
Clinical laboratory evaluation
All individual laboratory measurements were listed by patient. Descriptive statistics of haematology and biochemistry parameters in the FAS population up to D70 were summarised and the evolution of values over time was presented graphically.
Vital signs/physical examinations
Descriptive statistics of vital signs/physical examinations in the FAS population up to D70, and in the period after the AFMTs, were summarised.
